# Reducing Nuclear Rupture Rescues Gene Expression and Survival in *LMNA*-DCM

**DOI:** 10.1161/CIRCRESAHA.125.327744

**Published:** 2026-09-25

**Authors:** Noam Zuela-Sopilniak, Julien L.P. Morival, Ioannis Ntekas, Margaret A. Elpers, Rohit Agarwal, Sarah J. Henretta, Jacob D. Odell, Iwijn De Vlaminck, Jan Lammerding

**Affiliations:** Weill Institute for Cell and Molecular Biology (N.Z.-S., J.L.P.M., M.A.E., S.J.H., J.D.O., J.L.), Cornell University 5922 , Ithaca, NY.; Meinig School of Biomedical Engineering (N.Z.-S., J.L.P.M., I.N., M.A.E., R.A., S.J.H., J.D.O., I.D.V., J.L.), Cornell University 5922 , Ithaca, NY.

**Keywords:** fibrosis, inflammation, myocytes, cardiac, nuclear envelope, transcriptome

## Abstract

**BACKGROUND:**

Mutations in the *LMNA* gene, which encodes the nuclear envelope proteins lamins A and C, account for 6% to 8% of all cases of congenital dilated cardiomyopathy. *LMNA*-related dilated cardiomyopathy is among the most severe forms of dilated cardiomyopathy, with limited treatment options due to an incomplete understanding of its molecular mechanisms.

**METHODS:**

We generated an inducible, cardiomyocyte-specific *Lmna* deletion mouse model (cKO) and conducted comprehensive bulk, single-nucleus, and spatial transcriptomic analyses on left ventricular free wall tissue from cKO and controls lacking the floxed *Lmna* alleles across disease progression. Concurrent cardiomyocyte-specific expression of a dominant-negative KASH domain protein (DN KASH) that disrupts the linker of nucleoskeleton and cytoskeleton complex was used to determine the role of mechanically induced nuclear envelope rupture in altered gene expression.

**RESULTS:**

We identified genes misregulated early in disease that were enriched for cytosolic pattern recognition receptor signaling and innate immunity. These transcriptional changes were primarily driven by a subset of disease-specific cardiomyocytes. Spatial and single-nucleus transcriptomics revealed aberrant interactions between these cardiomyocytes, fibroblasts, and immune cells, contributing to widespread transcriptional dysregulation in cKO hearts. Concurrent disruption of the linker of nucleoskeleton and cytoskeleton complex significantly reduced nuclear envelope rupture, normalized expression of over half the dysregulated genes, and dramatically improved cardiac function and survival in cKO mice.

**CONCLUSIONS:**

Our findings support a model in which nuclear envelope rupture in cKO cardiomyocytes triggers cytosolic DNA sensing pathways and maladaptive cell-cell communication with fibroblasts and immune cells, driving inflammation, fibrosis, and cardiac dysfunction. The work presented here points to novel strategies for treating *LMNA*-dilated cardiomyopathy.

**Novelty and Significance**

**What Is Known?**

- Loss of the nuclear envelope proteins lamin A/C in cardiomyocytes is sufficient to cause *LMNA*-related dilated cardiomyopathy (*LMNA*-DCM), which currently lacks disease-modifying therapies.
- Loss of lamin A/C results in perturbed gene expression in cardiomyocytes and increased nuclear envelope (NE) rupture.
- Whether *LMNA*-DCM arises from altered gene regulation or from NE rupture has remained unresolved.

**What New Information Does This Article Contribute?**

- Transcriptional changes in *LMNA*-DCM mouse hearts are concentrated in 2 subpopulations of cardiomyocytes that are highly enriched for markers of NE rupture and exhibit aberrant signaling to myofibroblasts and macrophages.
- NE rupture is associated with increased pattern recognition receptor signaling that drives innate immune activation and fibrosis.
- Reducing NE rupture by disrupting the linker of nucleoskeleton and cytoskeleton complex normalizes expression of more than half of all dysregulated genes in *LMNA*-DCM hearts, largely restores cardiac function, and significantly extends survival, despite continued lamin A/C depletion.

Mutations in the *LMNA* gene, encoding the NE proteins lamin A/C, cause a severe form of inherited *LMNA*-DCM, for which no targeted therapies exist. Progress has been slowed by a long-standing ambiguity: lamin A/C both organize chromatin and mechanically protect the nucleus, leaving it unclear whether cardiac dysfunction reflects a primary defect in transcriptional regulation or the consequence of mechanically induced NE rupture in cardiomyocytes. This distinction matters because the 2 models point toward different therapeutic strategies. Applying bulk, single-nucleus, and spatial transcriptomics to mice with inducible, cardiomyocyte-specific lamin A/C depletion, we provide evidence that NE rupture is responsible for many of the transcriptomic changes in *LMNA*-DCM hearts, including innate immune activation that promotes inflammation and fibrosis. Critically, reducing NE rupture by linker of nucleoskeleton and cytoskeleton complex disruption was sufficient to substantially rescue gene expression, cardiac function, and survival, despite continued lamin A/C depletion. Our findings indicate that NE rupture is a causal disease driver and that much of the abnormal gene expression arises downstream of NE rupture rather than from defects in transcriptional regulation. Targeting NE rupture, or the pathogenic signaling it triggers, may therefore offer therapeutic promise in *LMNA*-DCM and other diseases involving NE damage.

**Meet the First Author, see p e000772**

**Editorial, see Article by Chen et al**

Lamins A and C are nuclear intermediate filaments that provide structural support to the nucleus and regulate numerous cellular processes, including chromatin organization and transcriptional regulation. Although lamins A/C are nearly ubiquitously expressed, most *LMNA* mutations primarily affect striated muscle and tendons. *LMNA*-related dilated cardiomyopathy (*LMNA*-DCM) is the most prevalent laminopathy and has a worse clinical prognosis compared with other congenital DCMs,^1,2^ frequently resulting in sudden cardiac death.^1^ Currently, no effective therapies exist for *LMNA*-DCM, and the molecular pathogenic mechanism remains largely unresolved, presenting a major hurdle in developing effective treatments.

A major question regarding striated muscle laminopathies is whether tissue-specific phenotypes arise from perturbed chromatin organization and altered transcriptional regulation (gene regulation hypothesis) or from impaired nuclear structural stability, resulting in nuclear envelope (NE) rupture in mechanically stressed tissues (structural hypothesis).^3,4^ Supporting the structural hypothesis, *LMNA* mutations linked to striated muscle disease often disrupt lamin filament assembly, resulting in mechanically fragile nuclei and increased NE rupture.^5–7^ NE rupture, although typically repaired rapidly,^8^ can lead to DNA damage and activate cytosolic DNA sensing pathways, such as cGAS (cyclic GMP-AMP synthase)/STING (Stimulator of Interferon Response cGAMP Interactor),^5,9^ potentially triggering pathogenic signaling cascades that contribute to cardiac dysfunction. However, whether NE ruptures directly drive cardiac dysfunction in *LMNA*-DCM remains unclear.

Previous studies have identified several altered signaling pathways in *LMNA*-DCM, including MRTF (myocardin-related transcription factor), ERK1/2 (extracellular-regulated kinases 1 and 2), AKT (protein kinase B)/mTOR (mammalian target of rapamycin), Wnt/β-Catenin, and DNA damage response pathways.^10,11^ However, despite efforts to target these pathways in preclinical models, none have been curative, and most have shown only modest survival benefits,^11^ raising the question of whether these pathways are disease-causing or represent downstream consequences of other pathogenic effects.

Here, we addressed these questions using a multilevel transcriptomic analysis of left ventricular tissue from an inducible cardiomyocyte-specific *Lmna* deletion mouse model (cKO). By examining early transcriptional changes that intensify with disease progression, we identified activation of cGAS-independent cytosolic pattern recognition receptor (PRR) pathways, likely triggered by exposure of DNA to the cytoplasm following NE rupture,^12^ which promote innate immune activation and sterile inflammation. Single-nucleus RNA sequencing (snRNA-seq) and spatial transcriptomics identified 2 disease-specific cardiomyocyte subpopulations largely responsible for tissue-level transcriptomic changes and activation of fibroblasts and innate immune cells. Supporting a causal role of NE rupture, concurrent cardiomyocyte-specific disruption of the linker of nucleoskeleton and cytoskeleton (LINC) complex,^5,6^ which reduces physical stress on cardiomyocyte nuclei,^6,13,14^ reduced NE ruptures and rescued the expression of >50% of dysregulated genes, including PRR and inflammation molecular signatures. Notably, LINC complex disruption resulted in near-complete rescue of cardiac function and extended survival of cKO mice from weeks to over 1 year. Together, our findings offer new mechanistic insight into *LMNA*-DCM pathogenesis and identify promising therapeutic targets.

## Methods

### Data Availability

Bulk RNA sequencing (RNA-seq; GSE269451 and GSE304346), snRNA-seq (GSE269452), and Spatial Transcriptomics (GSE307910) data sets are publicly available in the Gene Expression Omnibus (GEO) repository.

A detailed version of the methods is available in the Supplemental Methods.

### Animals

Transgenic mice expressing alpha-myosin heavy chain (αMHC)-MerCreMer (JAX 005657), *Lmna* floxed alleles (JAX 026284), or a Cre-inducible dominant-negative KASH domain protein (DN KASH) construct^15^ were maintained in a C57BL/6 background. Conditional *Lmna* deletion (cKO) and combined *Lmna* deletion with LINC disruption (cKO + DN KASH) were generated through standard breeding. Tamoxifen (30 mg/kg, 5 days) was administered at 9 to 10 weeks by intraperitoneal injections to induce cardiomyocyte lamin A/C depletion and LINC complex disruption. αMHC-MerCreMer hemizygous *Lmna*^+/+^ littermates served as controls lacking the floxed *Lmna* alleles (cWT). All procedures followed institutionally approved IACUC protocols.

### Cardiac Function

Echocardiography was performed under isoflurane anesthesia using the Vevo 2100 system. Left ventricular systolic function was quantified from AM-mode images over ≥5 cardiac cycles by investigators blinded to genotype and treatment.

### Immunofluorescence and Histology of Mouse Tissues

Hearts were fixed in 4% paraformaldehyde before paraffin embedding for histology, or flash frozen in OCT and cryosectioned for immunofluorescence. Masson trichrome staining and immunofluorescence were performed using standard protocols. Images were acquired and processed using consistent exposure and threshold parameters.

### Proximity Image Analysis

CellProfiler^16^ was used to segment 4′,6-diamidino-2-phenylindole (DAPI)-positive nuclei, CD68-positive and reticulon 4 (RTN4)-positive regions, and to compute nearest neighbor distances between CD68-positive regions and the nearest RTN4-positive regions.

### Western Blot Analysis

Heart lysates were prepared in high-salt radioimmunoprecipitation assay buffer (RIPA) buffer, resolved by SDS-PAGE, transferred to polyvinylidene difluoride (PVDF) membranes, and probed for CD68 and RTN4. Detection used IRDye secondary antibodies and LI-COR imaging.

### Bulk RNA-Seq

Poly(A)-selected RNA from left ventricles was used for library preparation and sequencing. We used 24 cKO and cWT total samples (data set GSE269451) for the initial comparison between cKO and cWT hearts, and 48 total samples from the LINC complex disruption anaylsis (data set GSE304346). Reads were aligned with STAR^17^ to GRCm38/mm10, quantified with featureCounts,^18^ and analyzed using DESeq2.^19^ Differentially expressed genes (DEGs) were defined using |log_2_FC| ≥1 and *P*-adj ≤0.05. Emerging DEGs (eDEGs) were identified by comparing 11 days post-injection (dpi) genes with *P*-adj <0.05 and base mean expression ≥10 to 25-dpi DEGs with consistent directionality (GSE269451, Figure S2A; GSE304346, Figure S20A).

### Single-Nucleus RNA Sequencing

Nuclei from frozen left ventricle samples were processed using the 10× Genomics Chromium 3′ workflow (SingulOmics). Cell Ranger^20^ output was filtered using Debris Identification using Expectation Maximization (DIEM)^21^ and analyzed with Seurat,^22^ including ambient RNA and doublet removal, sctransform normalization, integration, and clustering. Cell types were annotated using Azimuth.^23–25^ Cluster-specific DEGs were identified with the Seurat FindMarkers function, and gene module scores were calculated using the Seurat AddModuleScore function.

### Cell-Cell Interaction Prediction

Ligand-receptor interactions were inferred using CellChat^26^ (default settings) and compared across genotypes and time points.

### Pseudotime Analysis

Cardiomyocyte lineage trajectories were inferred using Slingshot^27^ on principal component analysis-derived embeddings using CM1 as the root state and CM3 as the terminal state.

### Functional Enrichment Analysis

Ranked DEG and eDEG lists were analyzed using Metascape^28^ and Ingenuity Pathway Analysis^29^ for gene ontology (GO) pathway enrichment and upstream regulator inference.

### Human DCM snRNA-Seq Data Set

Publicly available snRNA-seq data of congenital DCM^30^ were downloaded from CZ CELLxGENE,^31^ processed using Scanpy,^32^ and analyzed with the standard Seurat pipeline.

### Spatial Transcriptomics

Heart sections were processed on Curio Seeker arrays. Data were aligned to GRCm39 using STARsolo^33^ and preprocessed with Scanpy,^32^ including bead filtering, normalization, spatial smoothing, and left ventricle (LV) segmentation. Cell2location^34^ and Squidpy^35^ were used for spatial analyses including deconvolution, and subpopulation and neighborhood enrichment.

### Statistical Analysis

Animals were randomly assigned and analyses were performed blinded. Appropriate parametric or nonparametric tests were applied based on sample distribution.

## Results

### Cardiomyocyte-Specific Depletion of Lamin A/C in Adult Mice Causes Severe DCM

We generated cKO mice using a tamoxifen-responsive Cre driven by the α-myosin heavy chain promoter and floxed *Lmna* alleles. Inducing lamin A/C depletion in adulthood allows separation of effects on cardiac function from those during development. In addition, this model minimizes confounding factors present in other *LMNA*-DCM models, including variability in disease onset and severity, multitissue involvement, and differences in animal size and motility.^36^ Following tamoxifen treatment (Figure 1A), lamin A levels in cKO cardiomyocytes were significantly reduced by ≈50% to 70% at 11-dpi, compared with tamoxifen-treated littermate cWT (Figure 1B and 1C) and vehicle-treated cKO controls (Figure S1A). This depletion matches the reduced lamin A/C levels often seen in human *LMNA*-DCM hearts.^37^ Immunofluorescence for lamin A/C showed a similar but smaller effect size relative to lamin A (Figure S1B and S1C). Since the lamin A antibody produced better labeling and matched a recent study in a similar cKO model,^38^ subsequent analyses are based on lamin A immunofluorescence. Despite the only partial lamin A/C depletion, tamoxifen-treated cKO mice rapidly developed severe DCM (Figure 1D). Histological analysis revealed a visible increase in fibrosis at 11-dpi, followed by substantial left ventricular enlargement by 25-dpi in cKO compared with cWT hearts (Figure 1D and 1E; Figure S1D). Echocardiography showed a significant reduction in cardiac function in tamoxifen-treated cKO mice by 25-dpi (Figure 1F; Figure S1E through S1H), whereas tamoxifen-treated cWT and vehicle-treated cKO controls maintained normal cardiac morphology and function (Figure 1D through 1F; Figure S1D through S1H).

**Figure 1.**
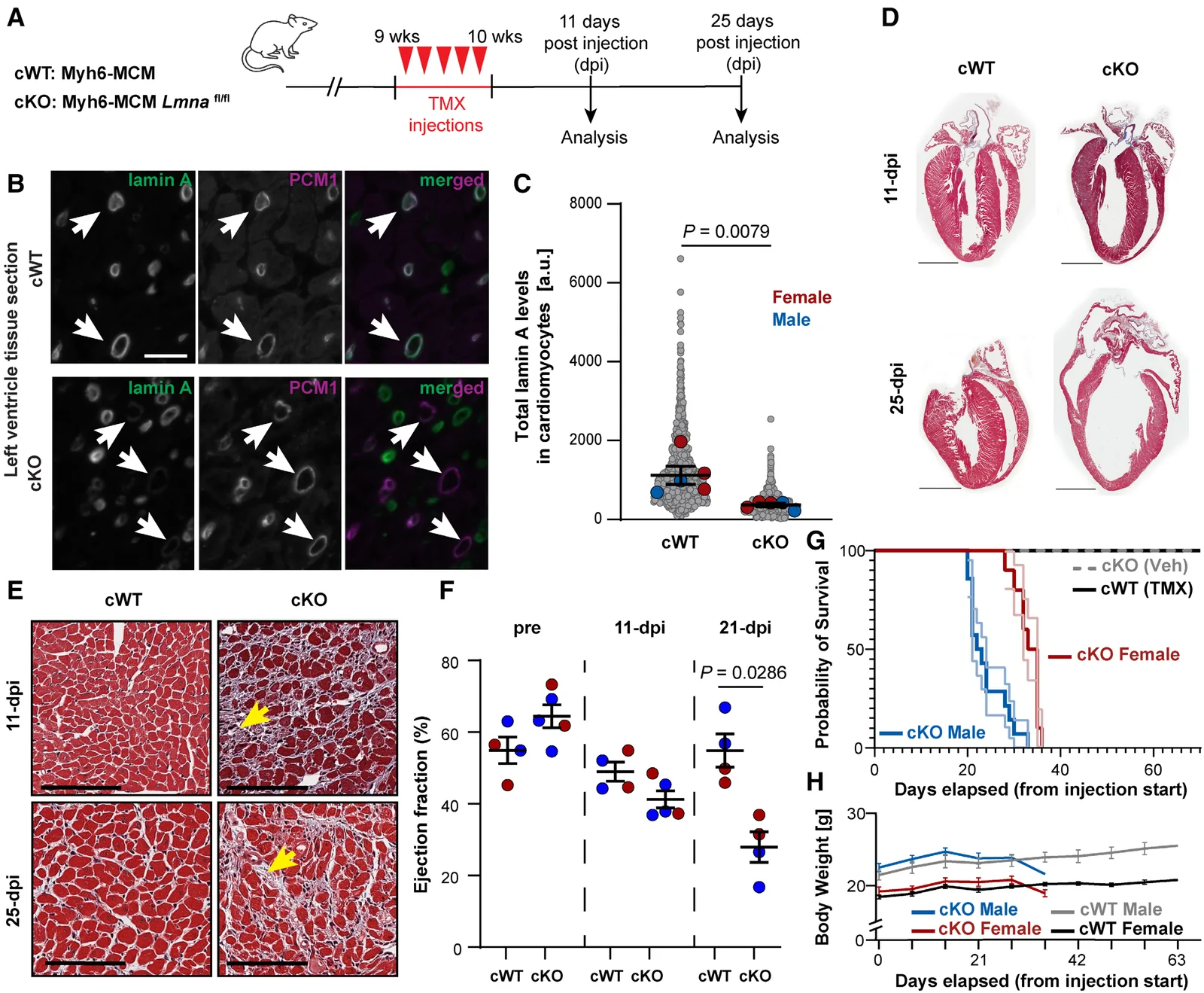
**Cardiac-specific lamin A depletion causes rapid-onset dilated cardiomyopathy. A**, Experimental timeline for tamoxifen-induced *Lmna* deletion with echocardiography and tissue collection at indicated time points. **B**, Representative immunofluorescence images of cardiac tissues from cWT (**top**) and cKO (**bottom**) mice labeled for lamin A (green) and pericentriolar material 1 (PCM1, magenta), a cardiomyocyte-specific nuclear marker. Scale bar, 20 µm. Arrows indicate representative cardiomyocytes. **C**, Quantification of lamin A levels in cardiomyocytes. Colored circles represent average values for individual male (blue) or female (red) mice. Gray circles represent individual cardiomyocytes. N=5 animals/genotype. **D** and **E**, Masson trichrome staining of heart sections (**D**) showing progressive thinning of the left ventricular free wall and enlargement of cKO hearts. Scale bar, 2 mm, and (**E**) increased collagen deposition (yellow arrows) in cKO hearts at both 11- and 25-days post-injection (dpi). Scale bar, 100 µm. **F**, Left ventricular ejection fraction (EF) measured by echocardiography. Symbols represent %EF of individual (red for female and blue for male) mice. Error bars represent mean ± SEM. N=4 to 5 animals/genotype. **G**, Kaplan-Meier survival plot for tamoxifen-treated cWT mice (N=14 males and 11 females), vehicle-only-treated cKO mice (N=10 males and 2 females), and tamoxifen-treated cKO mice (N=14 males and 10 females). **H**, Body weight measurements performed during the survival study on the mice in panel (**G**) before (day 0) and after tamoxifen injections. Statistical tests: (**C**) Mann-Whitney *U* test and (**F**) Mann-Whitney *U* test for each time point. cKO indicates inducible cardiomyocyte-specific *Lmna* deletion mouse model; cWT, controls lacking the floxed *Lmna* alleles; and TMX, tamoxifen.

Consistent with their severe cardiac functional decline, tamoxifen-treated cKO mice had significantly reduced survival compared with cWT and vehicle-treated controls, with a median survival of <5 weeks (Figure 1G). Male cKO mice died earlier than females, despite similar lamin A depletion (Figure 1C), consistent with previous reports in other *LMNA*-DCM models.^39^ Tamoxifen-treated cWT controls showed no premature mortality (Figure 1G), indicating minimal cardiotoxic effects of the Cre recombinase. Unlike other striated laminopathy models,^40^ cKO mice died suddenly, without preceding weight loss (Figure 1H) or external indications, closely mirroring the sudden cardiac death in many *LMNA*-DCM patients.^41^ Collectively, these findings indicate that even partial reduction of lamin A/C protein levels in cardiomyocytes is sufficient to cause severe, rapidly progressing DCM.^37,42^

### Transcriptomic Profiling Reveals Early and Progressive Gene Dysregulation in cKO Hearts

To characterize transcriptional changes driven by cardiac-specific lamin A/C depletion, we performed bulk RNA-seq, snRNA-seq, and spatial transcriptomics on left ventricles from tamoxifen-treated cKO and cWT mice at 11- and 25-dpi (Figure 2A), corresponding to early and late disease states, respectively (Figure 1D through 1F). We initially focused on bulk RNA-seq to capture global transcriptional changes and associated pathways. Transcriptional differences between cKO and cWT hearts were detectable at 11-dpi and became more pronounced by 25-dpi (Figure 2B), with DEGs increasing from 86 to 2951 (Figure S2A). Most DEGs were significantly upregulated in cKO hearts (Figure S2B), suggesting progressive transcriptional program activation. Despite sex-specific survival differences (Figure 1G), principal component analysis showed no segregation by sex (Figure S3A); therefore, we combined data from both sexes for subsequent analyses to increase statistical power.

**Figure 2.**
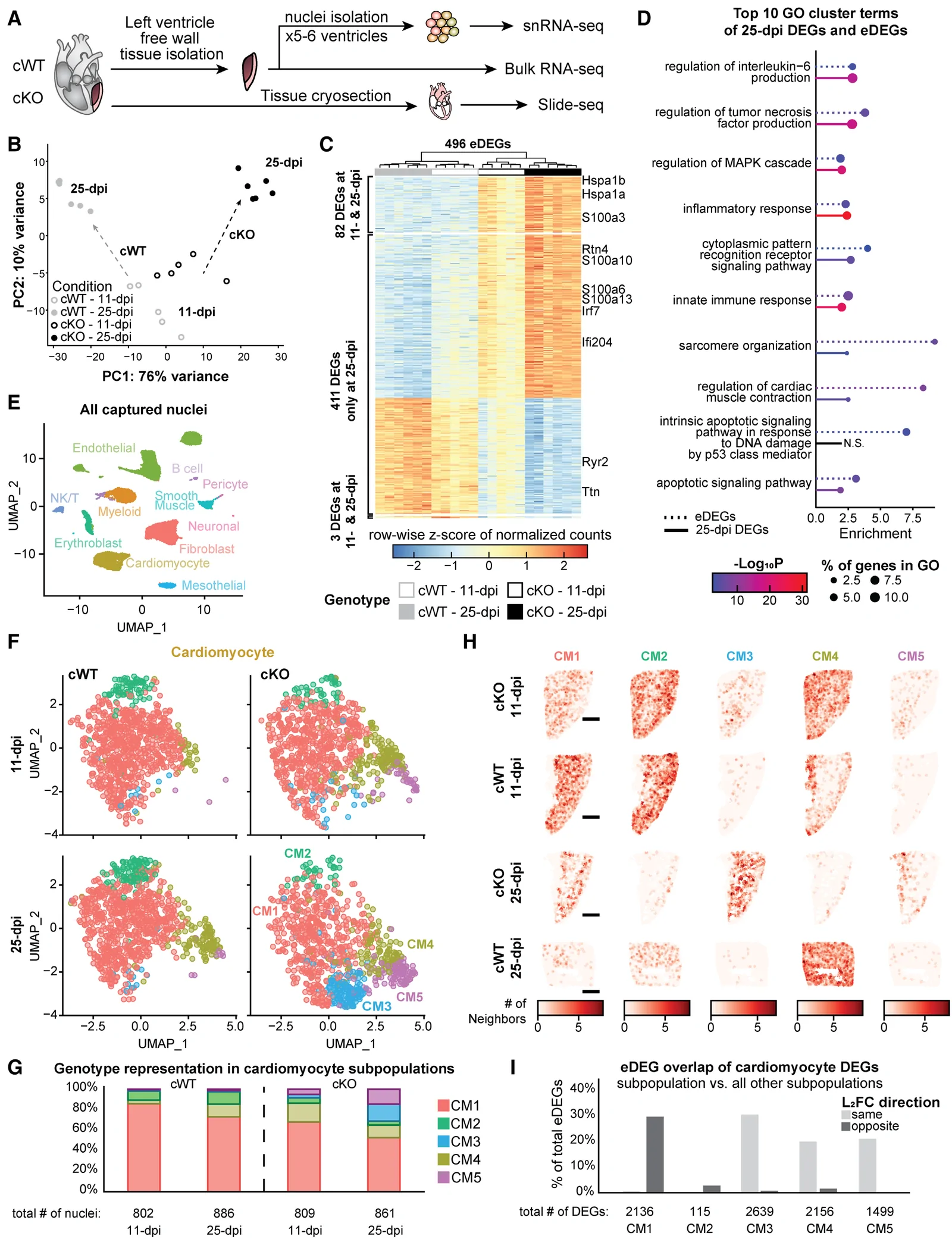
**Two disease-specific cardiomyocyte subpopulations account for a large fraction of emerging differentially expressed gene (eDEG) expression in cKO hearts. A**, Experimental design for transcriptomic analyses of cKO and cWT hearts, including bulk RNA sequencing (RNA-seq; N=5–6 animals/genotype), single-nucleus RNA sequencing (snRNA-seq; N=5–6 animals/genotype), and spatial transcriptomics (N=1 animal/genotype). **B**, Principal component analysis (PCA) for bulk RNA-seq samples reveals progressive genotype-dependent separation of samples from 11- to 25-days post-injection (dpi; arrows). **C**, Heat map of row-wise *Z*-score-normalized read counts for eDEGs. Genes meeting DEG criteria at both time points (85 genes, **top** and **bottom** sections) or emerging by 25-dpi (411 genes, **middle** section) are indicated. Samples are hierarchically clustered. Select genes identified throughout the study are highlighted. **D**, Gene ontology (GO) cluster term enrichment of eDEGs (dashed) and 25-dpi differentially expressed genes (DEGs; solid). **E**, Uniform Manifold Approximation and Projection (UMAP) of integrated snRNA-seq nuclei from cWT (**left**) and cKO (**right**) hearts, colored by cell type and shaded by time point (11-dpi, light; 25-dpi, dark). **(F**) UMAP of cardiomyocyte (CM) nuclei, colored by subpopulation (CM1–CM5), across genotype (**left** and **right**) and time point (**top** and **bottom**). **G**, Relative composition of cardiomyocyte subpopulations shown as the percentage of total cardiomyocyte nuclei sequenced per condition. Total numbers of sequenced nuclei are indicated for each subgroup. **H**, Spatial neighborhood enrichment maps of cardiomyocyte subpopulations in left ventricular sections from cKO and cWT hearts at 11- and 25-dpi. Red intensity indicates local enrichment. Scale bars, 500 µm. **I**, Percentage of eDEGs overlapping with subpopulation-specific DEGs. Bar color indicates concordant (both up- or downregulated; light gray) or discordant (1 up- and 1 downregulated; dark gray) fold-change direction of corresponding eDEG and DEG. The total number of DEGs is indicated below each condition. cKO indicates inducible cardiomyocyte-specific *Lmna* deletion mouse model; cWT, controls lacking the floxed *Lmna* alleles; MAPK, mitogen-activated protein kinase; NS, not significant; and PC, principal component.

Given the subtle transcriptional differences at 11-dpi, we reasoned that early disease-relevant gene expression changes may fall below our stringent DEG threshold (|log_2_-fold change| ≥1) yet may still contribute to pathogenesis. We developed an analysis pipeline that identified 496 genes that already differed significantly between cKO and cWT at 11-dpi and whose change in expression reached the DEG threshold by 25-dpi (Figure S2A). We termed these genes eDEGs (Table S2). Hierarchical clustering based on eDEG expression separated samples by genotype and time point (Figure 2C), demonstrating effective detection of early transcriptional changes. In contrast to the 496 eDEGs, only 12 genes identified as significantly different at 11-dpi reversed expression direction at 25-dpi (Figure S2C and S2D).

To identify biological processes underlying the transcriptional changes in cKO hearts, we performed comparative GO analysis of eDEGs and 25-dpi DEGs (Figure 2D).^28,43^ Both gene sets were enriched for pathways previously linked to lamin A/C deficiency, including MAPK activation^44^ and apoptosis.^45^ Notably, eDEGs showed additional enrichment for inflammation, innate immunity, and cardiac function, suggesting their activation early in disease.

### Two Cardiomyocyte Subpopulations Predominantly Account for eDEG Expression in cKO Hearts

To identify cell-type specific transcriptional contribution, we focused on the snRNA-seq data set. Cell type annotation identified the expected left ventricular cell types^30^ (Figure 2E). Cardiomyocyte nuclei clustered into 5 subpopulations (Table S4), 2 of which, CM3 and CM5, were exclusive to cKO hearts and progressively expanded over time (Figure 2F and 2G), mirroring the increased transcriptional divergence between cKO and cWT hearts (Figure 2B). Spatial transcriptomics analysis confirmed the presence of CM3 and CM5 in cKO hearts at both 11- and 25-dpi, but not in cWT hearts (Figure 2H; Figure S4).

We next compared transcriptomic profiles of individual subpopulations with the eDEGs identified by bulk RNA-seq. CM3 and CM5 together accounted for over 40% of all eDEGs (Figure 2I; Figure S5A and S5B), despite representing only 20% of cardiomyocyte nuclei in cKO hearts (Figure 2G) and <5% of all captured nuclei. The eDEGs enriched in CM3 and CM5 were associated with GO terms related to immunity, apoptosis, and cardiac function (Figure S5C), suggesting that the CM3/CM5 subpopulations disproportionately contribute to *LMNA*-DCM pathogenesis.

### cKO Cardiomyocytes Exhibit Transcriptomic Evidence of Cytosolic DNA Sensing Pathway Activation

Cells from human patients and mouse models of striated laminopathies frequently exhibit nuclear damage, including chromatin protrusions and NE ruptures.^5,38,46–48^ NE ruptures can be visualized by transient accumulation of barrier-to-autointegration factor (BAF) at the NE,^49^ where BAF facilitates NE repair.^8^ BAF staining revealed that cKO, but not cWT, cardiomyocytes frequently exhibited NE ruptures (Figure 3A; Figure S6). Consistent with cytoplasmic exposure to DNA, eDEGs were significantly enriched for the GO term cytoplasmic PRR signaling pathway. This term includes cytosolic DNA sensing molecules such as cGAS and its downstream target STING, a central regulator of inflammation via activation of NF-κB (nuclear factor kappa B) and IRFs (interferon-regulatory factors).^12,50^ Ingenuity Pathway Analysis further predicted multiple other components of cytosolic DNA-sensing as upstream regulators of eDEGs, including STING1, IRF3, IRF7, MyD88, and NF-κB (Table S1). While GO-term analysis of eDEGs associated with CM3 and CM5 did not show direct enrichment of cytosolic DNA sensing pathway (Figure S5C), Ingenuity Pathway Analysis identified several related upstream regulators, including TLR9 (toll-like receptor-9), p53 (TP53), IL6 (interleukin-6), NF-κB, IL1A/B (interleukin-1), MyD88, and IFNG (interferon-γ; Figure 3B). Furthermore, both snRNA-seq and spatial data revealed enrichment of inflammatory response and regulation of defense GO terms, which include cytosolic DNA-sensing molecules, within CM3 and CM5 cardiomyocytes (Figure 3C through 3E; Figure S7A and S7B).

**Figure 3.**
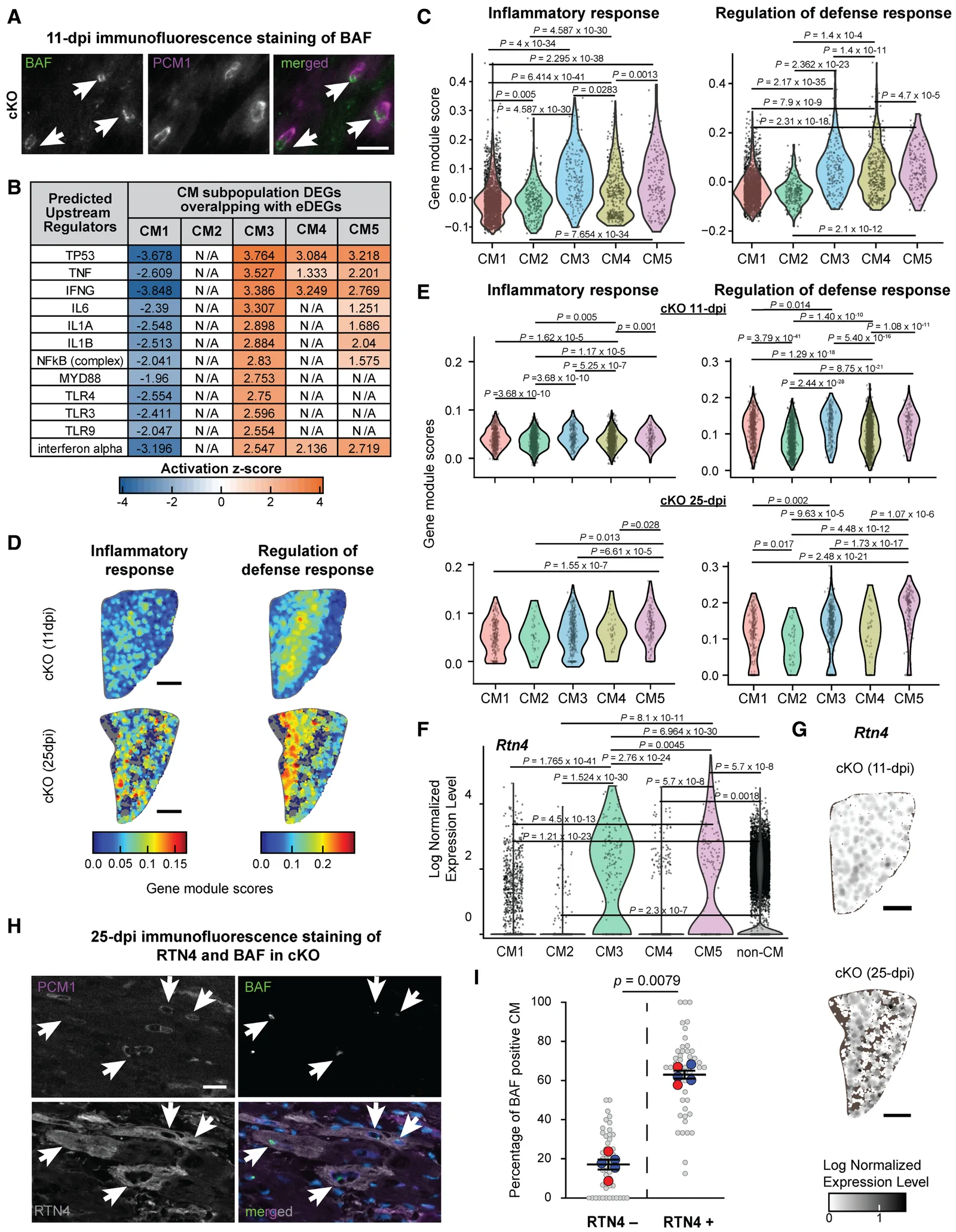
**cKO hearts exhibit transcriptomic signatures of cytosolic DNA-sensing pathway activation. A**, Immunofluorescence images of cardiac tissues from cKO mice at 11-days post-injection (dpi) labeled for barrier-to-autointegration factor (BAF; green) to indicate nuclear envelope (NE) rupture, and pericentriolar material 1 (PCM1; magenta) to identify cardiomyocytes. Scale bar, 20 µm. Arrows indicate representative cardiomyocytes. **B**, Predicted activation of the top 40 upstream regulators involved in DNA damage and cytosolic DNA sensing pathways identified using Ingenuity Pathway Analysis (IPA) based on cardiomyocyte subpopulation (CM1–CM5)-specific differentially expressed genes (DEGs) that overlap emerging DEGs (eDEGs). N/A indicates the upstream regulator was not predicted. **C**, Gene module scores for gene ontology (GO) terms inflammatory response (**left**) and regulation of defense (**right**) for cardiomyocyte subpopulations in the integrated single-nucleus RNA sequencing (snRNA-seq) data set. **D**, Spatial distribution and (**E**) quantification of GO term inflammatory response (**left**) and regulation of defense (**right**) gene module scores across the (**D**) left ventricle and (**E**) cardiomyocyte subpopulations at 11- and 25-dpi in cKO hearts. **F**, Normalized* reticulon 4* (*Rtn4*) expression across CM1–CM5 and all noncardiomyocyte cells in the integrated snRNA-seq data set. **G**, Spatial expression of *Rtn4* in cKO left ventricles at 11-dpi (**left**) and 25-dpi (**right**). **H**, Immunofluorescence labeling for RTN4 (gray), BAF (green), PCM1 (magenta), and nuclei using 4′,6-diamidino-2-phenylindole (DAPI, blue) for cKO left ventricles. Scale bar, 20 µm. **I**, Quantification of BAF-positive nuclei in RTN4-negative (**left**) and RTN4-positive (**right**) cardiomyocytes in cKO left ventricles at 25-dpi. Statistical annotation: Colored circles represent average values for individual male (blue) or female (red) mice. N=5 animals/group. **C**, **E**, and **F**, Gray dots represent individual nuclei (**C** and **F**) and array spots (**E**). Statistical tests: (**C**, **E**, and **F**) pair-wise Wilcoxon rank-sum test with Benjamini-Hochberg correction, and (**I**) Mann-Whitney *U* test. cKO indicates inducible cardiomyocyte-specific *Lmna* deletion mouse model; IFNG, interferon-gamma; IL, interleukin; NFkB, nuclear factor kappa B; TNF, tumor necrosis factor; and TLR, toll-like receptor.

Despite the activation of cytosolic DNA-sensing pathways, only a few cardiomyocytes expressed *cGAS* or *Sting* (0.35% and 0.2%, respectively), with no detectable expression in CM3 and CM5 (Figure S8A through S8D). Although low transcript abundance precludes definitive exclusion, the rare detection frequency aligns with recent reports that cGAS is essentially absent in cardiomyocytes, and that deletion of *cGas* or *Sting* does not rescue *LMNA*-DCM.^30,38,51^ We therefore investigated alternative cytosolic DNA sensing mechanisms. *Rtn4* stood out as a highly upregulated eDEG in CM3 and CM5 relative to other cardiomyocytes (Figure 3F and 3G; Figure S7C). RTN4 is an endoplasmic reticulum transmembrane protein that regulates trafficking of toll-like receptors (TLRs), particularly TLR9, to endolysosomes, where they initiate cGAS/STING-independent innate immune signaling.^12,52^ We confirmed elevated RTN4 levels in cKO versus cWT hearts at 25-dpi by immunofluorescence (Figure S9) and immunoblot analyses (Figure S10A). We also observed significant upregulation of genes downstream of TLR signaling (*Traf3*, *Tkb1*, *Irak4*, *and Map3k7*) in CM3 and CM5 relative to other cardiomyocytes (Figure S11).

These findings indicate that CM3 and CM5 represent cardiomyocytes with damaged nuclei. Costaining for PCM1 (cardiomyocyte marker), RTN4 (CM3/CM5 biomarker), and BAF (ruptured nuclei) showed that RTN4-positive cardiomyocytes were significantly enriched for BAF-positive nuclei (64% versus 17% in RNT4-negative cells), demonstrating strong association between NE rupture and RTN4 upregulation (Figure 3H and 3I). In addition to TLR-associated signaling, we found evidence of STING-independent cytosolic DNA sensing in CM3 and CM5, including upregulation of *Dhx9* (Figure S8E and S8F), a DNA-binding helicase that activates NF-κB and transforming growth factor beta (TGF-β),^53^ and *Hmgb1* (Figure S11), a cytosolic DNA-binding TLR accessory protein.^53^ Furthermore, many cardiomyocytes, particularly in CM3, expressed *IFI16*/*Ifi204* (interferon gamma inducible protein 16; Figure S8G and S8H), a cytoplasmic DNA sensor capable of inducing inflammasome formation and cytokine release independently of STING.^54^

Collectively, these findings suggest that CM3/CM5 represent cardiomyocytes in which NE rupture activates cGAS/STING-independent cytosolic DNA-sensing pathways, driving inflammatory signaling and cytokine production. Supporting this interpretation, we identified similar significant upregulation of PRR-associated genes in transcriptomic data of human *LMNA*-DCM hearts,^30^ including *RTN4*, *MAP3K7*, and *HMGB1*, compared with healthy controls (Figure S12).

### cKO Hearts Show Progressive Innate Immune Cell Activation

Cardiomyocytes respond to injury by releasing cytokines and damage-associated molecular patterns (DAMPs),^30,38^ which recruit immune cells and promote inflammation and cardiac remodeling.^55,56^ Accordingly, GO term analysis of eDEGs revealed enrichment for immune and inflammatory pathways, particularly innate immunity (Figure 2D). CM3 and CM5 had significant upregulation of cytokines (*Il34*, *Il4*, *Cxcl9*, *Ccl5*) and several DAMPs, including heat shock proteins, S100 proteins, and HMGB-1 relative to other cardiomyocytes (Figure S13). Several of these genes were also identified as eDEGs, suggesting their early emergence in disease and their role as potential disease drivers.

Our snRNA-seq further revealed increased immune cell populations in cKO versus cWT hearts at 25-dpi (Figure 4A), confirmed by CD45 (pan-leukocyte marker) immunolabeling (Figure 4B). Macrophage and monocyte populations (CD68^+^) showed a slight, nonsignificant increase at 11-dpi, which progressively increased and reached significance by 25-dpi (Figure 4C), confirmed by immunoblotting (Figure S10C) and consistent with the early emergence of innate immunity-related eDEGs (Figure 2D). The snRNA-seq data confirmed the expansion of existing immune cell populations, with increases in myeloid subpopulations Mye1 (22%–32%), Mye2 (24%–28%), and Mye4 (13%–23%), and natural killer/T-cell subpopulation NKT2 (29%–51%) (Figure 4A; Figure S14A). We classified Mye2 and Mye4 as tissue resident macrophages and Mye1 as infiltrating macrophages, based on marker expression^57^ (Figure 4D, Table S8). These data indicate that both immune cell recruitment and resident macrophage expansion shape the evolving inflammatory landscape. Supporting local cardiomyocyte-immune interaction, costaining for CD68 and the CM3/CM5 marker RTN4 revealed increased spatial proximity between CD68- and RTN4-positive regions in cKO versus cWT hearts (Figure 4E and 4F), supporting a model in which cytokine release promotes local immune recruitment/expansion, driving sterile inflammation.

**Figure 4.**
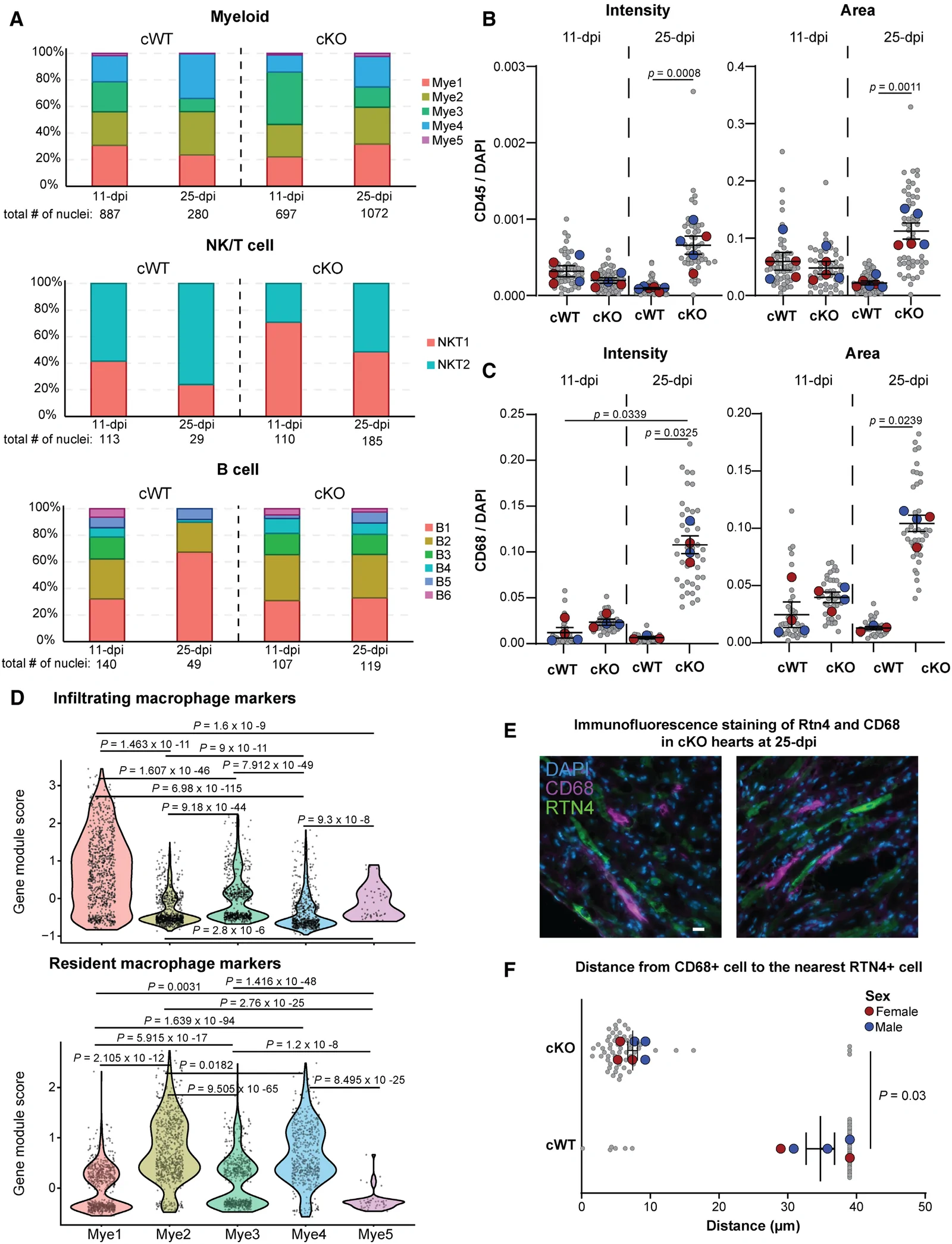
**Infiltration and expansion of innate immune cells in cKO hearts. A**, Number of nuclei captured in each genotype within individual immune cell subclusters and in total. Quantification of (**B**) CD45 and (**C**) CD68 immunofluorescence intensity normalized to 4′,6-diamidino-2-phenylindole (DAPI) intensity (**left**) and area coverage normalized to DAPI area coverage (**right**) for cKO and cWT cardiac tissue sections, collected at 11- and 25-days post-injection (dpi). N=5–6 mice/genotype for CD45 and N=4 mice/genotype for CD68. **D**, Gene module scores for infiltrating (**top**) and resident (**bottom**) gene markers across individual myeloid subpopulations in the integrated single-nucleus RNA sequencing (snRNA-seq) data set. Dots represent individual nuclei. **E**, Immunofluorescence images of cKO cardiac tissue sections at 25-dpi stained for CD68 (magenta), reticulon 4 (RTN4; green), and DAPI (blue). Scale bar, 20 µm. **F**, Nearest neighbor analysis showing distances (µm) between RTN4-positive and CD68-positive regions in cKO (**top**) and cWT (**bottom**) hearts. N=5–6 animals/genotype. For statistical comparison, we selected 40 µm as an arbitrary threshold for lack of spatial proximity. Statistical annotation: (**B**, **C**, and **F**) Colored circles represent average values for individual male (blue) or female (red) mice. Gray circles represent individual fields of view. Statistical tests: (**B** and **C**) Kruskal-Wallis test with Dunn post hoc test, (**D**) pair-wise Wilcoxon rank-sum test with Benjamini-Hochberg correction, and (**F**) Mann-Whitney *U* test. cKO indicates inducible cardiomyocyte-specific *Lmna* deletion mouse model; and cWT, controls lacking the floxed *Lmna* alleles.

Concurrently, we detected a small but significant increase in *Lmna* transcript levels in cKO hearts between 11- and 25-dpi (Figure S3B). To determine whether this change reflects infiltration/expansion of immune cells, which express lamin A in the cKO model, or loss of cardiomyocytes with low lamin A/C levels, we quantified cardiomyocyte numbers (PCM1-positive nuclei) and lamin A levels by immunofluorescence. Neither cardiomyocyte abundance (Figure S3C) nor lamin A levels (Figure S3D) significantly changed over time, suggesting that the modest increase in bulk *Lmna* expression likely reflects immune cell infiltration/expansion (Figure 4A through 4D).

### Lamin A/C-Depleted Cardiomyocytes Affect Noncardiomyocyte Gene Expression Through Altered Cell-Cell Communication

Our snRNA-seq analysis identified several noncardiomyocyte subpopulations with distinct gene expression changes in cKO compared with cWT hearts (Figure S14A through S14C; Tables S5 through S12), despite retaining lamin A/C expression. Fibroblasts showed progressive transcriptional changes in cKO versus cWT hearts, based on the fraction of associated DEGs at each time point (Figure 5A). In immune cells, in contrast, over 65% of the 25-dpi DEGs were already misregulated at 11-dpi (Figure 5A), indicating that immune cell transcriptional changes occur earlier in disease than those in fibroblasts.

**Figure 5.**
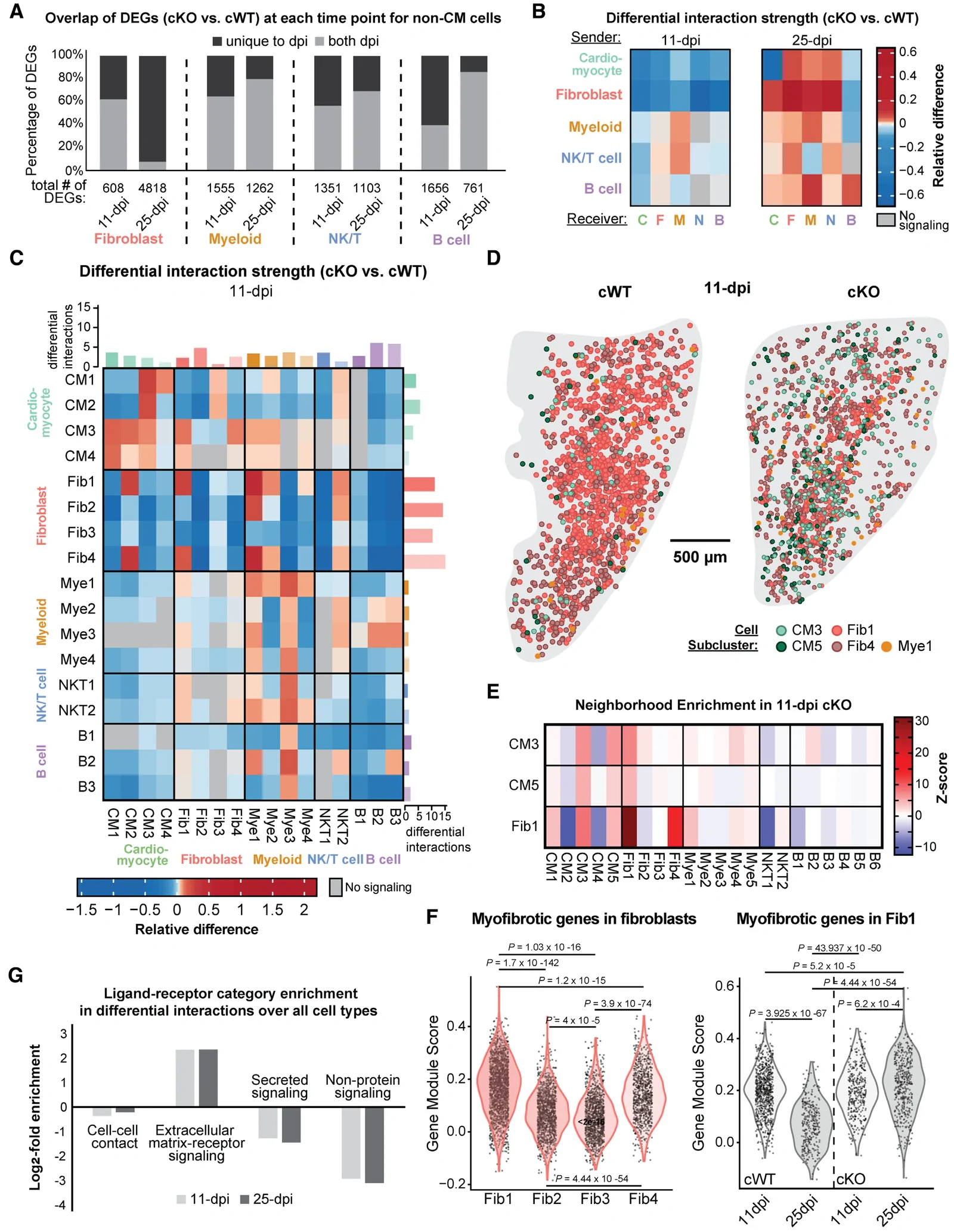
**Prediction of intercellular signaling in cKO cardiomyocytes, fibroblasts, and myeloid cells reveals a fibrosis-inflammation link driven by CM3/CM5. A**, Percentage of differentially expressed genes (DEGs) between cKO and cWT hearts at 11- and 25-days post-injection (dpi), for individual noncardiomyocyte cell types. DEGs unique to (light gray) or shared between (dark gray) early and late time points are denoted. The total DEG counts are indicated under each column. **B** and **C**, Heat map showing the difference (cKO vs cWT) in the interaction strength of sender (rows) and receiver (columns, initial letter) cell types (**B**) and subpopulations (**C**). The numbers of differential interactions for each row and column are indicated in the bar plot. Subpopulations with insufficient nuclei for analysis were removed (ie, CM5, Mye5, and B4–B6). **D**, Spatial transcriptomics annotation of cardiomyocyte (CM) (CM3, light green; CM5, dark green), fibroblast (Fib1, pink; Fib4, maroon), and myeloid (Mye1, orange) subpopulations in cWT and cKO 11-dpi ventricles (tissue boundaries shown in gray). **E**, Heat map of *Z*-score neighborhood enrichment for subpopulations in 11-dpi cKO ventricles based on spatial transcriptomics. **F**, Gene module scores for a myofibrotic gene set in fibroblast subpopulations (**left**) and split by experimental condition for Fib1 (**right**, light gray—cWT, dark gray—cKO). Dots represent individual nuclei. **G**, Log_2_-fold enrichment of particular ligand-receptor categories associated with differential interactions over all cell types at 11-dpi (light gray) and 25-dpi (dark gray). Statistical tests: (**F**) pair-wise Wilcoxon rank-sum test with Benjamini-Hochberg correction. cKO indicates inducible cardiomyocyte-specific *Lmna* deletion mouse model; and cWT, controls lacking the floxed *Lmna* alleles.

Transcriptomic changes in noncardiomyocytes could result from signaling by lamin A/C-depleted cardiomyocytes, for example, release of cytokines and DAMPs that activate immune cells^58^ and fibroblasts,^59,60^ or in response to cardiac dysfunction. To distinguish between these mechanisms, we implemented CellChat^26^ to compare predicted cell-cell communication between cKO and cWT hearts. Overall, cell-cell communication among all cell types was reduced in cKO versus cWT hearts at 11-dpi but increased by 25-dpi (Figure 5B; Figure S15). Both outgoing (sender) and incoming (receiver) communication for fibroblasts showed a marked shift from weaker cell-cell interaction strength at 11-dpi to stronger interaction strength at 25-dpi in cKO relative to cWT. Fibroblast outgoing communication at 25-dpi represented the strongest relative increase in communication received by innate immune cells (myeloid and NK/T cells) in cKO hearts compared with cWT. Furthermore, myeloid and NK/T outgoing cell-cell communication interactions were also stronger in cKO than cWT at 25-dpi, further supporting a role for innate immunity in disease progression. In contrast, B cells showed weaker incoming communication from other cell types at both time points. Endothelial and mural cells similarly showed reduced communication with cKO cardiomyocytes at 11- and 25-dpi (Figure S15), suggesting limited contribution to pathogenic signaling.

Although overall communication increased in cKO compared with cWT hearts at 25-dpi, cardiomyocyte-to-cardiomyocyte communication strength was markedly reduced at both time points (Figure 5B). CellChat analysis at 11-dpi revealed that this reduction was primarily driven by CM1 and CM2 (Figure 5C; Figure S16). In contrast, CM3 exhibited increased communication with other cardiomyocytes, myeloid subpopulations Mye1 and Mye2, and fibroblast subpopulations Fib1 and Fib4 (Figure 5C). While CM5 contained too few nuclei in the 11-dpi cWT sample to assess differential communication, CellChat predicted interactions between CM5 and Fib1, Mye1, and Mye2 in cKO hearts, similar to CM3 (Figure S16). In addition, Fib1 and Fib4 showed increased interactions with Mye1 and Mye2 in cKO hearts. Spatial transcriptomics revealed increased proximity among CM3, CM5, Fib1, Fib4, and Mye1 in cKO versus cWT hearts (Figure 5D and 5E; Figure S17), with Fib1 and Fib4 showing pronounced shifts in spatial distribution at 11-dpi (Figure 5D), further supporting increased communication between CM3/CM5 cardiomyocytes, monocytes/macrophages, and specific fibroblast subpopulations.

Fib1 exhibited a transcriptional profile consistent with myofibroblasts (Figure 5F, left), which significantly intensified over time in cKO (Figure 5F, right).^61^ Pathogenic fibroblast activation was further supported by CellChat prediction that cardiomyocyte and noncardiomyocyte interactions were largely mediated by extracellular matrix (ECM)-receptor pathways (Figure 5G). These results align with recent work in another cKO mouse model showing increased ECM-mediated cardiomyocyte-fibroblast signaling^38^ and suggest that fibrosis is not merely a secondary consequence of cardiac dysfunction but a direct contributor to *LMNA*-DCM pathogenesis.

Together, enhanced cell-cell communication (Figure 5C) and spatial proximity (Figure 5D and 5E) between CM3, CM5, Fib1, Fib4, and Mye1 in cKO versus cWT hearts at 11-dpi suggest a pathogenic communication network between disease-specific cardiomyocytes (CM3/CM5), myofibrotic fibroblasts (Fib1), and infiltrating macrophages (Mye1). These data indicate progressive reorganization of cardiomyocyte and noncardiomyocyte interactions starting early in disease, driving the inflammatory and fibrotic remodeling characteristic of *LMNA*-DCM.

### Reducing NE Damage in Lamin A/C-Deficient Cardiomyocytes Partially Restores Gene Expression and Substantially Ameliorates *LMNA*-DCM Pathology

Our data suggest that NE rupture in a subset of cKO cardiomyocytes drives altered gene expression, sterile inflammation, fibrosis, and cardiac dysfunction. We therefore hypothesized that reducing NE rupture could reverse many transcriptional changes—even in the continued absence of lamin A/C—and ameliorate disease progression. To test this hypothesis, we generated mice with inducible, cardiomyocyte-specific lamin A/C depletion and concurrent expression of a dominant negative nesprin construct consisting of the DN KASH^15^ (Figure 6A). DN KASH displaces endogenous nesprins from the NE, leading to LINC complex disruption and reduced cytoskeletal forces on the nucleus.^5,6,13,14^ Accordingly, tamoxifen treatment significantly reduced the fraction of cKO cardiomyocytes with NE rupture (BAF-positive nuclei) compared with cKO littermate controls lacking the DN KASH transgene (Figure 6B; Figure S18). Notably, cardiomyocyte-specific LINC complex disruption dramatically improved cardiac function in cKO mice (Figure 6C; Figure S19). Although cardiac function was not completely rescued, DN KASH expression extended survival of cKO mice from ≈30 dpi to over 400-dpi, which was the experimental end point. cWT mice expressing DN KASH had normal survival (Figure 6D).

**Figure 6.**
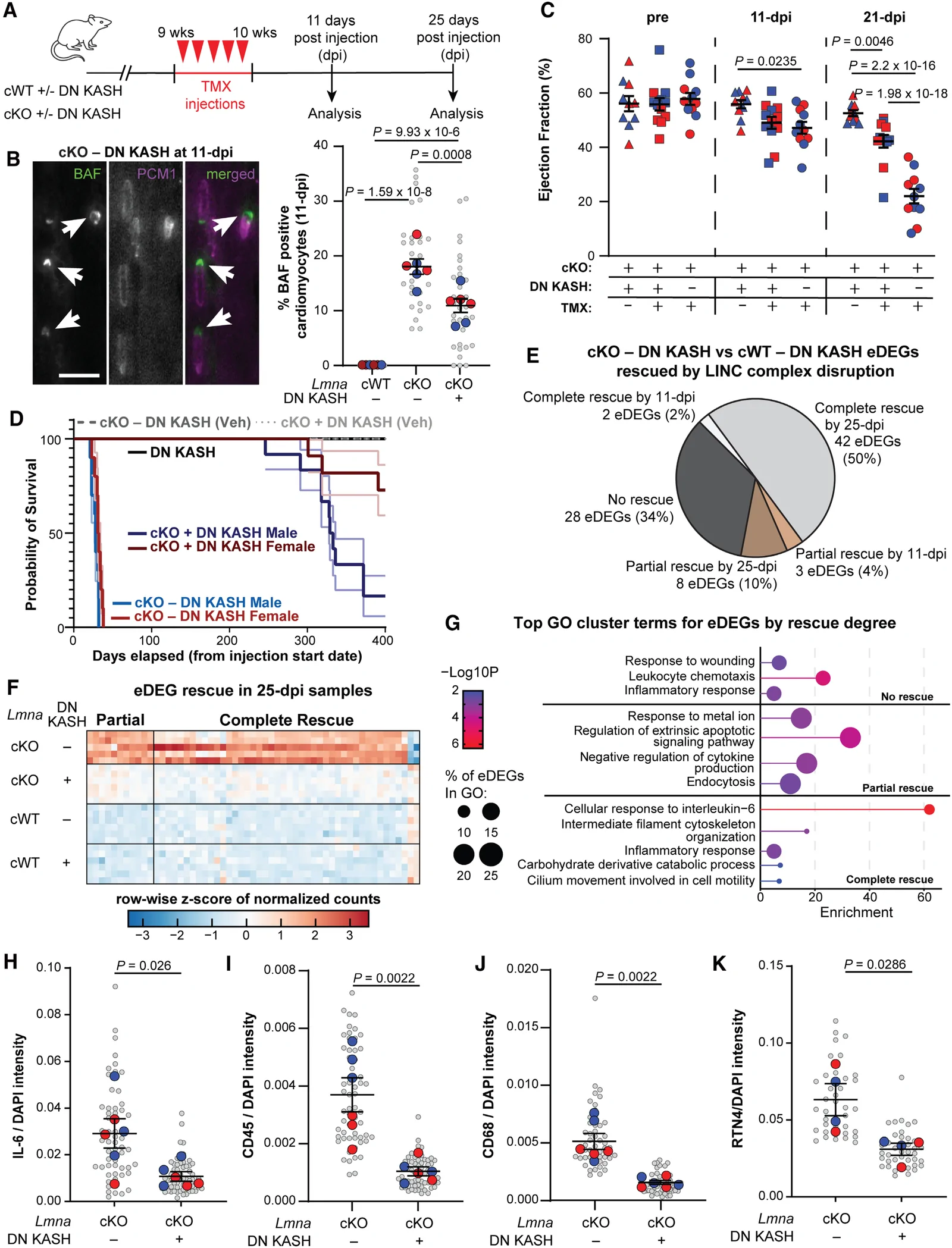
**Linker of nucleoskeleton and cytoskeleton (LINC) complex disruption improves cardiac function and survival in cKO mice by reducing nuclear envelope (NE) rupture and transcriptional activation of cytosolic DNA sensing-mediated inflammation. A**, Experimental timeline for concurrent induction of *Lmna* deletion and LINC complex disruption by cardiomyocyte-specific dominant-negative KASH domain protein (DN KASH) expression. Echocardiography and tissue collection were performed at the indicated time points. **B**, Representative images of barrier-to-autointegration factor (BAF)-positive (green) cardiomyocytes pericentriolar material 1 (PCM1)-positive (magenta) in cKO mice without LINC complex disruption (**left**) and quantification for cWT and cKO mice with (DN KASH +) and without (DN KASH −) LINC complex disruption (**right**). Arrows indicate representative cardiomyocytes. N=5–6 animals/genotype. **C**, Left ventricular ejection fraction (EF) measured before tamoxifen (TMX) injection (pre), or at 11- and 25-days post-injection (dpi). Symbols represent %EF of individual male (blue) or female (red) mice. N=10–12 animals/genotype. Error bars represent mean ± SEM. **D**, Kaplan-Meier survival plot for cWT mice with LINC complex disruption (cWT + DN KASH, N=5 males and 5 females), cKO mice treated with vehicle (cKO vehicle, N=5 males and 5 females), cKO mice with DN KASH treated with vehicle (cKO + DN KASH vehicle, N=5 males and 5 females), cKO mice with LINC complex disruption (cKO + DN KASH, N=23 mice: 12 males and 11 females), and cKO mice (cKO, N=20 mice: 10 males and 10 females). **E**, Fraction of emerging differentially expressed genes (eDEGs) with complete (44 eDEGs, light gray), partial (11 eDEGs, brown), or no rescue (28 eDEGs, dark gray) following LINC complex disruption. **F**, Heat map of row-wise *Z*-score-normalized read counts for eDEGs rescued by LINC complex disruption, grouped by degree of rescue (complete, **right**; partial, **left**) and sample condition. **G**, Gene ontology (GO) cluster terms enriched in eDEGs stratified by rescue category (none, **top**; partial, **middle**; complete, **bottom**). Dot color indicates enrichment significance; size reflects proportion of eDEGs per term. **H** through **K**, Quantification of interleukin 6 (IL6) (**H**), CD45 (**I**), CD68 (**J**), and reticulon 4 (RTN4) (**K**) intensity normalized to 4′,6-diamidino-2-phenylindole (DAPI) in cKO with (DN KASH +) and without (DN KASH −) LINC complex disruption. Colored circles represent average values for individual male (blue) or female (red) mice. Gray circles represent individual fields of view. N=6 animals/genotype for IL-6, CD45, and CD68. N=4 animals/genotype for RTN4. Statistical tests: (**B**) 1-way ANOVA test with Tukey post hoc, (**C**) 2-way repeated measures ANOVA with Tukey post hoc test, and (**H** through **K**) Mann-Whitney *U* test. cKO indicates inducible cardiomyocyte-specific *Lmna* deletion mouse model; and cWT, controls lacking the floxed *Lmna* alleles.

To further assess the effects of LINC complex disruption, we performed bulk RNA-seq on left ventricles from cKO and cWT mice, with and without concurrent DN KASH expression. We first applied our RNA-seq analysis to cKO and cWT littermates without DN KASH expression. We observed a progressive increase in DEGs from 11-dpi (n=208) to 25-dpi (n=3426; Figure S20A), mirroring our original cKO versus cWT analysis (Figure 2B and 2C; Figure S2A), and identified 83 eDEGs (Figure S20A through S20C). The eDEGs were enriched for GO terms related to inflammation, apoptosis, and innate immunity (Figure S20D), consistent with our findings in the original cohort (Figure 2).

We next evaluated the effect of LINC complex disruption on gene expression. In cWT hearts, DN KASH expression caused only minimal transcriptional changes (4 DEGs detected by 25-dpi; Table S13), indicating that cardiomyocyte-specific LINC complex disruption in adult mice is well tolerated.^15,62^ In the cKO hearts, DN KASH expression restored expression of most eDEGs (n=44, 53%) and DEGs (n=1786, 52%) to levels statistically indistinguishable from cWT controls (Figure 6E and 6F; Figure S21; Table S14); we designated these genes as complete rescue. An additional subset showed partial rescue, including 11 eDEGs (13%) and 226 DEGs (7%), which differed significantly between cKO samples with and without LINC complex disruption but did not return to cWT levels (Figure 6E and 6F; Figure S21; Table S14). Several eDEGs and DEGs were rescued already at 11-dpi, indicating their potential role as disease drivers; the majority were rescued by 25-dpi (Figure 6E; Figure S21; Table S14).

GO term analysis revealed that the partially and completely rescued eDEGs were significantly enriched for apoptosis- and inflammation-related terms, whereas terms such as inflammatory response and leukocyte chemotaxis persisted among the no-rescue group (Figure 6G). These findings likely reflect multiple contributions, including the residual NE rupture in some cKO cardiomyocytes despite DN KASH expression (Figure 6B), the multifactorial nature of inflammation, and transcriptional changes intrinsic to lamin A/C depletion, independent of NE rupture. Nonetheless, LINC complex disruption substantially mitigated expression of key candidate pathological drivers in cKO hearts, including rescue of genes associated with cytosolic DNA sensing (*Rtn4*, *Ifi204*, *Tlr9*, *Il6*, *Irf7*, and *Hspa1a*; Figure S22), infiltrating macrophages (47% reduction; Figure S23, left), and myofibroblasts (65% reduction; Figure S23, right), and significant reduction of CD45, CD68, IL-6 (interleukin-6), and RNT4 protein levels (Figure 6H through 6K).

Consistent with a pathogenic role for inflammation, immune cell burden (CD45^+^ and CD68^+^ cells) inversely correlated with ejection fraction across all cKO conditions (Figure S24). Furthermore, cellular response to interleukin-6 was the most strongly enriched GO term among the completely rescued genes following DN KASH expression (Figure 6G, bottom), while negative regulation of cytokine production and endocytosis were significantly enriched in the partially rescued genes (Figure 6G). Together, these findings indicate that LINC complex disruption confers therapeutic benefit by reducing NE rupture and downstream inflammatory signaling, thereby reducing immune cell recruitment/expansion and pathogenic cardiac remodeling.

## Discussion

Using temporally resolved, multilevel transcriptomic analyses, we identified 2 disease-specific cardiomyocyte subpopulations (CM3 and CM5) that were largely responsible for altered gene expression in cKO hearts, despite representing only a small fraction of cardiomyocytes in cKO hearts (CM3: 10.12%; CM5: 9.88%). This fraction aligns with the percentage of cKO cardiomyocytes undergoing NE rupture observed here (18%; Figure 6B) and in other studies.^6,38^ CM4 lies between CM3 and CM5 in the transcriptional space and shares eDEG signatures with CM3 and CM5 at 25-dpi (Figure 2I). Pseudotime analysis (Figure S25A) and the shared enrichment of genes associated with oxidative phosphorylation in CM4 and CM5 (Figure S25B) indicate that CM5 likely emerged from CM4. This is reflected by a decline in CM4 cells as CM5 expands in cKO hearts (Figure 2G), and by CM4 cells at the CM4-CM5 interface exhibiting CM5-like transcriptional profiles (Figure S25C). In cWT hearts, CM4 increases with age (Figure 2G), consistent with cardiomyocyte maturation and metabolic shift from glycolysis^63^ (Figure S25B). Thus, CM4 plays a dual role: contributing to disease-associated progression in cKO hearts, while representing a maturation state in cWT hearts.

We found that LINC complex disruption reduces NE rupture in lamin A/C-depleted cardiomyocytes (Figure 6B), resulting in near-complete rescue of cardiac function and survival in cKO mice (Figure 6C and 6D), as well as normalization of over 50% of misregulated genes, despite the continued depletion of lamin A/C. These findings provide strong evidence mechanistically linking NE rupture to altered gene expression in *LMNA*-DCM and support NE rupture as an early catalyst of disease pathology. Approximately 66% of eDEGs (Figure 6E) were rescued by LINC complex disruption, suggesting that the gene regulation and the structural^3,4^ hypotheses are linked. The broad effects of NE rupture may also explain why targeting specific downstream pathways, such as p53 or STING, had only limited success in preclinical models.^64–66^

Of note, DN KASH competes with endogenous KASH-domain-containing nesprins and is thus unlikely to achieve complete LINC complex disruption. This likely explains the residual NE rupture (10% of nuclei; Figure 6B) and incomplete transcriptional rescue (Figure 6E) in cKO hearts. The nonrescued transcriptional changes may also reflect epigenomic alterations caused by loss of lamin A/C, including changes in lamina-associated domains (LADs) and at DNA methylation sites,^67,68^ which were not assessed here. Nevertheless, the reduced inflammation and large-scale normalization of transcriptomic changes achieved by LINC complex disruption were sufficient to significantly improve cardiac function and extend survival by >1 year, supporting the interpretation that reducing NE rupture presents a central rescue mechanism. Nonetheless, we cannot exclude that LINC complex disruption-mediated rescue includes additional mechanisms, including alterations in chromatin organization,^69^ epigenetic modifications,^70^ and cytoskeleton organization.^6^

Our study revealed a previously unrecognized role of cytosolic PRR signaling and activation of innate immunity in cKO hearts (Figure 2D and Figure 4B through 4D). As most cardiomyocytes lack cGAS/STING, we propose that innate immune activation following NE rupture is mediated by other PRR components,^71,72^ such as TLRs and MyD88, which were enriched among eDEGs and in CM3 and CM5 (Figures 2D, 3F through 3I, 4E and 4F; Figure S11). Cross-referencing our data with human clinical data, we detected PRR pathway activation in both *LMNA*-DCM and nonlaminopathy DCM patient samples^30^ (Figure S12). While human data reflect end-stage disease, the temporal resolution of our cKO model supports PRR signaling as a potential disease driving mechanism. DNA and DAMPs released by damaged cardiomyocytes may further activate PRR signaling in fibroblasts and immune cells, consistent with our integrated single-nucleus and spatial transcriptomic evidence of pathogenic cell-cell communication.

Our computational approach robustly identified disease-driving transcriptional programs. However, we recognize that the inherent subtlety of early transcriptional changes makes them susceptible to biological noise, such as stochastic cell-type abundance (eg, cardiomyocytes with NE rupture or infiltrating immune cells), cohort-specific variability (eg, transgene insertion), and sequencing platform differences. This sensitivity is reflected in the limited concordance in log_2_-fold-change directionality between early (11-dpi) and late (25-dpi) genes in the DN KASH rescue experiments (Figure S20A and S20C), largely driven by transcriptional variability in 11-dpi cKO (Figure S26A and S26B), which led to fewer eDEGs being identified (Figures S2A and S20A). Nonetheless, DN KASH expression normalized even discordant genes (Figure S20B and S20E), and late-stage transcriptional profiles converged strongly across data sets (Figure S26B and S26C). Importantly, despite variation in the expression of individual genes at the early time point, the identified biological pathways were highly consistent across experiments, underscoring the value of prioritizing pathway-level analysis over individual early genes in isolation. Ultimately, our eDEG framework captured early transcriptional differences that matured into robust disease signatures and revealed key pathways rescued by LINC complex disruption, supporting their biological relevance and predictive power.

### Limitations of the Current Study

Although the cKO model enables identification of early *LMNA*-DCM disease drivers, several caveats merit consideration. Human *LMNA*-DCM arises from germline mutations,^73^ which could affect cardiac development and noncardiomyocyte cell functions, further contributing to disease progression, which is not reflected in our cardiomyocyte-specific lamin A/C-depletion model. Other affected tissues might also contribute to severity. Consistent with this, LINC complex disruption almost completely rescues cKO mice but only partially rescues global *LMNA* mutant models.^74^ In addition, the sudden depletion of lamin A/C in the cKO model may cause a more rapid disease progression by circumventing compensatory adaptations during development; while less physiological, this feature of the cKO model might actually aid in the identification of pathogenic mechanisms.

Our analysis focused on eDEGs and associated pathways, excluding late-stage DEGs not significantly altered early in disease or showing inconsistent directionality. While such late-stage DEGs may primarily reflect downstream consequences of disease progression, rather than disease-driving mechanisms, they may nonetheless contribute to cardiac dysfunction.

Finally, transcriptomic changes do not always reflect protein levels, posttranslational modifications, or changes in localization of proteins. Although several key findings were confirmed at the protein level, further studies are needed to fully validate the proposed mechanisms.

## Conclusions

We propose a model (Figure S27) in which NE rupture in a subset of cardiomyocytes activates cytosolic PRR pathways, independent of cGAS/STING, driving transcriptional and functional changes that initiate pathogenic cell-cell communication involving monocytes/macrophages and fibroblasts, leading to inflammation, fibrosis, and cardiac remodeling and ultimately causing systolic dysfunction and sudden cardiac death. Release of DAMPs and PRR signaling by immune cells and fibroblasts might further amplify inflammation and disease progression. The strong rescue conferred by DN KASH expression highlights the therapeutic potential of targeting the LINC complex or other strategies to reduce NE rupture in *LMNA*-DCM. In addition, our findings suggest that targeting aberrantly activated pathways triggered by NE rupture or restoring perturbed cell-cell communication may serve as alternative or complementary therapeutic strategies for *LMNA*-DCM.

## Article Information

### Acknowledgments

The authors thank Stephen Weiss (University of Michigan) and Didier Hodzic (Washington University at St. Louis) for generously providing the floxed *Lmna* mice and dominant-negative KASH domain protein (DN KASH) expressing mice, respectively. Bulk RNA sequencing was performed by the Biotechnology Resource Center Genomics Facility (RRID:SCR_021727) at the Cornell Institute of Biotechnology. Statistical consulting was provided by Kenneth Tyler Wilcox through the Cornell Statistical Consulting Unit.

### Author Contributions

N. Zuela-Sopilniak, J.L.P. Morival, and J. Lammerding conceived and designed the study. N. Zuela-Sopilniak, J.L.P. Morival, and I. Ntekas performed the experiments and acquired the data. N. Zuela-Sopilniak, J.L.P. Morival, I. Ntekas, M.A. Elpers, R. Agarwal, S.J. Henretta, and J.D. Odell analyzed and interpreted the data. N. Zuela-Sopilniak, J.L.P. Morival, and J. Lammerding wrote the manuscript. J. Lammerding and I. De Vlaminck secured funding. All authors provided critical revision of the manuscript for important intellectual content and approved the final version to be published.

### Disclosures

None.

### Supplemental Material

Expanded Materials & Methods

Tables S1–S17

Figures S1–S27

Major Resources Table

References 75–92

## Supplementary Material


