# Supplementary material for: Reducing Nuclear Rupture Rescues Gene Expression and Survival in LMNA-DCM
Source: Circ Res. 2026 Sep 25;139(10):e327744. doi: 10.1161/CIRCRESAHA.125.327744 (PMC13682672; doi:10.1161/CIRCRESAHA.125.327744)
Supplement: Supplementary file 1 [file res-139-e327744-s001.pdf]

Supplemental Materials

The Supplemental Materials in this file contain Supplemental Methods, Supplemental Figures S1-S27, and Supplemental Table S1.

Supplemental Tables S2-S17 are provided as separate files.

Expanded Materials & Methods

Animals. Mice expressing a transgene containing the mouse cardiac-specific α-myosin heavy chain promoter (αMHC or Myh6) directing expression of a tamoxifen-inducible Cre recombinase (MerCreMer) in juvenile and adult cardiac myocytes (αMHC-MerCreMer) and mice possessing loxP sites flanking exon 2 of the lamin A gene (*Lmna* floxed or *Lmna*$^{fl/fl}$)[75], were obtained from The Jackson Laboratory (JAX) in a C57BL/6 background (αMHC-MerCreMer: B6.FVB(129)-A1cf$^{Tg(Myh6\text{-}cre/Esr1*)1Jmk}$/J, strain 005657; and *Lmna* floxed: *Lmna*$^{tm1.1Yxz}$/J, strain 026284, respectively) and genotyped as instructed. Mice expressing a recombinant KASH domain (DN KASH) were generated by the Hodzic lab[15]. All mice used in this study were maintained in the same C57BL/6 genetic background and backcrossed for at least three generations. To generate the inducible, cardiac specific *Lmna* deletion mouse model (cKO), αMHC-MerCreMer and *Lmna* floxed mice were crossed to obtain mice hemizygous for αMHC-MerCreMer and homozygous for *Lmna* floxed (*Lmna*$^{fl/fl}$). αMHC-MerCreMer hemizygous expressing wild-type *Lmna* (*Lmna*$^{+/+}$) littermates served as controls (cWT). To generate the inducible, cardiac specific *Lmna* deletion and LINC complex disruption mouse model (cKO + DN KASH), αMHC-MerCreMer, DN KASH, and *Lmna* floxed mice were crossed to obtain mice hemizygous for αMHC-MerCreMer, hemizygous for DN KASH, and homozygous for *Lmna* floxed (*Lmna*$^{fl/fl}$). Littermates hemizygous for αMHC-MerCreMer and homozygous for *Lmna* floxed (*Lmna*$^{fl/fl}$), and mice hemizygous for αMHC-MerCreMer and expressing wild-type *Lmna* (*Lmna*$^{+/+}$) without DN KASH expression served as controls (cWT – DN KASH). To induce lamin A/C depletion and/or LINC complex disruption in cardiomyocytes, tamoxifen (Cayman chemicals catalog#13258) was dissolved in sunflower seed oil (Sigma catalog# 88921) to a final concentration of 30 mg/kg (3 µl/g) and injected intraperitoneally at 9-10 weeks of age for five consecutive days, followed by a one-week-long washout period. Vehicle-only injections served as additional controls. For tissue collection, mice were anesthetized at the appropriate timepoints using Isoflurane (Covetrus catalog# 11695-6777-2). Immediately following cervical dislocation, hearts were harvested, washed with ice cold 1X Phosphate Buffer Saline (PBS), and processed for downstream analysis. All experiments, including breeding, maintenance, and euthanasia of animals, were performed in accordance with relevant guidelines and ethical regulations approved by the Cornell University Institutional Animal Care and Use Committee (IACUC protocol 2011–0099). Genotyping was performed by PCR using genomic DNA isolated from tail clippings following the protocol and sequencing primers suggested by JAX. Mice were housed in a disease-free barrier facility with 12/12 h light/dark cycles.

Cardiac function. Mice were anesthetized with 2% isoflurane and placed on a stereotactic heated scanning base (37°C) attached to an electrocardiographic monitor. Left ventricular function was determined with a Vevo 2100 imaging system (VisualSonics) equipped with a MS550D transducer (22–55 MHz). Ejection fraction (EF%), fraction shortening (FS%), left ventricle diameter during systole (mm) and volume during systole (mm) were measured for at least five cardiac cycles using AM mode images. The average heart rate for all studied animals was 461 ± 24 beats per minute (BPM). Analysis was performed using the AutoLV Analysis Software (VisualSonics) and conducted by observers blinded to the mouse genotype and/or treatment groups. As multiple

measurements were taken from a single mouse at different time points, the statistical test used to assess genotype- and time point-dependent changes in cardiac function were adjusted for repeated measurement and are specified in the appropriate figure legends.

Immunofluorescence and histology of mouse tissues. Immediately following tissue collection, hearts were washed with 1X PBS and fixed in 4% paraformaldehyde (diluted in 1X PBS) at 4°C overnight. Samples were stored for up to one week in 70% ethanol (diluted in 1X PBS) at 4°C before being embedded in paraffin, sectioned, and stained with Masson's Trichrome using standard methods at the Animal Health Diagnostic Center (College of Veterinary Medicine, Cornell University). For immunofluorescence analysis, samples were washed with PBS and flash frozen in Tissue-Tek® O.C.T. Compound (Sakura, #4583) in an ethanol bath, followed by storage at –70°C. Frozen tissue blocks were cryosectioned using an Epredia™ Microm HM525 NX Cryostat to a thickness of 5 µm, mounted on 25 × 75 × 1.0 mm Superfrost Plus Microscope slides (Fisherbrand® #12-55015) and left to air dry for 1 hour. Samples were stored at –20°C until processed for immunofluorescence staining and imaging. Prior to staining, slides containing tissue were equilibrated to room temperature and rehydrated with 1X PBS for 5 minutes. When using primary antibodies raised in mice, tissues were initially blocked with M.O.M® (Mouse on Mouse) Blocking Reagent (Vector laboratories, Cat# MKB-2213-1, diluted 1:20 in 1X PBS) for 1 hour at room temperature, followed by additional blocking and permeabilization with a solution of 3% BSA, 5% filtered horse serum in PBS-T (0.05% Triton-X 100 and 0.03% Tween (Sigma) in 1X PBS) for 1 hour at room temperature. Tissues were then incubated with primary antibodies diluted in blocking solution at 4˚C overnight. The following primary antibodies were used for this study: pcm1, Santa Cruz sc398365, 1:50 dilution; pcm1, Sigma HPA023370, 1:500 dilution; Lamin A/C (E-1), Santa Cruz sc376248, 1:100 dilution; lamin A, Sigma MAB3540, 1:100 dilution; CD68 (E3O7V), Cell Signaling 97778T, 1:400 dilution; BAF, Santa Cruz sc-166324, 1:250 dilution; Rtn4, Novus NB100-56681SS, 1:200 dilution; IL-6, Novus NB600-1131, 1:100; CD45 (D3F8Q), Cell Signaling 70257S, 1:100 dilution. Samples were washed with PBS-T and incubated for 1 hour at room temperature with AlexaFluor secondary antibodies (Invitrogen, 1:300 dilution) and Hoechst Nucleic Acid Stains (ThermoFisher 62249, 1:1000) in PBS-T. Cardiac sections were processed, stained, imaged, and analyzed in parallel under identical experimental and acquisition conditions. All markers showed consistent and reproducible signal intensity, with low fluorescence background. Representative images were selected based on image quality and clarity to illustrate the observed experimental effects and do not necessarily correspond to the mean or average quantitative value. All quantitative analyses were performed using the full dataset.

Proximity Image analysis. Images were processed using a custom CellProfiler[16] (v4.2.8) script to identify regions of interest (ROIs) corresponding to DAPI-positive nuclei, Rtn4-positive cells, and CD68-positive cells. DAPI-positive nuclei were identified using an Otsu two-class threshold to raw DAPI-channel images, followed by smoothing and segmentation based on size and shape. Rtn4-positive cells were detected using a three-class Otsu threshold on raw Rtn4-channel images, smoothed, and identified based on size. CD68-positive cells were identified by applying manually set threshold bounds, consistent across all images, to raw CD68-channel images, followed by size-based

identification. The distance between CD68-positive ROIs and the nearest Rtn4-positive ROI was calculated using the MeasureObjectNeighbors module. All image data was exported from CellProfiler for post-processing using the ExportToSpreadsheet module.

Western Blot analysis. Cardiac tissue was placed in homogenization strips (VWR, Cat #TN0946-08RS) in high salt RIPA buffer [12 mM sodium deoxycholate, 50 mM Tris-HCl pH 8.0, 750 mM NaCl, 1% (v/v) NP-40 alternative and 0.1% (v/v) SDS in ultrapure water] and a small metal ball (McMaster-Carr, Cat #1598K23). Tissues were homogenized for three 3-minute cycles of 30 shakes/sec using a TissueLyser II (Qiagen). Homogenized samples were sonicated (Branson 450 Digital Sonifier) for 30 s at 36% amplitude, boiled for 2 min, centrifuged at 4°C for 10 min at 14,000 g and stored at −75°C. Protein concentrations were determined by BCA assay, and 10 µg of total protein from each heart was denatured in 5X Laemmli buffer by boiling for 3 min. Protein was loaded onto 4–12% Bis-Tris gels (Invitrogen NP0322), separated for 1.5 h at 100 V, then transferred for 1 h at 16 V onto PVDF membrane. Coomassie staining of the gel following transfer was used to assess equal protein loading. Membranes were blocked for 1 h in blocking buffer containing 3% BSA in Tris-buffered saline plus 1% Tween 20. Primary antibodies were added individually overnight at 4°C diluted in blocking buffer. Primary antibodies used: rabbit anti-CD68 (Cell Signaling 97778; 1:1000) and rabbit anti-Rtn4 (Novus Biologicals 100-56681; 1:1000). Membranes were washed 3 × 10 min, and secondary antibodies were added for 1 h at room temperature in blocking buffer, followed by 3 × 10 min washed. Secondary antibodies used: Licor IRDye 680RD donkey anti-mouse-IgG (LICORbio 926-68072; 1:5000) and Licor IRDye 800CW Donkey anti-Rabbit IgG (LICORbio 926-32213; 1:5000). Membranes were imaged using Odyssey Licor scanner and then cropped and brightness and contrast was adjusted using Image Studio Lite (version 5.2) software.

Bulk RNA sequencing (RNA-seq) processing. Left ventricle free wall tissues (excluding the atria and septum) were harvested at the appropriate timepoints, flash frozen in liquid nitrogen, and stored at –70°C until RNA was extracted. Frozen tissue was placed in an Eppendorf tube containing one metal bead and 500 µl of TRIzol® reagent (Invitrogen, 15596018). Tissues were homogenized for five 3-minute cycles of 30 shakes/sec using a TissueLyser II (Qiagen) and transferred to an Rnase free microfuge tube (Ambion, Cat #AM12400). 1:5 chloroform was added and mixed by shaking, followed by a 5-minute centrifugation at 4°C 14,000 rpm. The clear supernatant was carefully transferred to a gDNA eliminator spin column (Rneasy Plus Mini Kit, Qiagen, #74136) and RNA purification was performed according to manufacturer instruction. RNA quality control was performed by the Cornell Genomics core facility with a fragment analyzer (Agilent) to assess RNA integrity (RIN); RNA concentrations were quantified by Nanodrop (ThermoFisher Scientific). To generate cDNA directional RNA-seq libraries (New England Biolabs, #E7760), 1 µg of total RNA sample (with RIN ≥ 7) was enriched for mRNA (New England Biolabs, #E7490) for each condition. Each sample received a unique barcode for downstream multiplexing. cDNA library size distribution and concentrations were again assessed by fragment analyzer and Qubit. Final libraries were pooled in equimolar concentrations and sequenced. The 24 cKO and cWT samples at both time points (GSE269451), used to generate data for Figure 2, were pooled 12 per lane and sequenced on a NextSeq 500 (Illumina) with a 75 bp single-end kit by the Cornell

Genomics core facility. Libraries generated for the 48 samples (GSE304346) associated with the LINC complex disruption experiment (Figure 6) were pooled into a single lane and sequenced on a NovaSeq 6000 (Illumina) with an S4 flow cell and a 150 bp paired-end kit by Novogene.

Bulk RNA-seq analysis. We used STAR software[17] (version 2.7.10a) to generate a genome for read alignment using the GRCm38/mm10 (Ensembl release 102) mouse FASTA and GTF files. Fastq files for the DN KASH experiment (GSE304346) had TruSeq adaptors trimmed using fastp[76] (versions 0.20.0). All fastq files were aligned to the STAR genome using default parameter settings, resulting in a uniquely mapped read alignment ≥ 79%. Gene-level quantification was performed using the featureCounts function from Subread[18] (version 2.0.1), with parameters -s 2 (for directional RNA-seq libraries), -p (for paired end only with GSE304346), and the GENCODE GTF annotation file (vM23), to generate a count matrix using all samples. PCA and distance correlation matrix analyses were performed to identify and remove outliers (GSE269451: samples 3 and 19, GSE304346: samples 16 and 43) before downstream processing. Differential gene expression (DEG) analysis was performed in R (v4.1.1) by applying DESeq2[19] (v1.34.0) to the input count matrix. The design formula "~genotype*timepoint", where genotypes were either cKO, cWT (for GSE269451) or cKO – DN KASH, cWT – DN KASH, cKO + DN KASH, cWT + DN KASH (for GSE304346), and timepoints were either 11- or 25-days post-injection (dpi), was used. The ashr[77] shrinkage estimator was applied to all DEG results. DEGs were defined as genes with $|\log_2$-fold change$| \geq 1$, baseMean ≥ 10, and *p*-adj ≤ 0.05.
While larger sample sizes improve power in RNA-seq studies, our sample size (n = 6 per condition) is consistent with common practice in the field. Empirical benchmarking studies[78] indicate that decreasing sample number primarily reduces recall (i.e., power to detect differentially expressed genes), with comparatively smaller effects on precision, particularly when appropriate statistical frameworks are used. In our analysis, differential expression was assessed using DESeq2, which employs shrinkage-based dispersion estimation and false discovery rate control, approaches shown to improve performance at moderate sample sizes. We therefore interpret our findings with appropriate caution, emphasizing robust and biologically consistent signals.

Computation of emerging differentially expressed genes (eDEGs). eDEGs were identified based on the pipeline described below for both bulk RNA sequencing datasets (GSE269451, Suppl. Figure 2A; GSE304346, Suppl. Figure 20A). We first filtered DEG analysis results for cKO versus cWT samples at 11- and 25-dpi, based on meeting a significance threshold of *p*-adj < 0.05 and a base mean expression ≥ 10. We retained all 11-dpi genes that met the threshold (GSE269451: 1,405 genes, GSE304346: 2,668 genes) for downstream analysis, irrespective of fold-change (FC). We further refined the 25-dpi significance-filtered genes using a |log2 FC| ≥ 1 threshold (GSE269451: 2,951 DEGs, GSE304346: 3,426 DEGs), thus meeting DEG requirements outlined in the "Bulk RNA-seq analysis" section. We compared the 11-dpi genes and 25-dpi DEGs and identified genes with altered expression at 11-dpi that were also found at the 25-dpi time-point (GSE269451: 508 genes, GSE304346: 995 genes). ~17% of these genes (GSE269451: 85 DEGs, GSE304346: 175 DEGs) had a |log2 FC| ≥ 1, thus representing

11-dpi DEGs. Of the genes that overlapped with 25-dpi DEGs, 496 genes (GSE269451, 98%) and 83 genes (GSE304346, 8%) had the same directional change at both early and late timepoints (i.e., both up- or down-regulated) and were termed emerging differentially expressed genes (eDEGs). The remaining genes did not have the same directional expression change at both early and late timepoints (described in Suppl. Fig. S2C-D for GSE269451 and S20C for GSE304346).

Classification of DEG rescue by LINC complex disruption. To assess the extent to which LINC complex disruption rescues gene misregulation in cKO hearts, we analyzed DEGs identified across three pairwise comparisons:

> (1) cKO hearts without LINC complex disruption versus cWT hearts without LINC complex disruption (cKO – DN KASH vs. cWT – DN KASH) to identify genes misregulated in the diseased state (Suppl. Table S15),
> (2) cKO hearts without LINC complex disruption versus cKO hearts with LINC complex disruption (*Lna* cKO – DN KASH vs. cKO + DN KASH) to evaluate the impact of LINC complex disruption (Suppl. Table S16), and
> (3) cKO hearts with LINC complex disruption versus cWT hearts without LINC complex disruption (cKO + DN KASH vs. cWT – DN KASH) to determine whether gene expression returned to wild-type levels with LINC complex disruption (Suppl. Table S17).

Genes were classified as rescued if they were significantly misregulated in the disease (comparison 1) and significantly altered following LINC complex disruption (comparison 2). Complete rescue was defined as genes whose expression in cKO + DN KASH hearts was no longer significantly different from cWT (not significant in comparison 3). Partial rescue was defined as genes that remained significantly different from cWT despite changes following LINC complex disruption (significant in comparison 3).

Single-nucleus RNA sequencing and analysis. Single-nucleus RNA-sequencing (snRNA-seq) was chosen over single-cell RNA-sequencing for this study due to several key advantages that are particularly relevant in cardiac research[79]: (1) snRNA-seq minimizes bias in cell type capture related to cell size, which is particularly important for larges cells such as cardiomyocytes; (2) snRNA-seq excludes cytoplasmic RNA, reducing interference from high mitochondrial RNA content commonly found in cardiomyocytes; and (3) snRNA-seq is compatible with frozen tissue samples, eliminating the need for fresh specimens.

For processing, left ventricle free wall tissues (5-6 left ventricles per experimental group) were harvested from an independent subset of cKO and cWT mice at the appropriate timepoints, flash frozen in liquid nitrogen, and sent to SingulOmics (Bronx, NY, USA) for processing. There, nuclei were isolated from frozen samples and used to construct 3' single nuclei gene expression libraries (Next GEM v3.1) using the 10X Genomics Chromium system. Libraries were sequenced with ≈200 million PE150 reads per sample on Illumina NovaSeq 6000. Sequencing reads, including introns, were then aligned with the mouse reference genome (mm10/GENCODE vM23/Ensembl 98) using Cell Ranger[20]

(v7.0.1). The Debris Identification using Expectation Maximization (DIEM) algorithm[21] was applied to remove background, keeping barcodes with debris scores < 0.5, UMI count >800, and number of features (genes) > 250. The resulting dataset was composed of 22,043 total nuclei (5,052 11-dpi and 5,939 25-dpi in cKO; 6,300 11-dpi and 4,752 25-dpi cWT), before downstream filtering. snRNA-seq sample outputs from Cell Ranger were then loaded into R[80] (v4.1.1) and individually subjected to the standard Seurat[22] (v5.0.1) pipeline and sctransform. All samples were processed for ambient RNA (SoupX[81], v1.6.2) and doublet (DoubletFinder[82], v2.0.3) removal individually. Sample integration was performed with the integrated object subjected to the same standard Seurat pipeline as above, with a cluster resolution of 0.5. To annotate individual clusters with cell types using the established Azimuth reference map[23–25], mouse genes were first converted to human orthologous genes as only human heart references are available. The converted snRNA-seq dataset was then compared to the Azimuth reference map to assess prediction mapping scores[83]. Cells with high-confidence annotations (prediction scores > 0.75) were used to determine cell types in each identified cluster. To identify cell type-specific subpopulations, each cell type cluster was subjected to the standard Seurat (v5.0.1) pipeline and sctransform with unique cluster resolution (0.9 for cardiomyocyte, 0.7 for myeloid cells, 0.5 for fibroblasts, 0.6 for B cells, and 0.8 for NK/T cells). DEG analyses were performed for each cell type cluster using Seurat's FindMarkers function: 1) cKO vs. cWT at 11- and 25-dpi across all cells in the cluster irrespective of subpopulation, and 2) each subpopulation vs. all other subpopulations irrespective of experimental condition. Genes were required to meet a threshold of $|\log 2\ FC| \geq 0.1$, p-adj < 0.05, and for the fraction of cells expressing the gene to be ≥ 0.01 for at least one of the two queried populations. Gene modules scores were generated using Seurat's AddModuleScore function and gene lists curated by the gene ontology consortium[43,84] (GO: 0006954, 0031347, and 0008630), Harmonizome[85,86] (v3.0) (myofibroblast gene set from GeneRIF Biological Term Annotations), and peer-reviewed papers[57] (infiltrating and resident macrophage markers).

Cell-cell interaction prediction. Cell-cell communication prediction was determined using CellChat[26] (version 2.1.1). The CellChat algorithm computed the strength of specific predicted cell-cell interactions, quantified as the sum of the communication probability of all ligand-receptor interactions, in each experimental condition. CellChat objects were generated from Seurat snRNA-seq objects for each condition individually and processed using the default pipeline to identify enriched ligand-receptor interactions between all cell types and subpopulations. CellChat objects were then merged based on timepoints to perform a comparative analysis of cell-cell communication between cKO and cWT samples over time.

Pseudotime analysis. Pseudotime trajectories were inferred using Slingshot[27] (v2.2.1) applied to the PCA embedding of the integrated cardiomyocyte subpopulations. CM1 was designated as the root population based on its early timepoint relevance and CM3 as the terminal population due to emergence at later timepoints. These assignments were based on their temporal distribution: CM1 was consistently present across all conditions at 11-dpi, while CM3 emerged only at 25-dpi.

Functional enrichment analysis. DEGs and eDEGs Log2-fold change-ranked gene symbol lists were submitted to Metascape[28] (v3.5.20240101) and QIAGEN's Ingenuity Pathway Analysis (IPA) software[29] (v111725566) for functional enrichment analysis. All analyses used the gene ontology[43,84] (GO) biological processes database for enrichment. To perform comparative analyses between gene sets, multiple gene lists were provided simultaneously to Metascape using default parameters (i.e. p-value < 0.01, enrichment ≥ 1.5, overlap ≥ 3). Metascape calculates enrichment using a hypergeometric test. Comparative analyses of predicted upstream regulators between different gene sets were performed using IPA, assuming a |z-score| > 2 and *p*-value < 0.05 as predictive of regulator activation or inhibition.

Human DCM snRNA-seq dataset. Publicly available snRNA-seq data of genetic dilated cardiomyopathies[30] were obtained from the CZ CELLxGENE data portal[31] in h5ad format. We extracted samples annotated as "heart left ventricle" and classified as either "dilated cardiomyopathy" or "normal" using Scanpy[32] (v1.9.3) in Python (v3.7.12). The resulting AnnData object was exported by separating it into counts.mtx, obs.csv, and var.csv files. These files were subsequently imported into R to create a Seurat object, which was subjected to the standard Seurat (v5.0.1) pipeline and sctransform with cluster resolution of 0.4.

Tissue collection for Curio Seeker spatial transcriptomics. Isolated hearts were perfused and then coated in Tissue-Tek O.C.T. Compound (Sakura Finetek # 4583), inside cryomolds to be snap-frozen in liquid nitrogen cooled isopentane (Thermo Scientific Chemicals # AA19387AP) and stored at –80°C. Frozen hearts were sectioned with a cryostat at 10 μm thick slices at –20°C.

Spatial transcriptomic library preparation and sequencing for cardiac samples. Spatial transcriptomic libraries were prepared from heart tissue sections obtained from cKO and cWT mice at 11- and 25-days post injection (dpi). Samples were mounted directly onto barcoded Curio Seeker arrays (Curio Biosciences), corresponding to tile IDs B0040_010 (cKO, 11-dpi), B0040_012 (cWT, 11-dpi), B0040_011 (cKO, 25-dpi), and B0040_014 (cWT, 25-dpi). The protocol followed Curio's spatial transcriptomics workflow, beginning with RNA hybridization and in situ reverse transcription on the array. Following enzymatic tissue digestion, spatially barcoded beads were released into solution, and second-strand cDNA synthesis and amplification were performed. Sequencing libraries were constructed using the Nextera XT DNA kit (Illumina #FC-131-1024), pooled, and sequenced on an Illumina NovaSeq X Plus platform (Read ½: 150 bp, Index ½: 8 bp). Raw sequencing data were processed using the slide_snake pipeline (https://github.com/mckellardw/slide_snake, v0.1). Adapter trimming and quality filtering were performed using the seeker_v3.1_internalTrim configuration (cutadapt v4.6)[87], and reads were aligned to the mouse genome (GRCm39) using STARsolo (v2.7.10b) to generate bead-resolved gene expression matrices[33].

Curio Seeker data preprocessing and quality control. For each sample, AnnData objects (v0.10.3) were generated from the raw count matrices, and spatial coordinates were assigned to beads using metadata provided by Curio Biosciences (also known as

slide-seq[88]). Preprocessing was performed using Scanpy[32] (v1.9.3), which included filtering out beads with fewer than 200 detected transcripts, normalizing expression values to a fixed total count per bead, and log transformation. Beads with fewer than 10 neighbors within a 100 μm radius were also excluded, as these often represent smeared or low-quality barcodes. Data were plotted, and regions of interest corresponding to the left ventricles were segmented using Napari[89]. To further reduce noise while preserving spatial structure, expression data were smoothed using the Smoothie package (https://github.com/caholdener01/Smoothie, v1.0.0) implementation with a Gaussian kernel of 30 μm[90].

Cell type deconvolution. We applied cell2location[34] (v0.1.3) to deconvolve the collected spatial transcriptomics data using the single-nucleus dataset discussed in this work as a reference. Cell-subpopulation-specific expression signatures were estimated from the reference and projected onto the spatial data to infer the contribution of each cell subpopulation category in the spatial beads. Cell2location was run either on full heart sections or focused specifically on the left ventricle annotated regions. Model hyperparameters were set to N_cells_per_location = 1 and detection_alpha = 20.

Cardiomyocyte neighbor score calculation. To assess the local enrichment of specific cardiomyocyte populations, we implemented a k-nearest neighbors (kNN) approach. For each bead, we computed a Cardiomyocyte neighbor score by counting how many of its 10 nearest spatial neighbors shared the same predicted cardiomyocyte subpopulation (CM1 to CM5). This analysis was performed independently for each of the segmented ventricular spatial transcriptomics objects.

Neighborhood enrichment analysis on spatial transcriptomics data. Z-score calculations of neighborhood enrichment was calculated using Squidpy[35] (v1.2.2) for all experimental conditions. A heatmap of this enrichment was generated with Seaborn[91] (v0.12.2). Finally, visualization of individual subpopulations across the tissue was generated through Squidpy.

Gene-set score calculation on spatial transcriptomics data. Gene set activity scores were then computed in Scanpy with default parameters for curated programs related to regulation of defense, interferon-beta response, intrinsic apoptosis, and inflammatory response. For each left ventricle sample, scores were compared across five cardiomyocyte subpopulations (CM1–CM5). To evaluate whether these distributions differed significantly within each heart, a Kruskal–Wallis H-test was first performed. In cases where the Kruskal–Wallis test returned a P value < 0.05, pairwise Mann–Whitney U tests were conducted to identify specific group differences. All pairwise p-values were adjusted for multiple testing using the Benjamini–Hochberg false discovery rate (FDR) correction, applied independently within each heart sample. All tests were two-sided and implemented using the scipy.stats and statsmodels.stats Python libraries.

Statistical analysis. Animals were randomly assigned to treatment groups, and experiments were conducted blinded to treatment and genotype. Since Cre expressed in the heart can be cardiotoxic at high tamoxifen doses, experiments used only low

tamoxifen doses (30 mg/kg/day). Furthermore, for maximum stringency, comparisons are focused on groups in which all mice express the MCM transgene but differ in the status of the floxed *Lmna* gene. Tissues isolated from individual mice were counted as a single replicate (unless otherwise specified). Since the bulk-RNA sequencing data did not show any sex difference, male and female mice were combined for downstream transcriptomic analysis. Survival curves were generated using the Kaplan–Meier method and differences in survival tested with a Breslow test using GraphPad Prism 10. For results that follow a Gaussian distribution, the mean and standard error of the mean (SEM) were calculated; ANOVA with Tukey or Dunnett post-analysis was used to test for statistical significance between more than two experimental groups. When group sizes were N < 6, non-parametric tests were performed. Details regarding the statistical analysis for each graph are included in the corresponding figure legends Data linearity was determined using Pearson correlation coefficients and significance calculated using the R package ggpubr[92]. The non-parametric pairwise wilcoxon rank sum test was calculated to determine significant changes in gene module scores between conditions in R. Finally, the concordance correlation coefficient (CCC) was calculated using the equation:

$$\frac{2*\sigma_{12}}{(\mu_1-\mu_2)^2+\sigma_1{}^2+\sigma_2{}^2}$$

where σ_{12} is the covariance, μ is the mean, and σ is the standard deviation.

Supplementary Figures

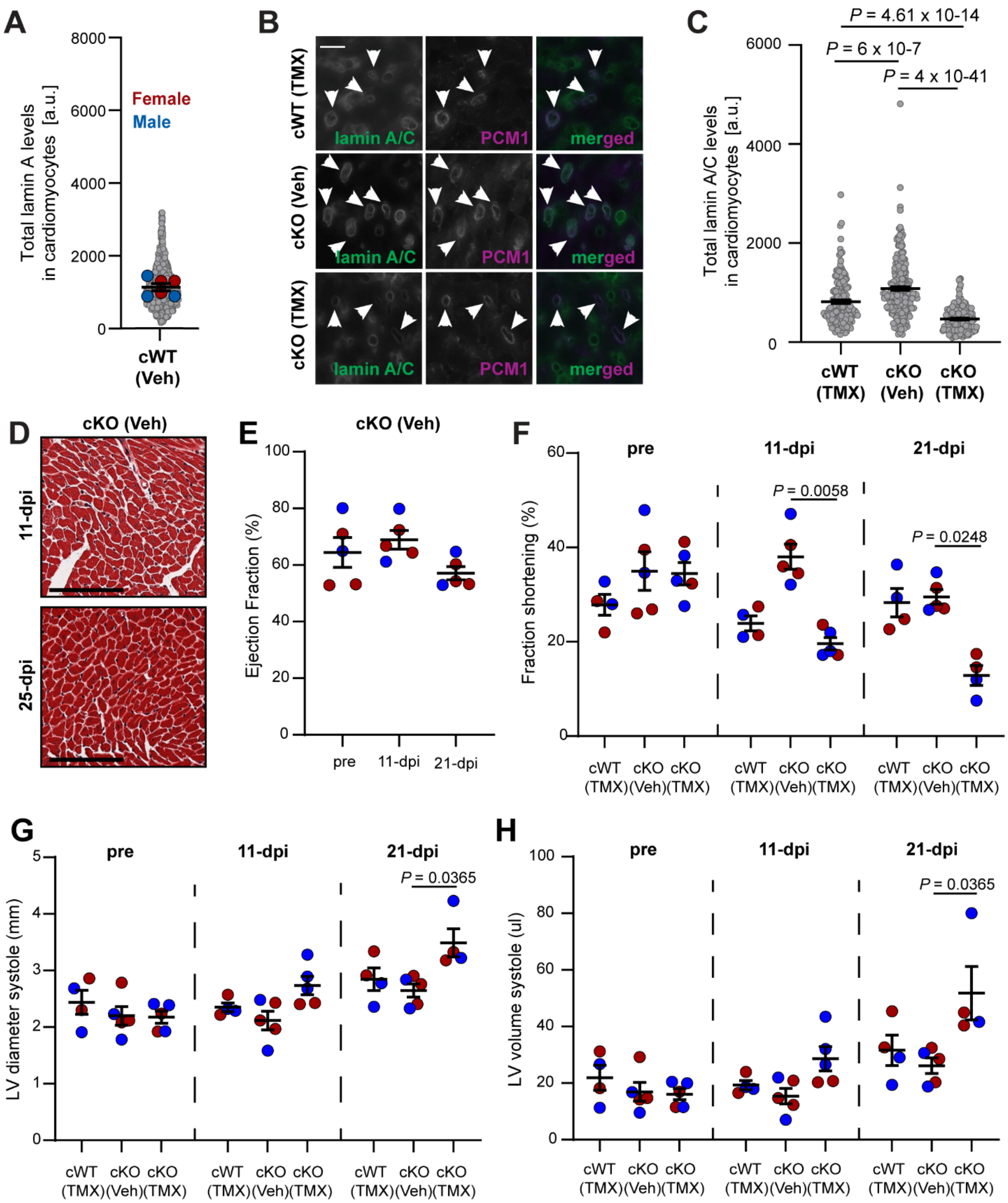

Supplementary Figure S1: Reduction of lamin A/C in cardiomyocytes in adult mice leads to dilated cardiomyopathy. (A) Quantification of total lamin A levels in vehicle-only treated cKO cardiomyocytes. Larger colored circles represent average values for individual mice. Smaller gray circles represent individual cardiomyocytes. *N* = 6

mice/condition. **(B)** Representative images of cardiac tissues from cWT mice treated with 30 mg/kg tamoxifen (TMX) (top), cKO mice treated with vehicle (Veh) control (middle), and cKO mice treated with 30 mg/kg tamoxifen (bottom), immunofluorescently labeled for lamin A (green) and PCM1, a cardiomyocyte-specific nuclear marker (magenta). Scale bar: 20 µm. Arrows indicate representative cardiomyocytes. **(C)** Quantification of total lamin A/C levels in cardiomyocytes from vehicle-only-treated cKO and tamoxifen-treated cKO and cWT hearts, confirming the depletion of lamin A/C in cardiomyocytes of tamoxifen-treated cKO mice. Gray circles represent individual cardiomyocytes (1 mouse per condition). **(D)** Masson's trichrome staining of heart sections to detect fibrosis in vehicle-only-treated cKO hearts at both early (11-dpi) and late (25-dpi). Scale bar: 100 µm. Echocardiography-based measurements of **(E)** left ventricular ejection fraction (EF), given as percentage, for vehicle-only treated cKO mice, and **(F)** fractional shortening (FS), given as percentage, **(G)** left ventricle (LV) diameter during systole, measured in mm, and **(H)** left ventricular volume during systole, measured in mm for vehicle-only treated cKO mice, and tamoxifen-treated cKO and cWT mice . Symbols represent the mean values of individual mice. Error bars represent mean ± S.E.M. **(A, E-H)** Color indicates sex of individual mice (red: female, blue: male). N = 4-5 mice/condition. Statistical tests: **(C, E, F-H)** Kruskal-Wallis test with Dunn's post-hoc test.

.

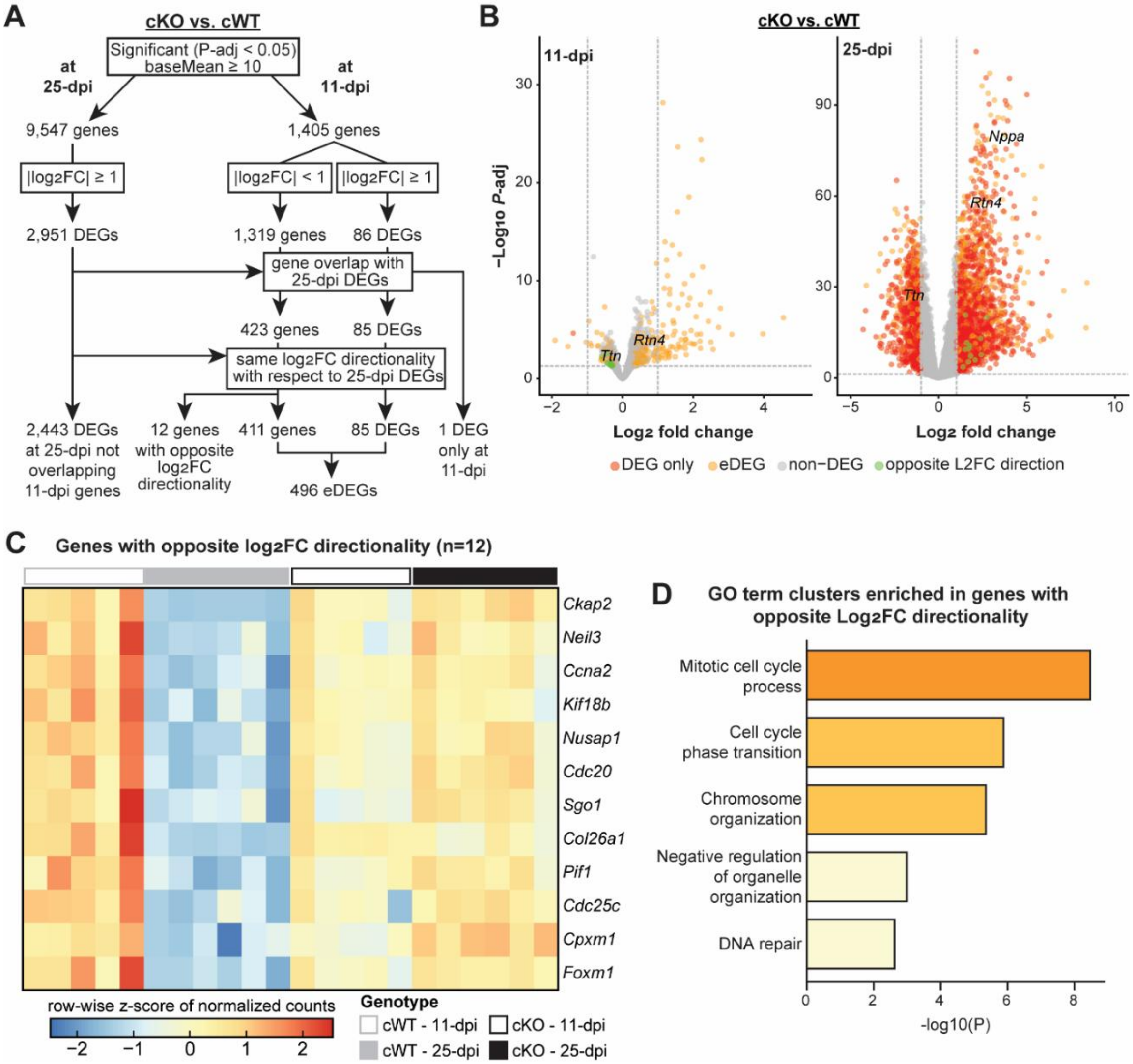

Supplementary Figure S2: eDEG pipeline and analysis in bulk RNA-seq data of left ventricular tissue. (A) Flow chart demonstrating the steps to identify emerging differentially expressed genes (eDEGs) from bulk RNA-seq data, based on $\log_2$-fold change (Log_2FC) and adjusted *P*-values (*P*-adj) generated when comparing the gene expression between cKO and cWT samples at each timepoint. **(B)** Volcano plot of the cKO versus cWT DEG analysis at 11- (left) and 25-days post injection (dpi) (right). Genes meeting DEG requirements (*P*-adj < 0.05 and $|\text{Log}_2\text{FC} \geq 1|$) but not defined as eDEG are represented in red, eDEGs are shown in orange, the 12 genes with opposite L2FC directionality between 11- and 25-dpi are shown in green, and genes not meeting either DEG or eDEG requirements are shown in gray. Select genes identified throughout the study are highlighted. **(C)** Heatmap of row-wise *z*-score normalized read counts for the 12 genes which had opposite Log_2FC directionality (cKO versus cWT) at 11- and 25-dpi. Columns, indicating individual samples, were sorted by experimental conditions denoted below by color. Gene names are highlighted. **(D)** Bar plot of gene ontology (GO) term clusters enriched in the 12 genes with opposite Log_2FC directionality between the 11- and 25-dpi timepoints.

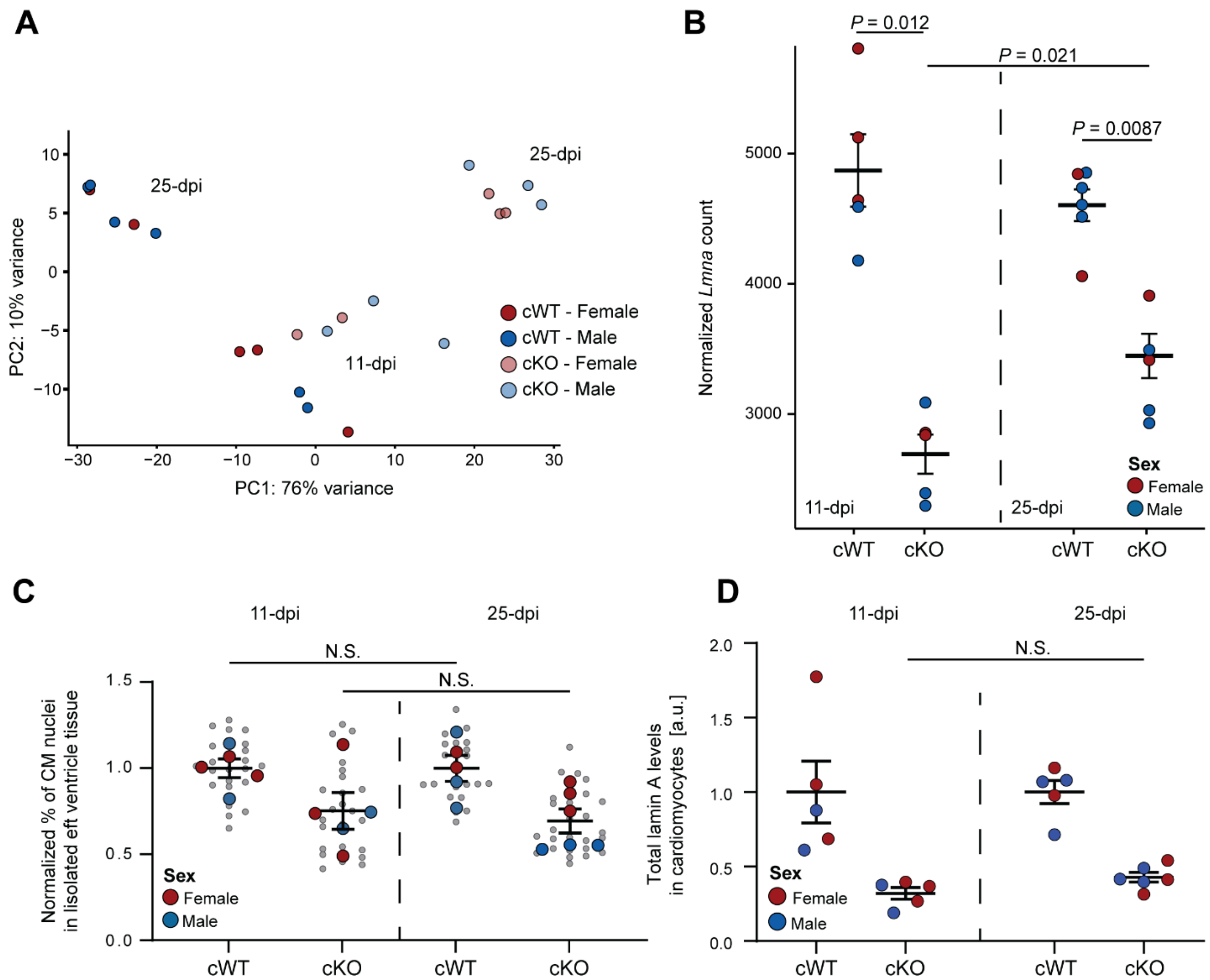

Supplementary Figure S3: Transcriptomic and immunofluorescence studies of *Lmna*-cKO and *Lmna*-cWT hearts did not show significant sex differences. (A) Principal components analysis (PCA) plot of bulk RNA-seq sample from all experimental conditions. *N* = 5-6 mice/condition. **(B)** Quantification of normalized *Lmna* read counts from bulk RNA-seq samples of cKO and cWT at 11- (left) and 25-dpi (right). Mean (thick black line) and standard error of the mean (thin black lines) are represented. *N* = 5-6 mice/condition. **(C)** Quantification of the normalized percentage of cardiomyocytes (CM) in 11-days post injection (dpi) (left) and 25-dpi (right) left ventricles. Gray dots represent individual fields of view images (10/mouse), large dots represent the average values for each individual mouse analyzed. *N* = 5 mice/group. **(D)** Quantification of lamin A levels in cardiomyocytes at 11-dpi (left) and 25-dpi (right). *N* = 5 mice/group. **(A-D)** Color indicates sex of individual mice (red: female, blue: male). Statistical tests: **(B)** Kruskal–Wallis tests followed by pairwise Mann–Whitney U tests with Benjamini-Hochberg correction, **(C, D)** Kruskal-Wallis test with Dunn's post-hoc test. N.S. = not significant

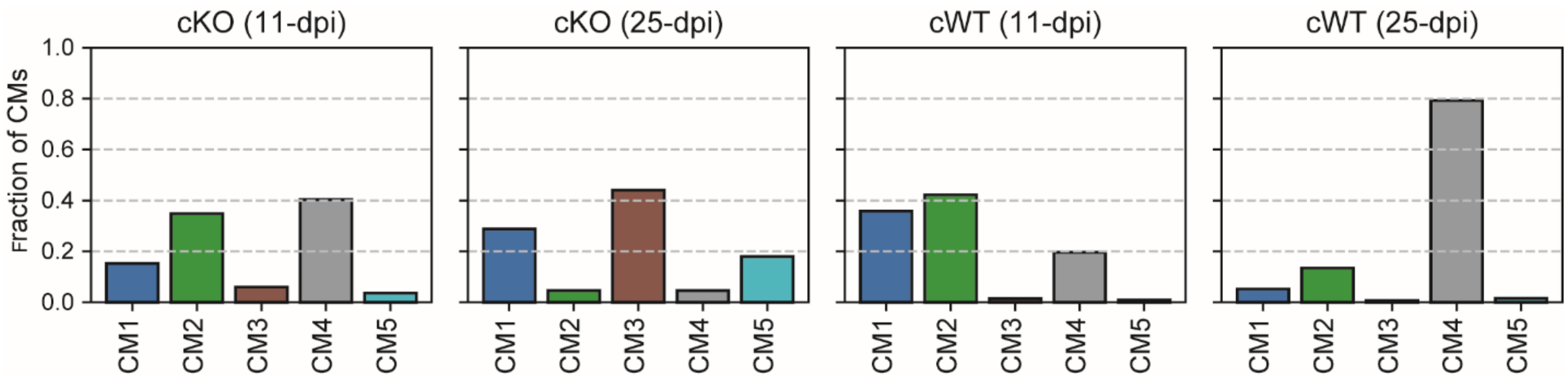

Supplementary Figure S4: Spatial transcriptomics analysis confirms the presence of disease-specific cardiomyocyte subpopulations. Fraction of Curio Seeker (Slide-seq) spots, 10 µm spatially barcoded locations on a slide array, assigned to each cardiomyocyte state (CM1–CM5) in the left ventricle of cKO and control cWT hearts at 11-dpi and 25-dpi. Fractions were computed within each sample by normalizing the number of spots assigned to each CM state to the total number of cardiomyocyte-labeled spots in that ventricle.

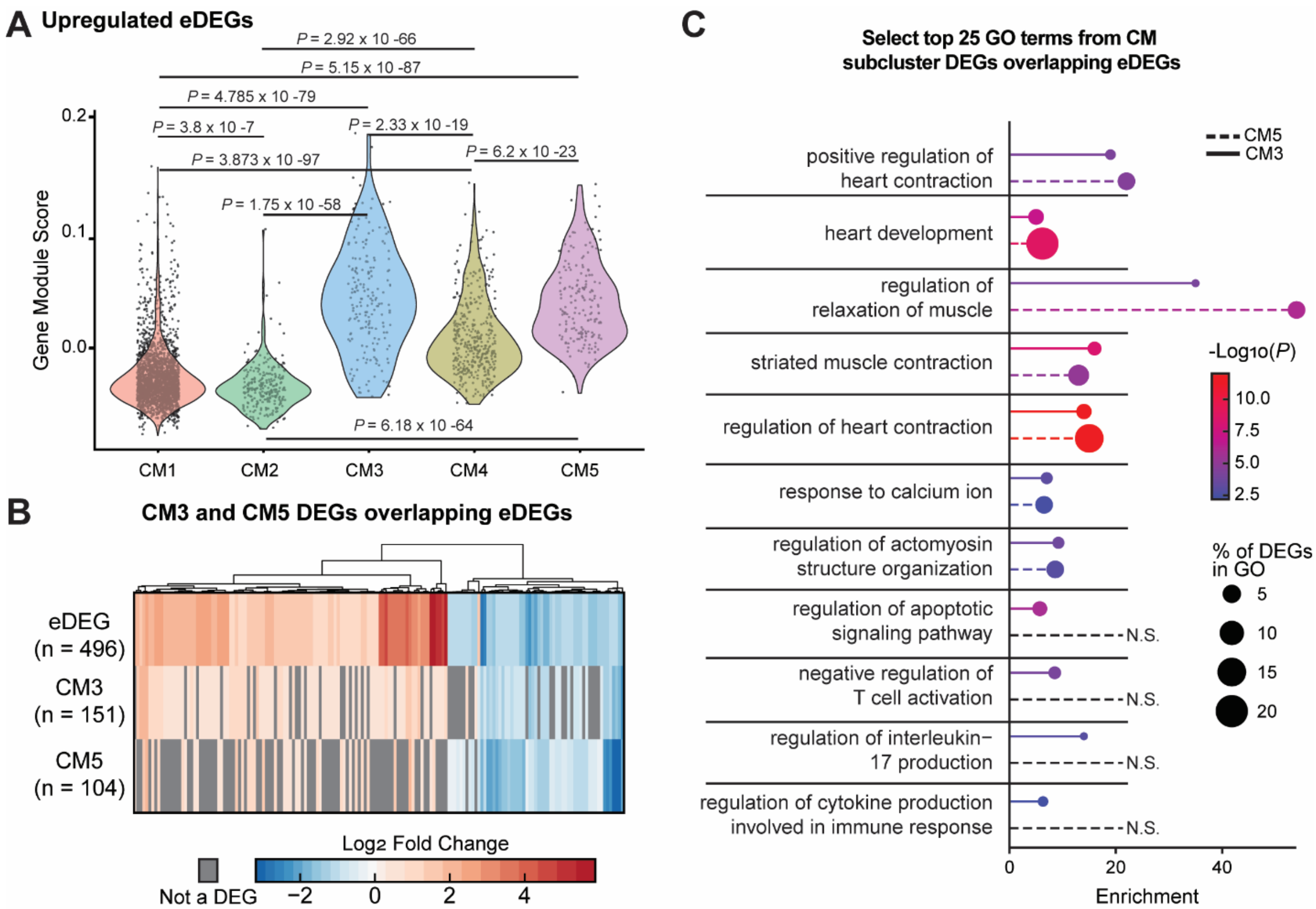

Supplementary Figure S5: Disease-specific cardiomyocyte subpopulations are enriched for eDEGs. (A) Violin plot of gene module scores for gene list consisting of emerging differentially expressed genes (eDEGs) upregulated between cKO and cWT at both timepoints across individual cardiomyocyte subpopulations (CM1-5) in the integrated snRNA-seq ventricle dataset. Dots represent individual nuclei. **(B)** Heatmap of log2 fold change for bulk RNA-seq eDEGs that overlap cardiomyocyte subcluster CM3 and CM5 subcluster-specific DEGs. Gray boxes indicate genes that were not subcluster-specific DEGs for CM3 or CM5. **(C)** Lollipop plot of select top 25 Gene Ontology (GO) terms enriched in disease-specific cardiomyocyte (CM) subpopulations, CM3 (continuous line) and CM5 (dashed line), differentially expressed genes (DEGs) that overlap eDEGs. Bar indicates enrichment score, while dot color shows significance of enrichment, and dot size represents the percentage of subpopulation-specific DEGs contained within the term. Non-significant (N.S.) terms are highlighted. Statistical test: **(A)** pair-wise Wilcoxon rank sum test with Benjamini-Hochberg correction.

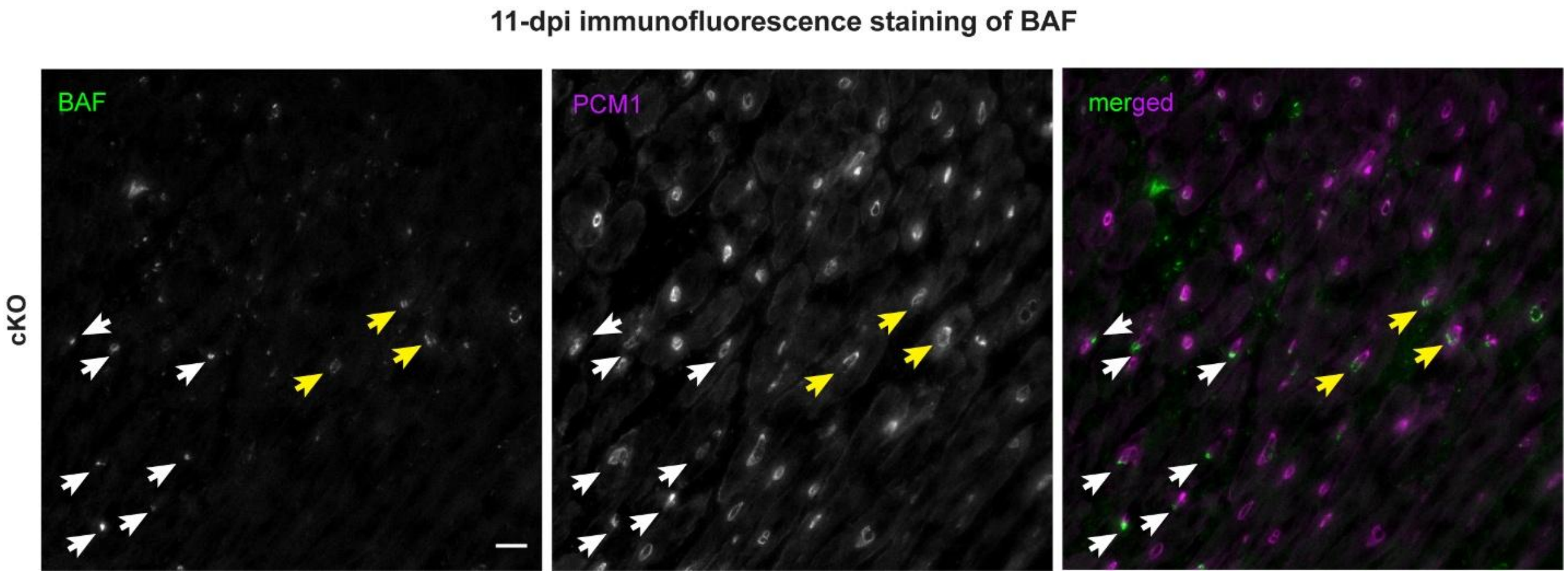

Supplementary Figure S6: *Lmna*-deficient cardiomyocytes have increased rates of nuclear envelope rupture. Representative images of cardiac tissue sections from cKO mice at 11 days post injection with antibodies against the nuclear envelope rupture marker BAF (green) and the cardiomyocyte specific marker PCM1 (magenta). Scale bar 20 µm. Arrows indicate representative cardiomyocytes positive for nuclear envelope rupture. Yellow arrows point to the nuclei depicted in the main text figure 3A.

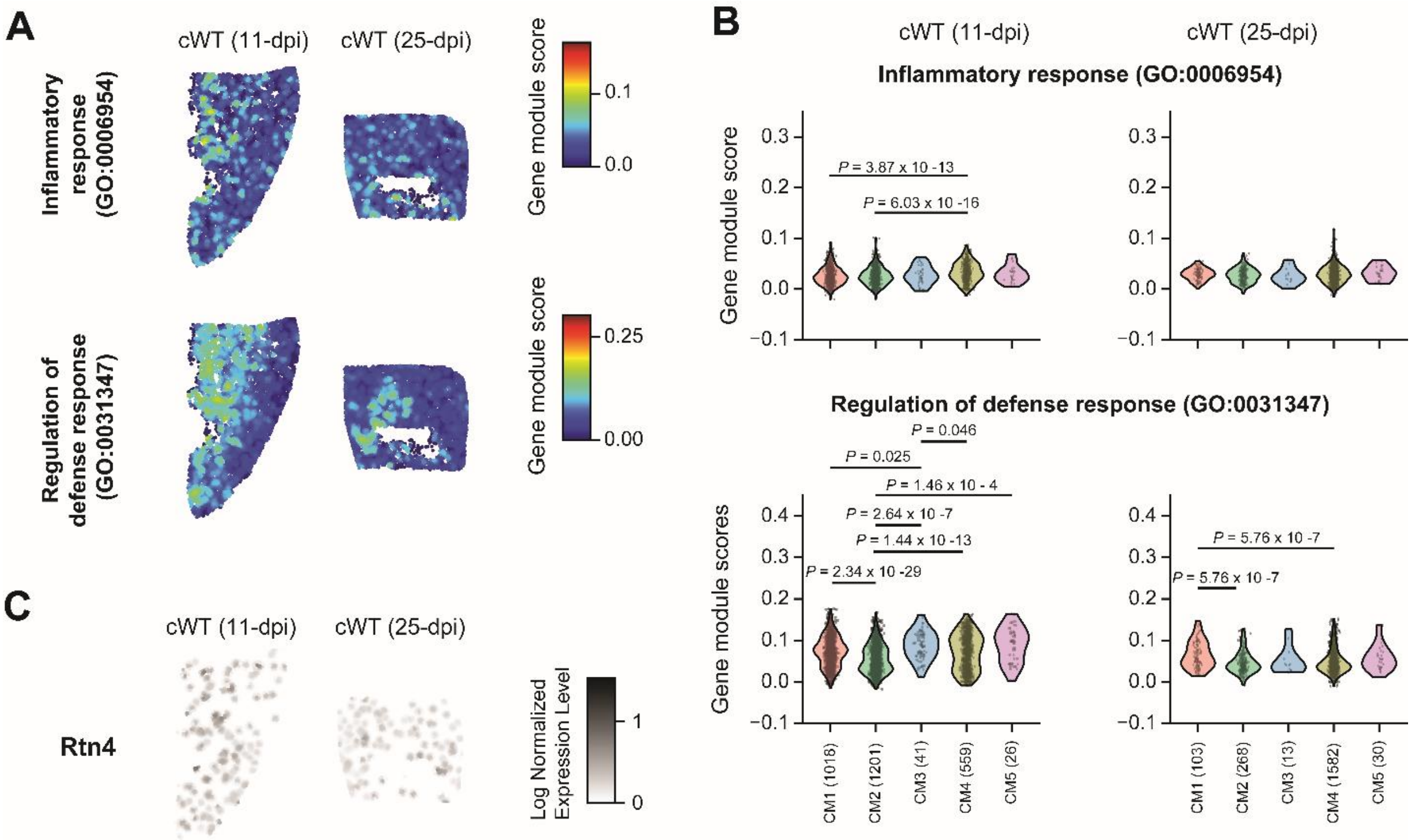

Supplementary Figure S7: Spatial transcriptomics analysis reveals enrichment of inflammatory and cytosolic DNA sensing pathways in cardiomyocyte subpopulations specific to cKO hearts. (A) Heatmap and **(B)** violin plots of gene module scores for GO terms "Inflammatory response" and "regulation of defense response" across **(A)** the left ventricle and **(B)** CM1-5 of 11- and 25-days post injection (dpi) cWT heart tissue slices based on spatial transcriptomics. Module scores were computed on smoothed, normalized gene expression data. **(B)** Sample size (number of spots per CM group) is indicated below each label. Dots represent individual spots in the array. **(C)** Heatmap of $\log_2$ normalized *Rtn4* expression across the left ventricle of 11-dpi (left) and 25-dpi (right) cWT and cKO heart tissue slices based on spatial transcriptomics. Statistical tests: **(B)** Kruskal–Wallis tests followed by pairwise Mann–Whitney U tests with Benjamini–Hochberg correction.

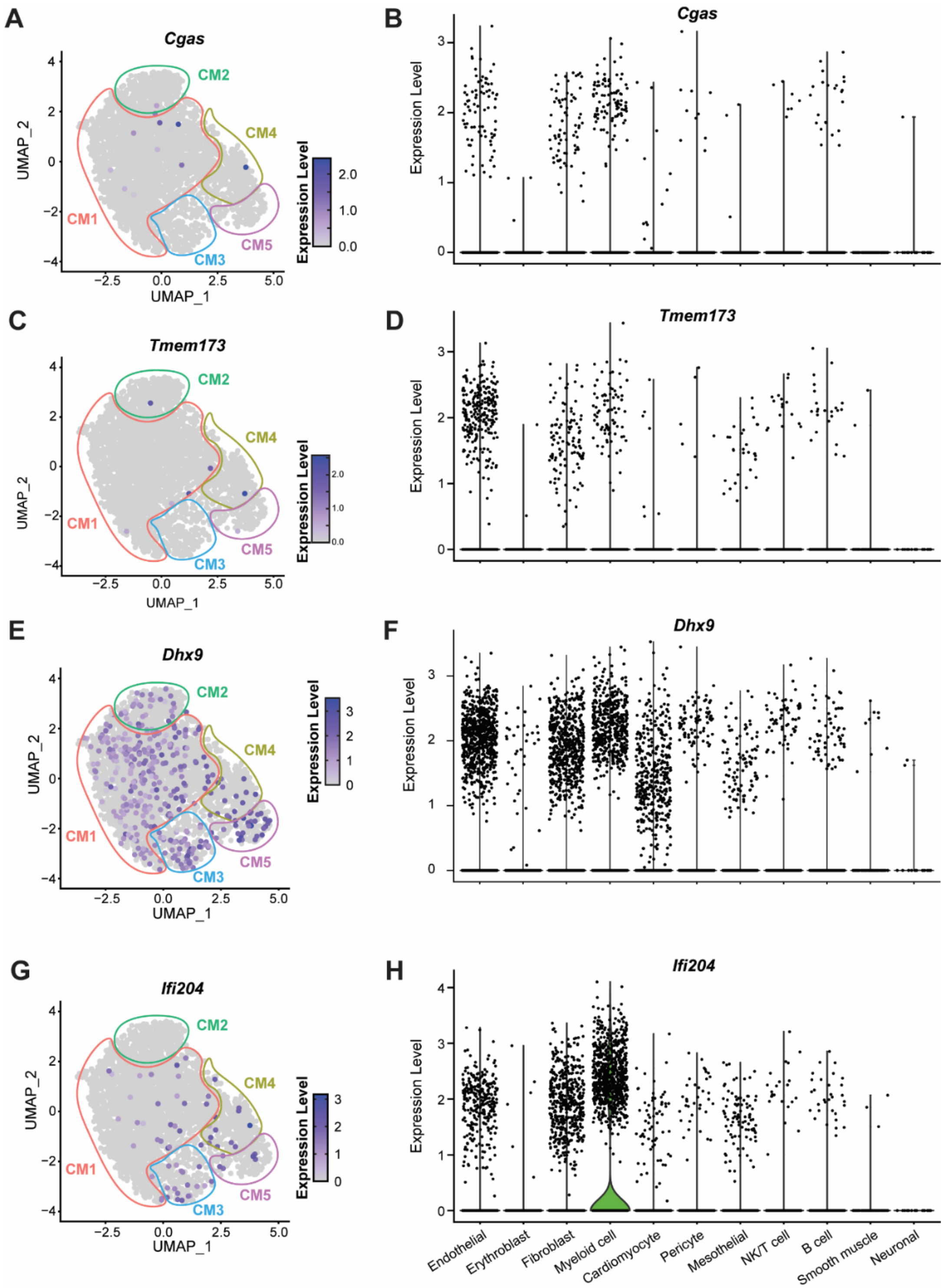

Supplementary Figure S8: Cardiomyocytes lack expression of *Cgas* and *Sting1*. Normalized expression levels for **(A-B)** *Cgas*, **(C-D)** *Sting1* (*Tmem73*), **(E-F)** *Dhx9*, and **(G-H)** *Ifi204* based on single-nucleus RNA sequencing (snRNA-seq) data **(A,C,E,G)** across the UMAP of all captured cardiomyocyte nuclei and **(B,D,F,H)** across all cell types in violin plots. **(A,C,E,G)** Circles of color in the UMAP plots indicate limits of cardiomyocyte subclusters (CM1-5).

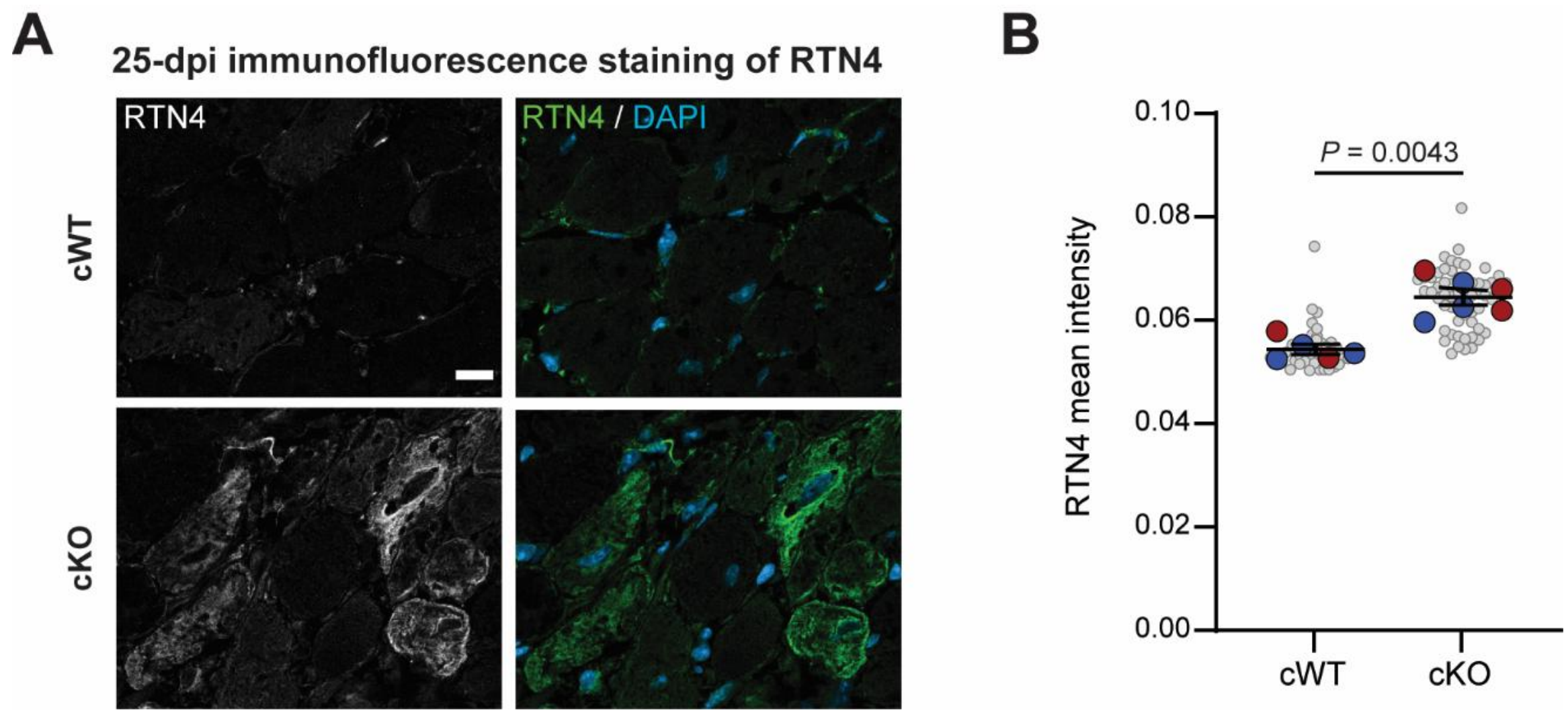

Supplementary Figure S9: Protein quantifications support transcriptomic findings of increased immune cell activation through cytosolic DNA sensing in cKO hearts. (**A**) Immunofluorescence labeling for RTN4 (gray on the left, green on the right) and nuclei using DAPI (blue) for cWT (top) and cKO (bottom) hearts at 25-dpi. Scale bar 20 µm. (**B**) quantification of mean RTN4 intensity from representative images. Larger colored circles represent average values for individual male (blue) or female (red) mice. *N* = 3 animals per genotype. Statistical test: Mann-Whitney test.

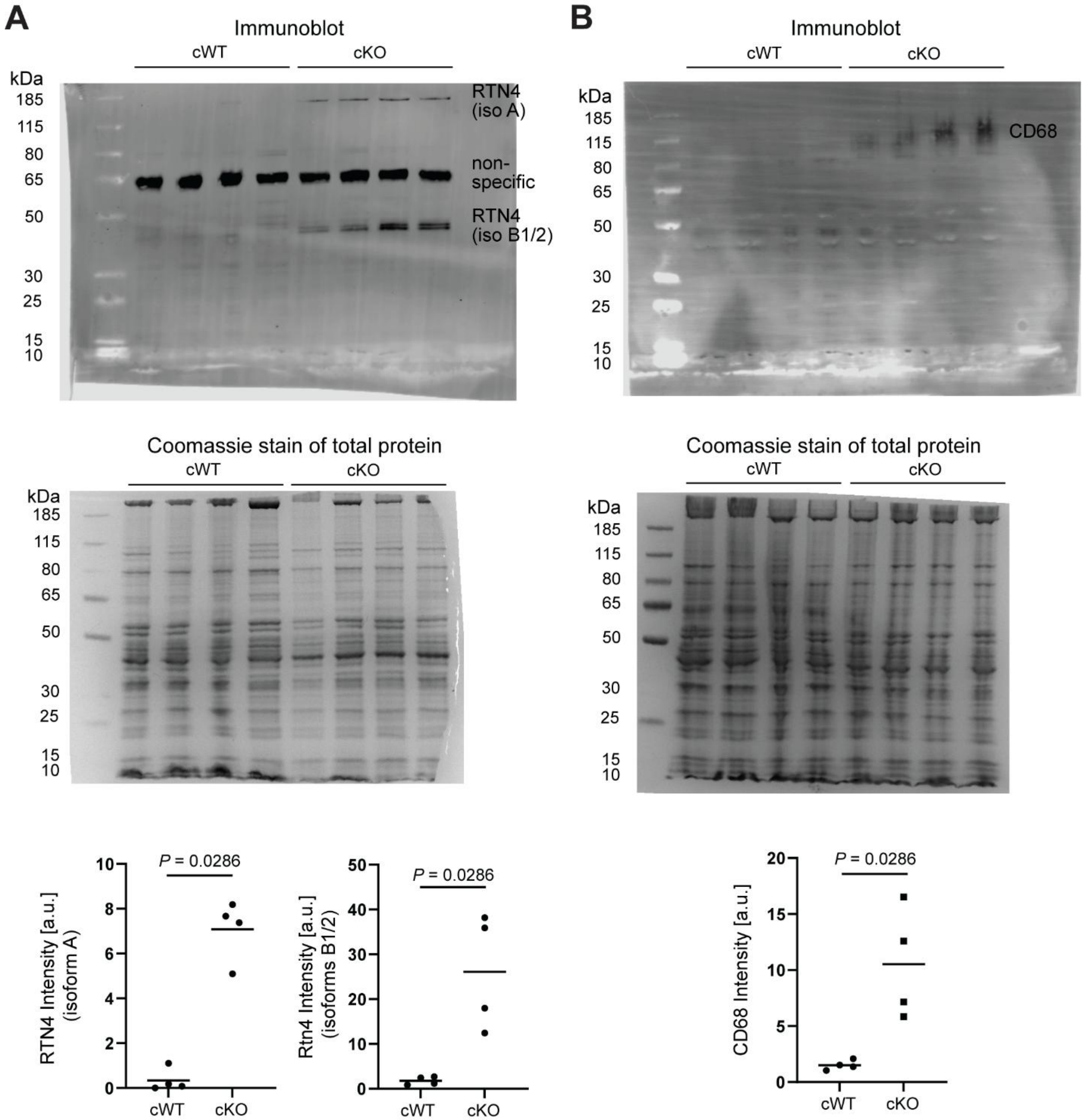

Supplementary Figure S10: Protein quantifications support transcriptomic findings of increased immune cell activation through cytosolic DNA sensing in cKO hearts. Immunoblot (top) of heart tissue protein lysates following immunoprecipitation with **(A)** anti-RTN4 and **(B)** anti-CD68 antibodies. Coomassie stains of western gels (middle) were used to ensure even protein loading and for normalization. Quantification (bottom) of band intensity, normalized to coomassie intensity, for replicates showing significant increased protein levels in cKO hearts versus cWT controls. **(A)** Individual isoforms of RTN4 (A and B1/2) are denoted in the gel. *N* = 4 animals per genotype. Statistical test: Mann-Whitney test.

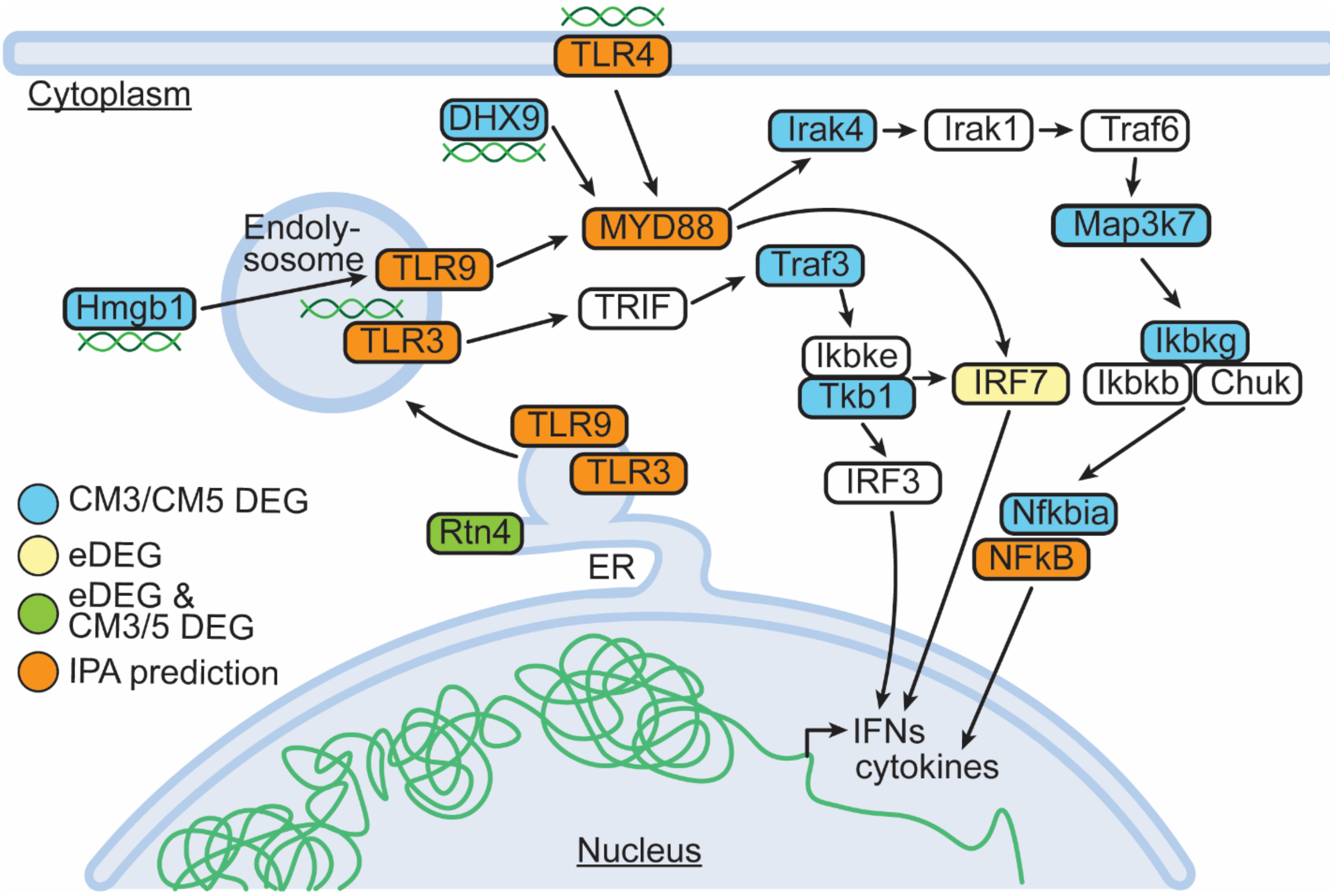

Supplementary Figure S11: Disease-specific cardiomyocyte subpopulations exhibit evidence for activation of cytosolic DNA sensing independent of cGas/STING pathways. Diagram of the Tool-like receptor (TLR)-mediated cytoplasmic pattern recognition receptor (PRR) signaling pathway in the cell interior, curated from multiple sources[43–46]. Colors around protein names indicate whether these were detected in the single nucleus RNA-seq cardiomyocyte subpopulations CM3 and CM5 DEGs (blue), appeared as an eDEG from the bulk RNA-seq dataset (yellow), appeared as both an eDEG and CM3/CM5 DEG (green), were predicted as an upstream regulator in Ingenuity Pathway Analysis (IPA) using genes that are both eDEG and CM3/CM5 DEGs (orange), or met none of these criteria (white). Green lines represent nuclear, cytoplasmic, and extracellular DNA.

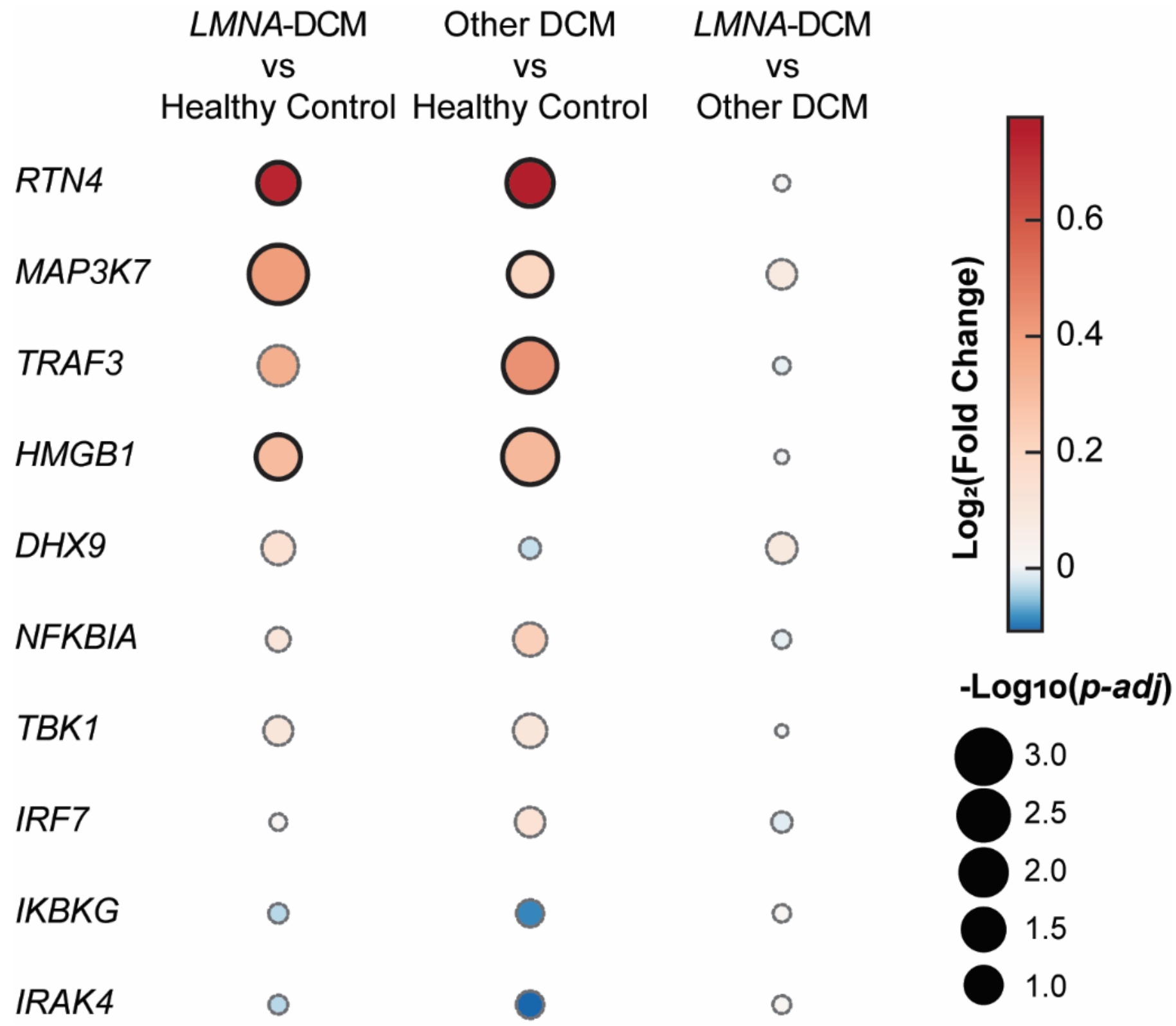

Supplementary Figure S12: Cytoplasmic pattern recognition receptor (PRR) signaling is upregulated in several forms of human dilated cardiomyopathy (DCM). Dot plot illustrating differential gene expression from pseudobulk snRNA-seq analysis of human left ventricle cardiomyocytes[29] comparing *LMNA*-DCM, other genetic DCMs, and healthy controls. Shown are cytoplasmic PRR genes identified as either eDEGs or CM3/CM5 DEGs (corresponding to the green, blue, and yellow gene sets in Suppl. Figure S11). Dot color represents effect size ($\log_2$ fold change), while dot size reflects statistical significance ($-\log_{10}$ *p-adj*) for the comparison indicated in each column. Genes reaching significance (i.e. $-\log_{10}$(*p-adj*) < 0.05) are outlined with solid black borders. Statistical test: Wald test and Benjamini-Hochberg correction implemented through DESeq2.

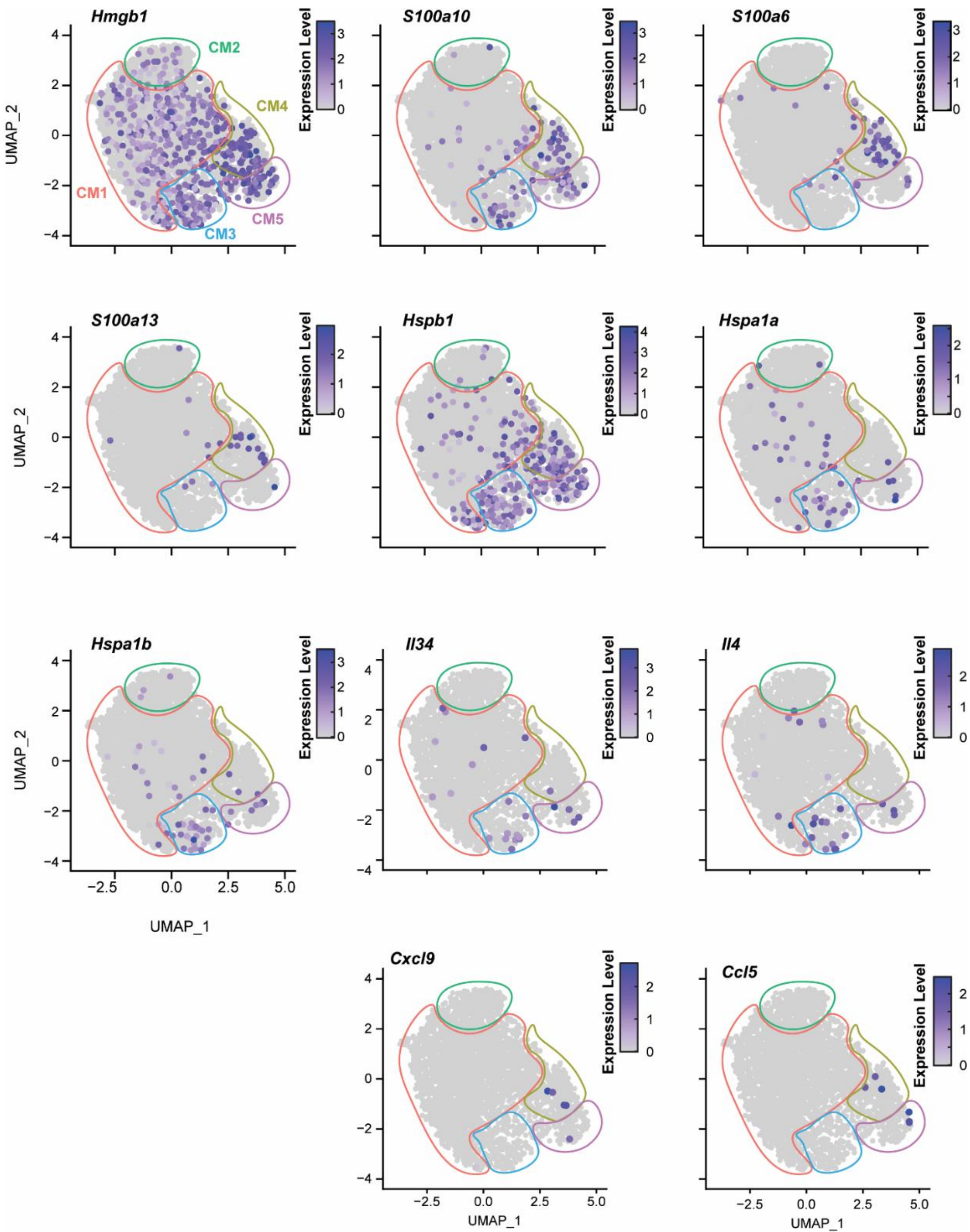

Supplementary Figure S13: Disease-specific cardiomyocyte subpopulations have increased expression of DAMPs and cytokines. Expression of various damage-associated molecular pattern (DAMP) (*Hmgb1, S100a10, S100a6, S100a13, Hspb1, Hspa1a, Hspa1b*) and cytokine (*Il34*, *Il4*, *Cxcl9*, *Ccl5*) genes in snRNA-seq data across the UMAP of all captured cardiomyocyte nuclei. Circles of color indicate limits of cardiomyocyte subclusters (CM1-5).

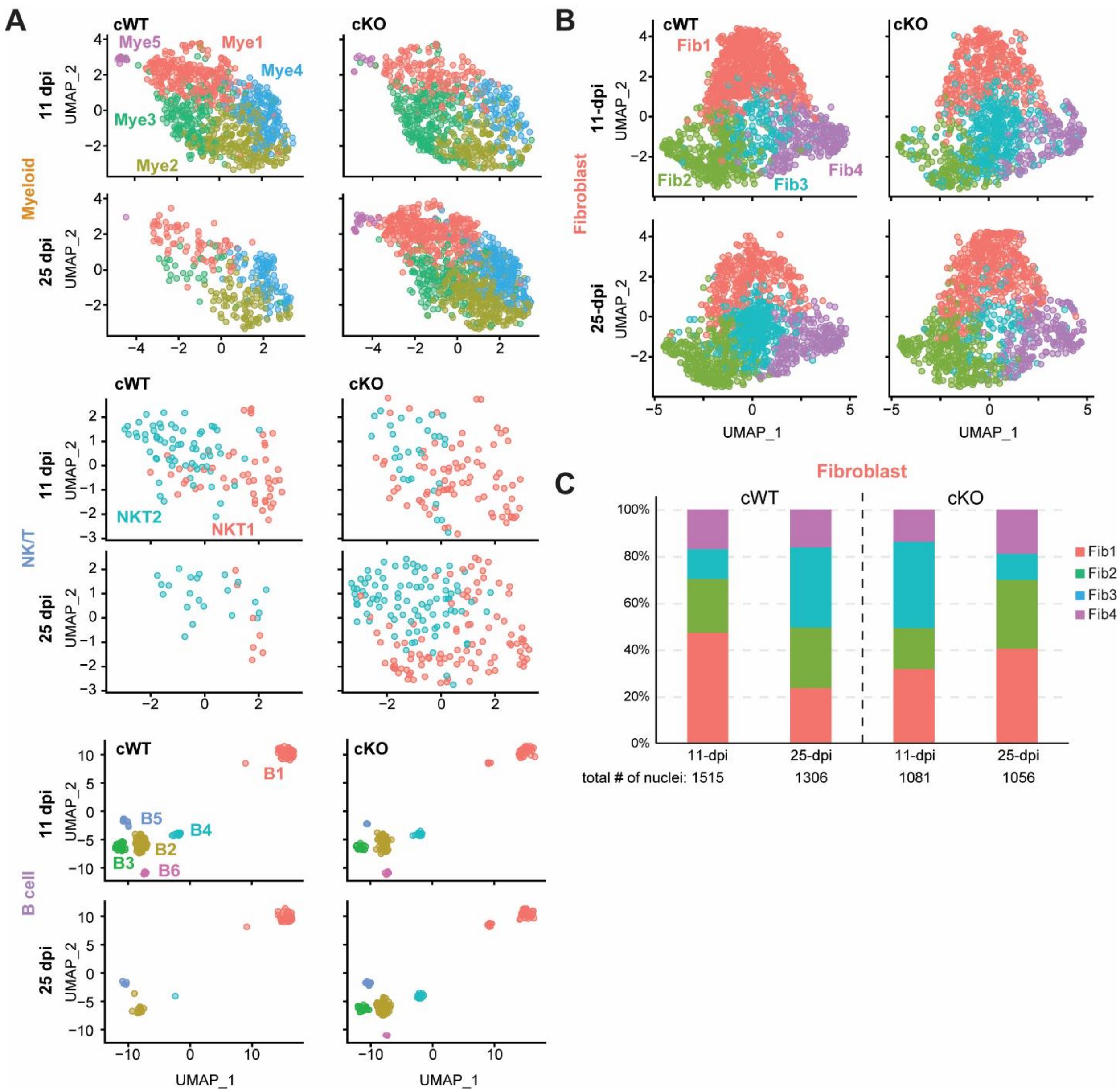

Supplementary Figure S14: Non-cardiomyocyte cells exhibit distinct subpopulation compositions in cKO hearts. (A,B) UMAP plots of **(A)** immune cells (Myeloid cells – top, Natural killer T cells – middle, and B cells – bottom) and **(B)** fibroblast-annotated nuclei from integrated snRNA-seq data, across genotype (left and right) and time point (top and bottom). Individual colors represent unique subpopulation identified. **(C)** Stacked bar plot showing the percent composition of each fibroblast (Fib) subcluster for every experimental condition.

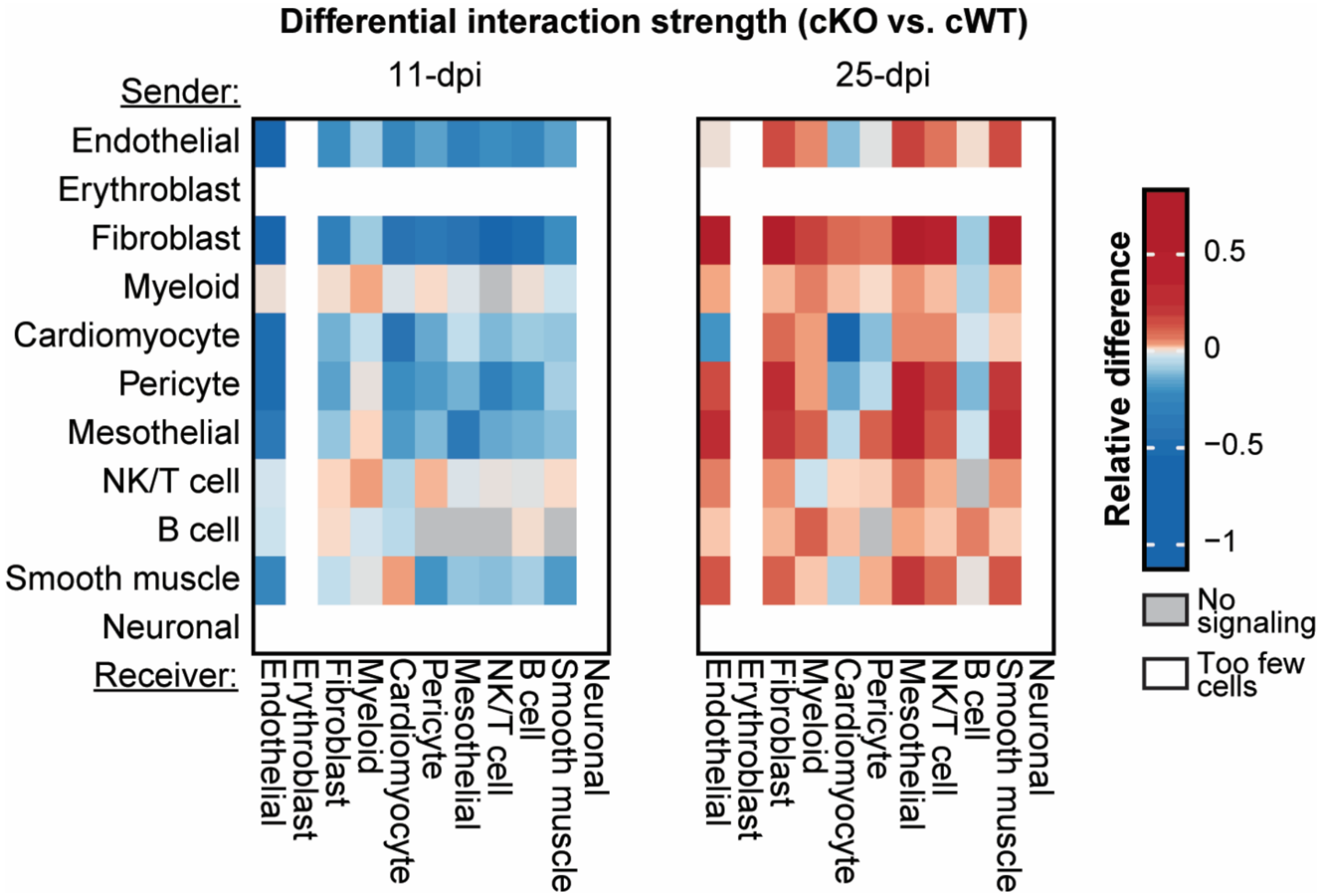

Supplementary Figure S15: Cell-cell communication across all captured cell types. Heatmap showing relative difference (cKO versus cWT) in the interaction strength of sender (rows) and receiver (columns) cell types at 11- (left) and 25-dpi (right). Interactions where no communication was identified (gray) and where too few cells were available to perform communication predictions (white) are shown.

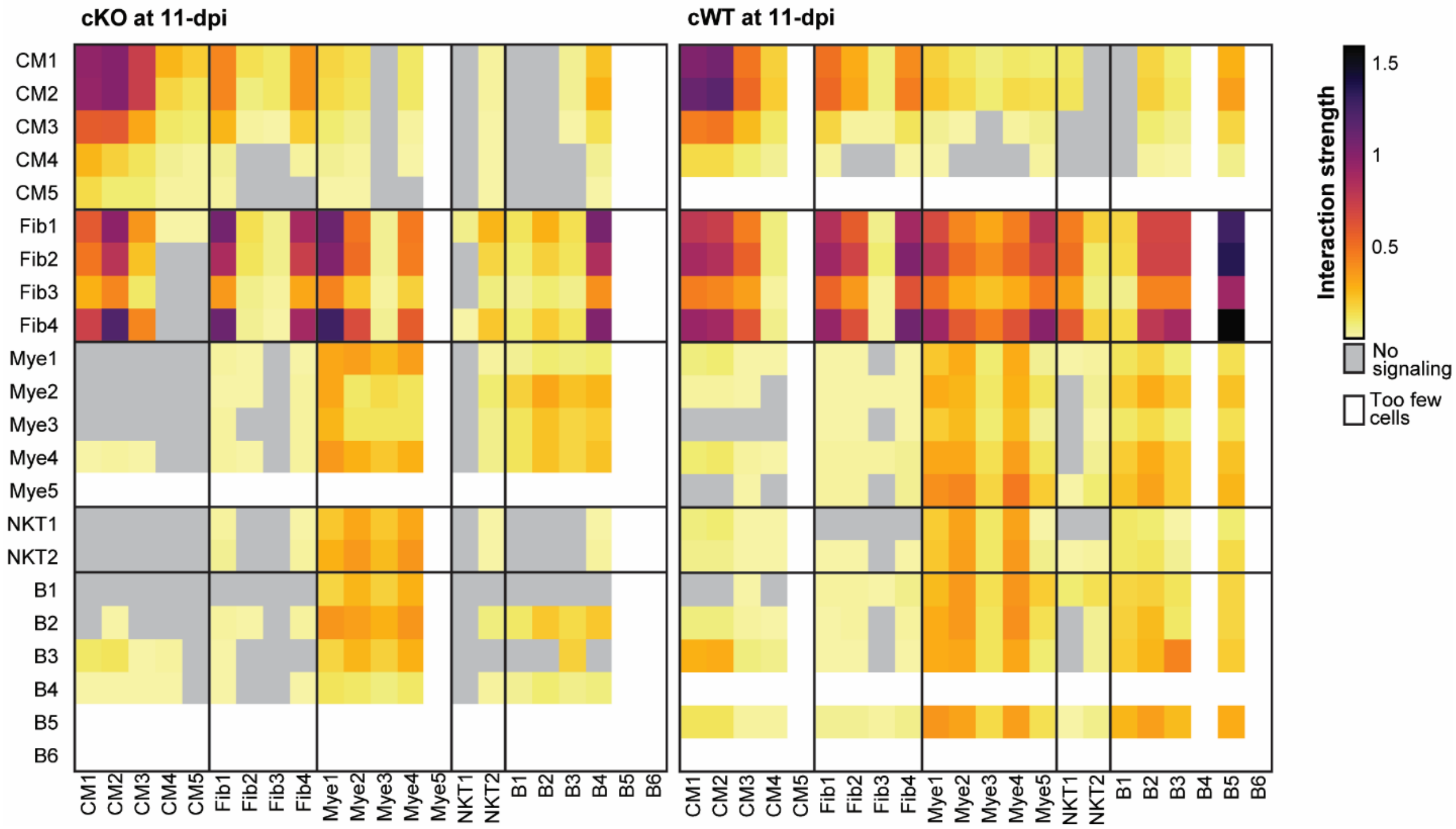

Supplementary Figure S16: cKO hearts show reduced cell-cell communication in comparison to cWT samples. Heatmap of the cell-cell communication interaction strength between all subcluster populations of interest at 11-days post injection (dpi) in cKO (left) and cWT (right). Interactions where no communication was identified (gray) and where too few cells were available to perform communication predictions (white) are shown.

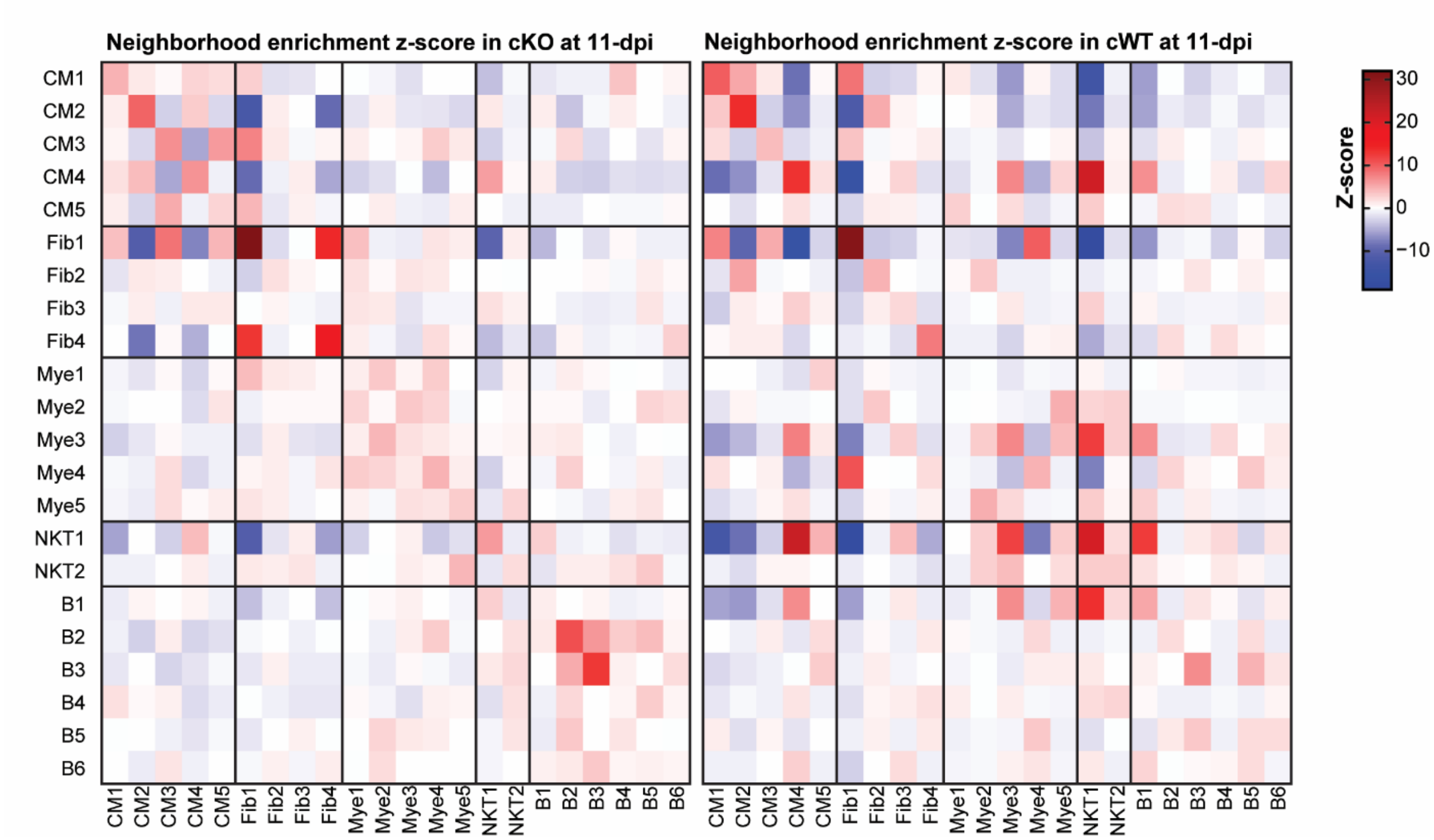

Supplementary Figure S17: Spatial reorganization of cells in cKO hearts promotes aberrant cell-cell communication. (A) Heatmap of *z*-score neighborhood enrichment for cell type subpopulations in 11-days post injection (dpi) cKO (left) and cWT (right) ventricles based on spatial transcriptomics.

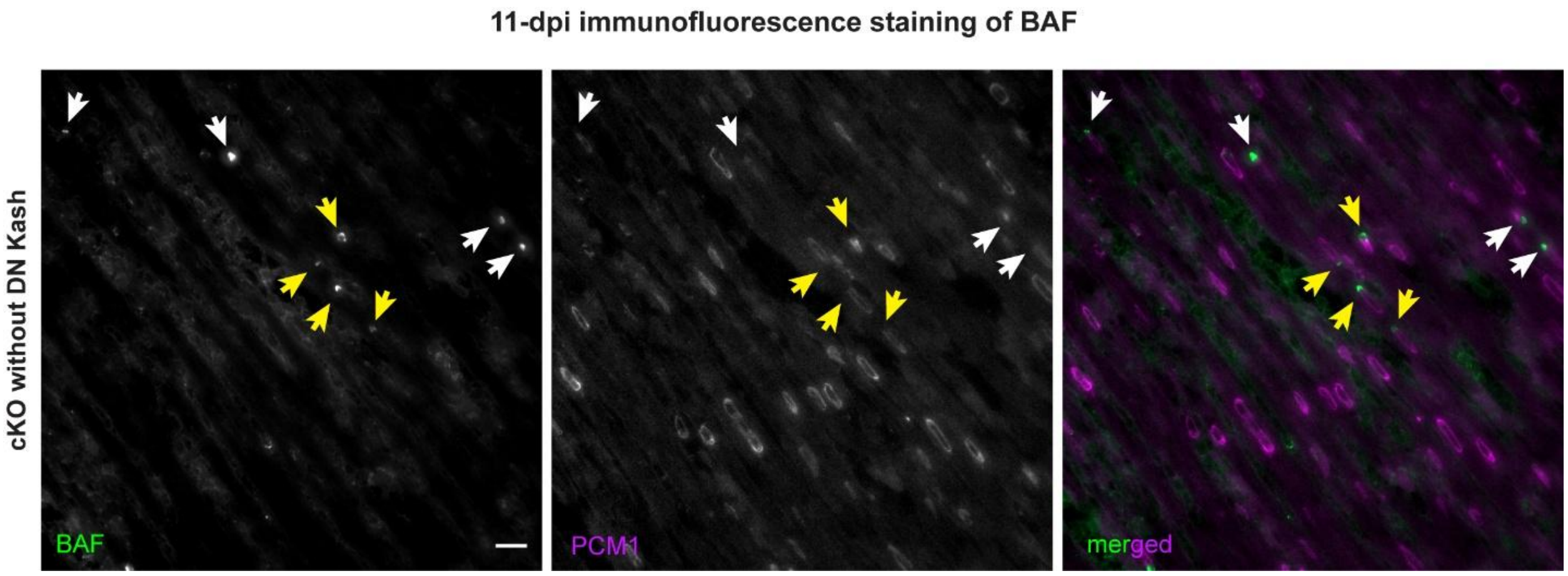

Supplementary Figure S18: *Lmna*-deficient cardiomyocytes without LINC complex disruption have increased rates of nuclear envelope rupture. Representative images of cardiac tissue sections from cKO mice without LINC complex disruption at 11-dpi with antibodies against the nuclear envelope rupture marker BAF (green) and the cardiomyocyte specific marker PCM1 (magenta). Scale bar 20 µm. Arrows indicate representative cardiomyocytes positive for nuclear envelope rupture. Yellow arrows point to the nuclei depicted in the main text figure 6B.

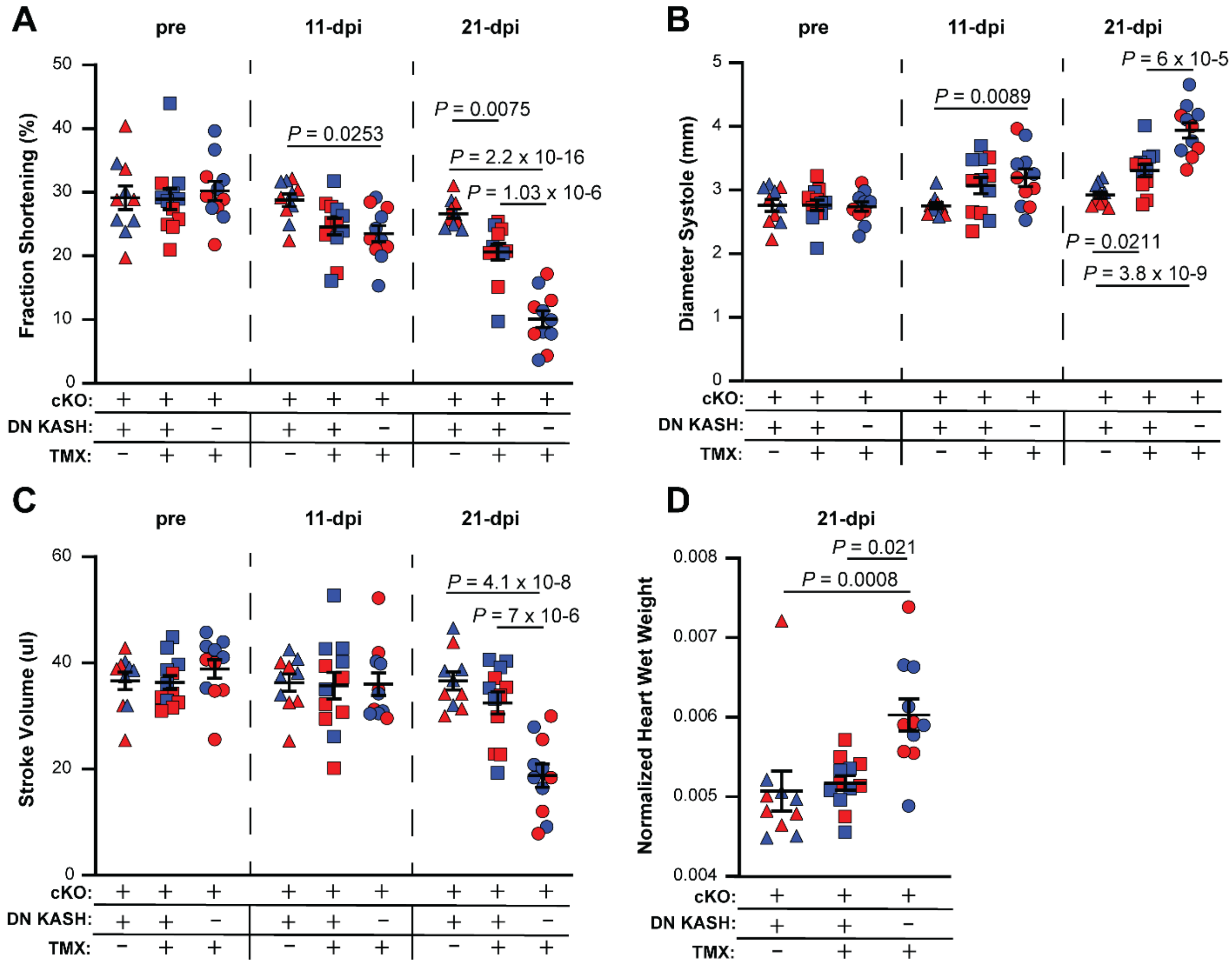

Supplementary Figure S19: LINC complex disruption rescues cardiac function in cKO mice. (A-D) Functional cardiac measurements of left ventricles from cKO mice with (+) and without (–) cardiomyocyte-specific LINC complex disruption using a dominant negative nesprin KASH domain construct (DN KASH) induced by tamoxifen (TMX) injection. **(A)** Fractional shortening (given as percentage), **(B)** diameter systole, and **(C)** stroke volume were measured by echocardiography either before TMX injection (pre, triangles), or at 11-days post injection (dpi) (squares) and 21-dpi (circles). **(D)** Heart wet weight normalized to mouse body weight was assessed at 21-dpi. Statistical tests: **(A-C)** Tukey Honest Significant Difference of repeated measurements two-way ANOVA test and **(D)** Dunn's Honest Significant Difference of Kruskal-Wallis. Color indicates sex of individual mice (red: female, blue: male). Error bars represent mean ± s.e.m. *N* = 10-12 animals per genotype.

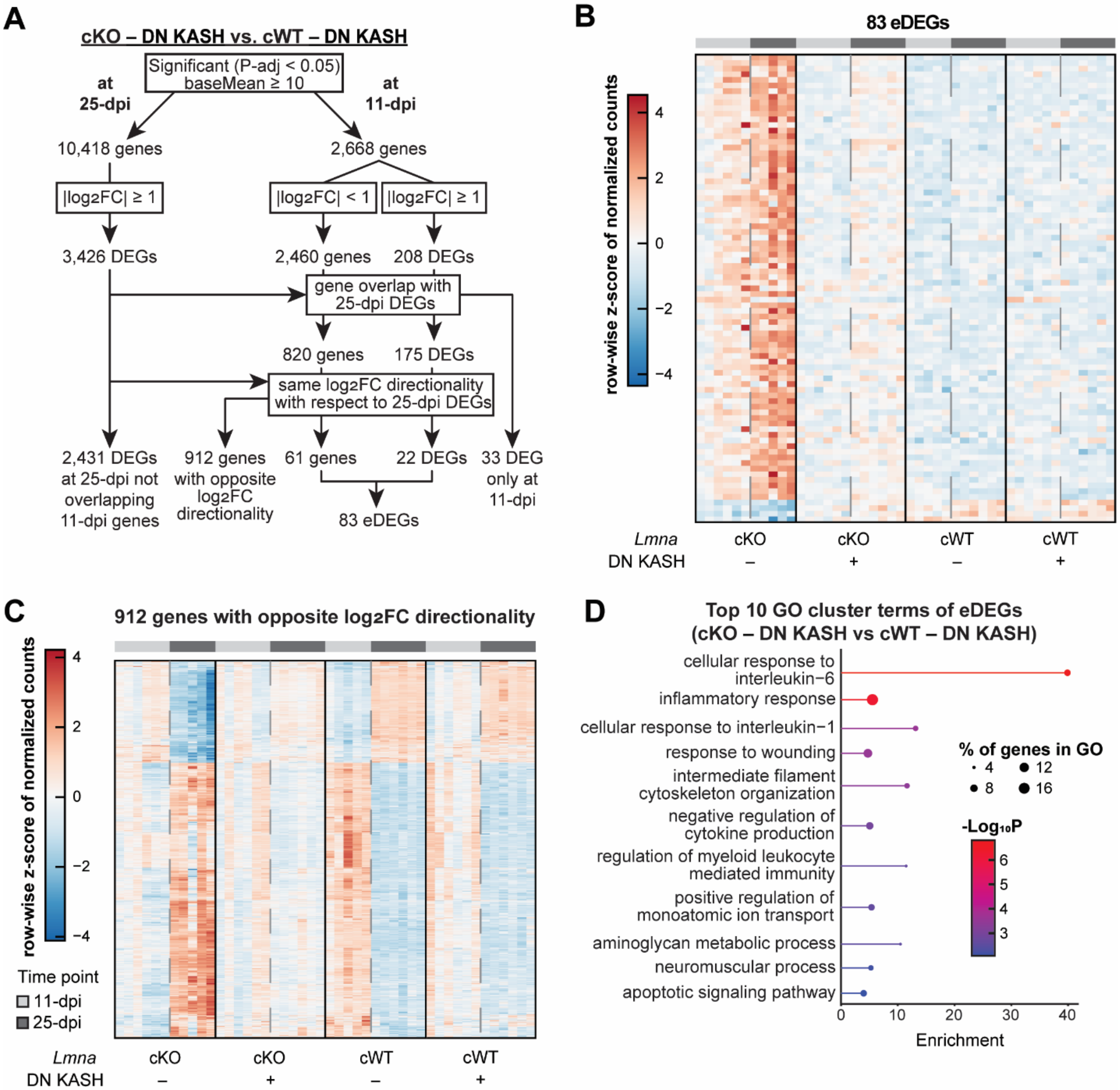

Supplementary Figure S20: eDEG pipeline and analysis in bulk RNA-seq data of left ventricular tissue for mice without LINC complex disruption. (A) Flow chart demonstrating the steps to identify emerging differentially expressed genes (eDEGs) from bulk RNA-seq data, based on log2-fold change (Log2FC) and adjusted *P*-values (*P*-adj) generated when comparing the gene expression between cKO and cWT samples both lacking LINC complex disruption (– DN KASH) at each timepoint. **(B)** Heatmap of row-wise *z*-score normalized read counts for the 83 eDEGs (cKO – DN KASH versus cWT – DN KASH) at 11- (light gray) and 25-dpi (dark gray). Columns, indicating individual samples, are separated by condition, where samples with (+) and without (–) cardiomyocyte-specific LINC complex disruption (DN KASH) are specified. **(C)** Heatmap of row-wise *z*-score normalized read counts for the 912 genes that had opposite log_2-fold change (log_2FC) directionality (cKO – DN KASH versus cWT – DN KASH) at 11- (light

gray) and 25-dpi (dark gray). Columns, indicating individual samples, are separated by condition, where samples with (+) and without (–) cardiomyocyte-specific LINC complex disruption (DN KASH) are specified. **(D)** Lollipop plot of the top 10 Gene Ontology (GO) cluster terms enriched in the 83 eDEGs (cKO – DN KASH versus cWT – DN KASH). Color indicates significance of enrichment and dot size represents the percentage of eDEGs contained within the term.

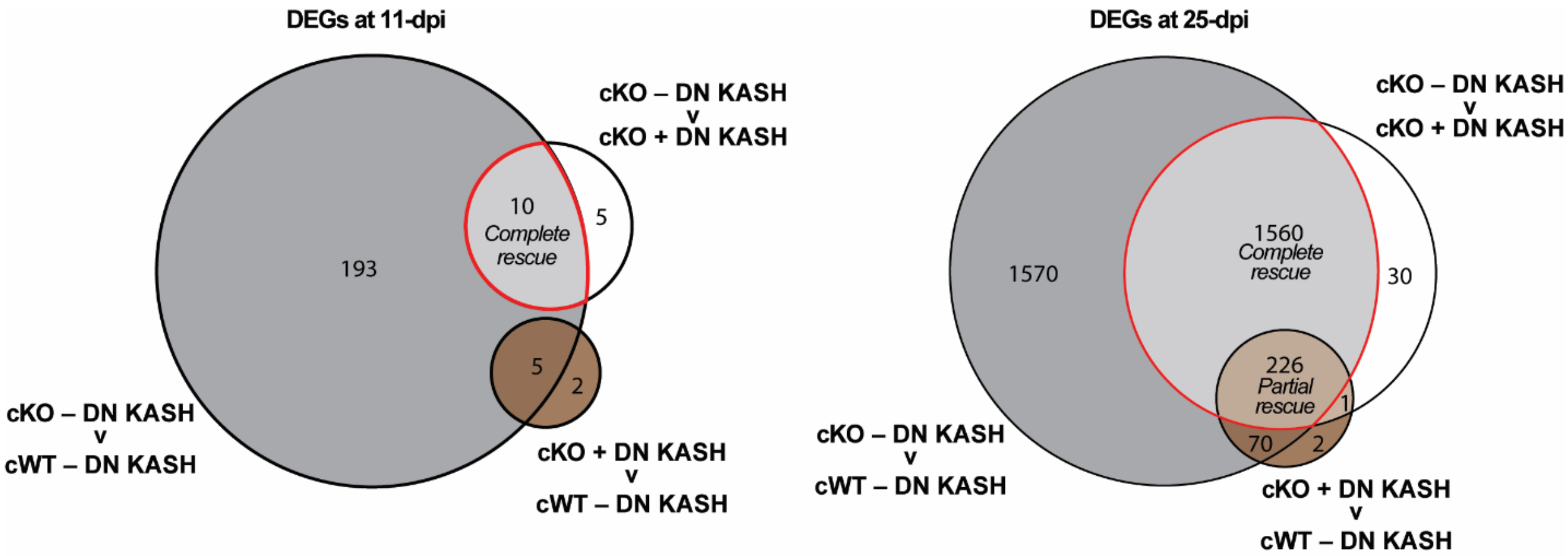

Supplementary Figure S21: LINC complex disruption partially rescues aberrant cKO gene expression. Euler plots showing the number of differentially expressed genes (DEGs) generated through three experimental condition comparisons at 11- (right) and 25-dpi (left). Overlaps between the comparisons indicate DEGs rescued by LINC complex disruption (red circle). Distinctions between *Complete rescue* and *Partial rescue* are also denoted.

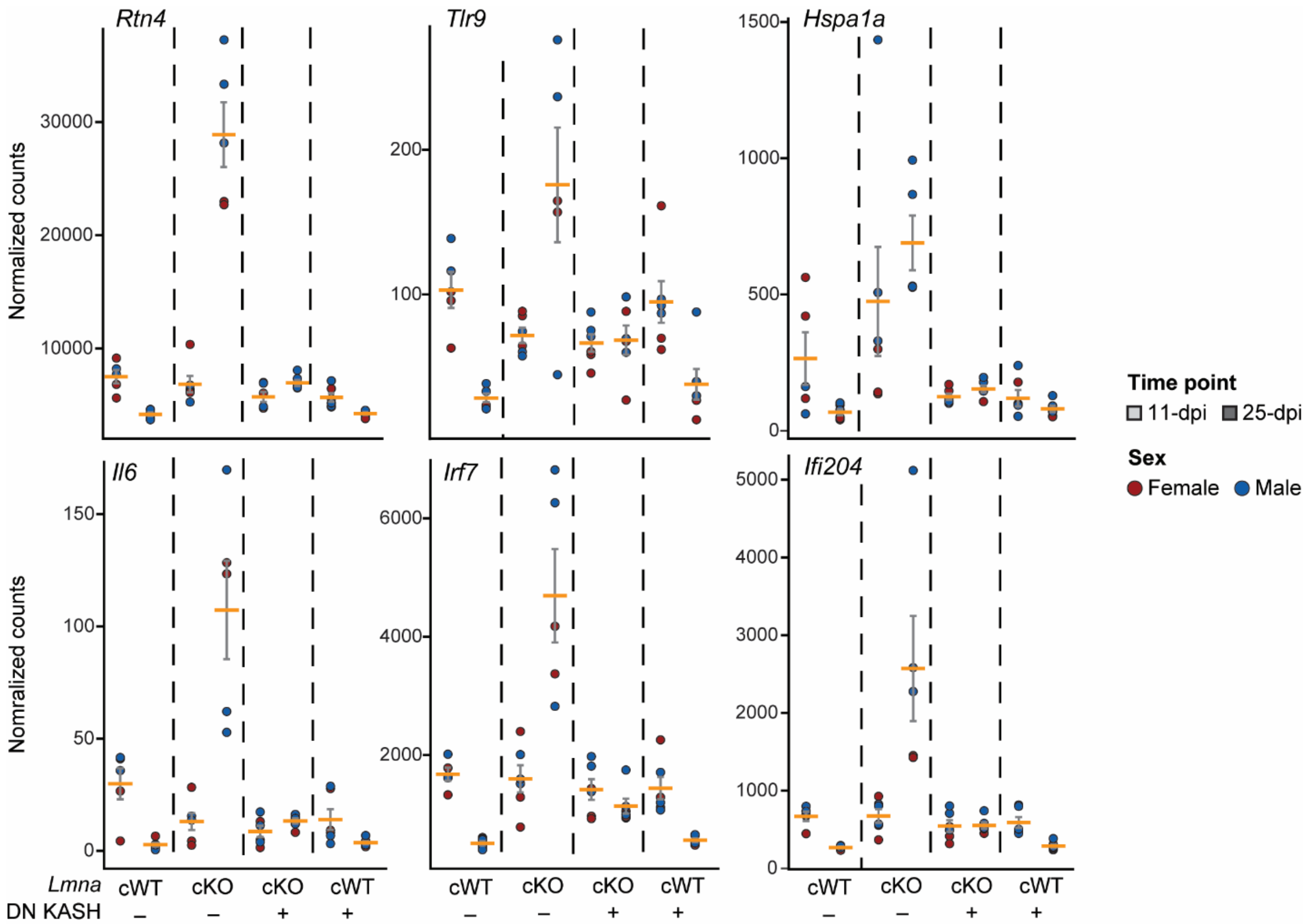

Supplementary Figure S22: LINC complex disruption restores normal expression of cytosolic DNA sensing genes. Quantification of normalized read counts for genes of interest involved in cytosolic DNA sensing within bulk RNA-seq samples across each experimental condition. cKO and cWT samples at 11- (light gray) and 25-days post injection (dpi) (dark gray) with (+) and without (–) cardiomyocyte-specific LINC complex disruption (DN KASH) are specified. Mean (orange line) and standard error of the mean (gray error bars) are represented. Color indicates sex of individual mice (red: female, blue: male). *N* = 4-5 animals per genotype.

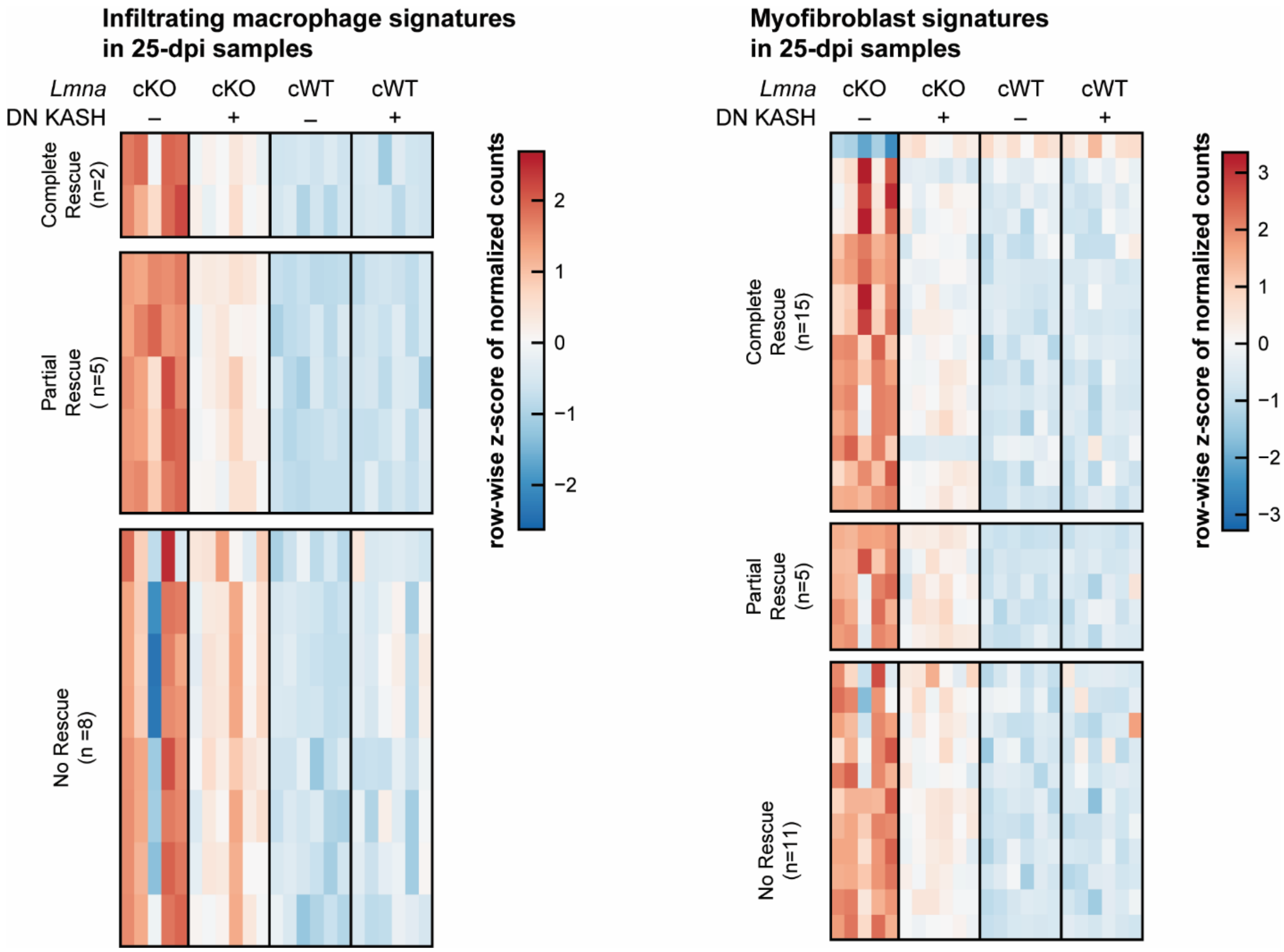

Supplementary Figure S23: Pathological transcriptional signatures show rescue by LINC complex disruption. (A) Heatmaps of row-wise z-scored normalized read counts of cKO – DN KASH vs. cWT – DN KASH DEGs representative of infiltrating macrophage (left) and myofibroblast (right) signatures across all experimental conditions. Heatmaps are split based on degree of rescue by LINC complex disruption induced by DN KASH expression.

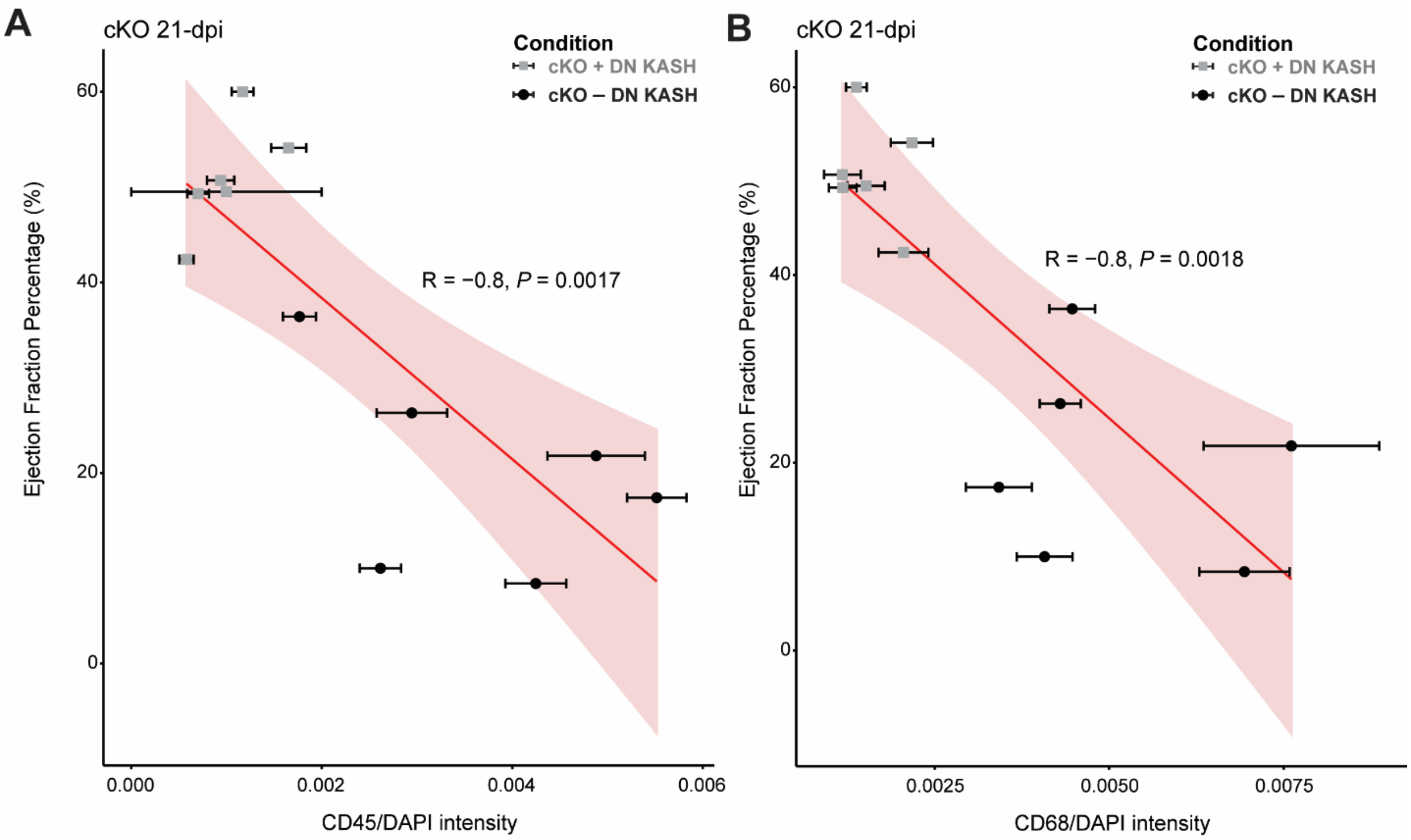

Supplementary Figure S24: Cardiac function inversely correlates to immune cell activation. Pearson correlation between the ejection fraction percentage and immunostaining intensity normalized to DAPI stain of left ventricle tissue for either **(A)** CD45 or **(B)** CD68 in cKO mice at 21-dpi. The presence (gray squares, cKO + DN KASH) or lack (black circles, cKO – DN KASH) of LINC complex disruption is indicated. *N* = 6 animals per genotype. Pearson correlation coefficient (R) and p-values for a linear fit (red) are displayed in the graphs.

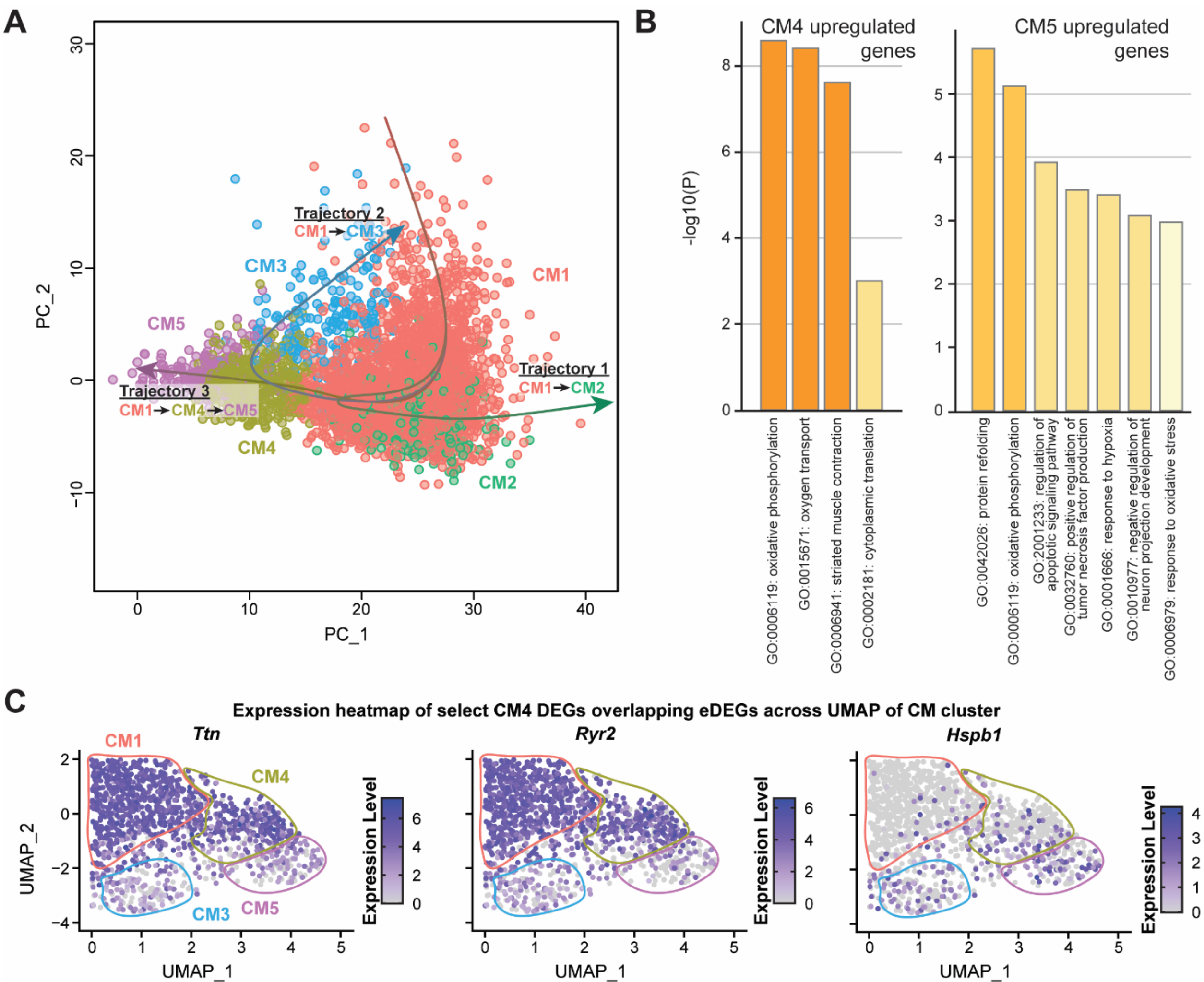

Supplementary Figure S25: LINC complex disruption restores normal expression of cytosolic DNA sensing genes. (A) PCA mapping of cardiomyocytes from the snRNA-seq dataset, colors indicate unique subpopulations. Three pseudotime trajectories (CM1 → CM2, CM1 → CM3, and CM1 → CM4 → CM5) are depicted by arrows, indicating that the CM1 population represents an earlier cardiomyocyte state that can progress along three distinct trajectories. **(B)** Barplot showing the significance of gene ontology (GO) term enrichment for genes upregulated in cardiomyocyte subpopulation CM4 (left) and CM5 (right). **(C)** UMAP plot of integrated cardiomyocyte subpopulations (outlined by color) for CM4-specific DEGs overlapping eDEGs (*Ttn* – left, *Ryr2* – middle, *Hspb1* – right). Heatmaps indicate normalized read counts for each gene.

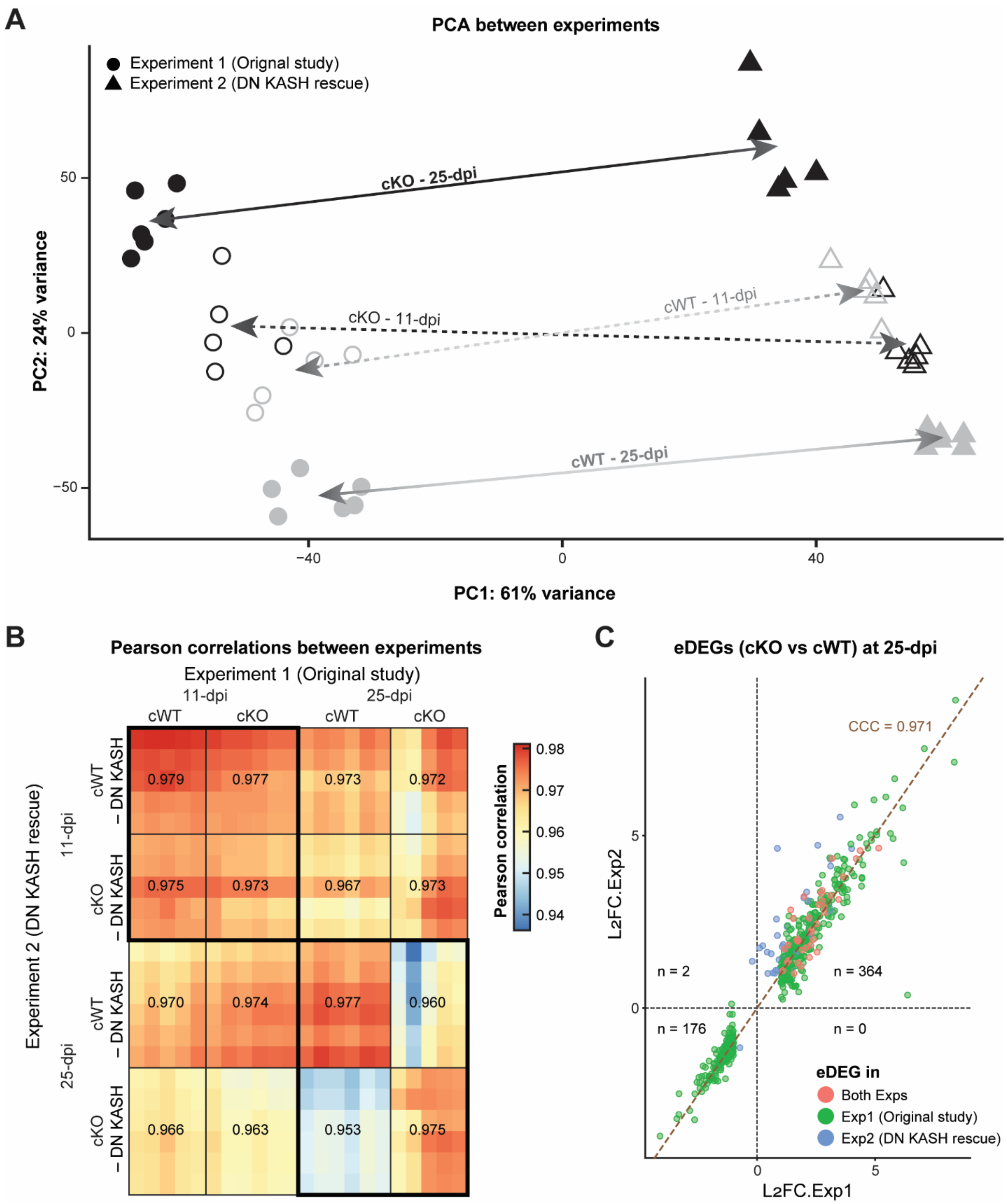

Supplementary Figure S26: Early disease stages are characterized by greater biological variability. (A) Principal component analysis (PCA) plot of bulk RNA-seq data for cWT (gray) and cKO (black) samples at 11- (open shape) and 25-dpi (filled shape). Samples from both the first (circle, GSE269451 used in Figure 2) and second (triangle, GSE304346 used in Figure 6) experiments are included. Arrows point to the center of

centroids for each condition, joining the same experimental condition across experiments. **(B)** Heatmap of Pearson correlations of bulk RNA-seq datasets between the first (x-axis) and second (y-axis) experiments. Rows and columns represent individual samples. Average Pearson correlation values are indicated by condition. Thick borders indicate similar timepoints**. (C)** Scatter plot of $\log_2$-fold change (L2FC) for cKO vs. cWT eDEGs in the first (x-axis, used in Figure 2) and second (y-axis, used in Figure 6) experiment. The experiment in which eDEGs were called are indicated by color. The concordance correlation coefficient (CCC) and the $y = x$ identity line are shown in brown.

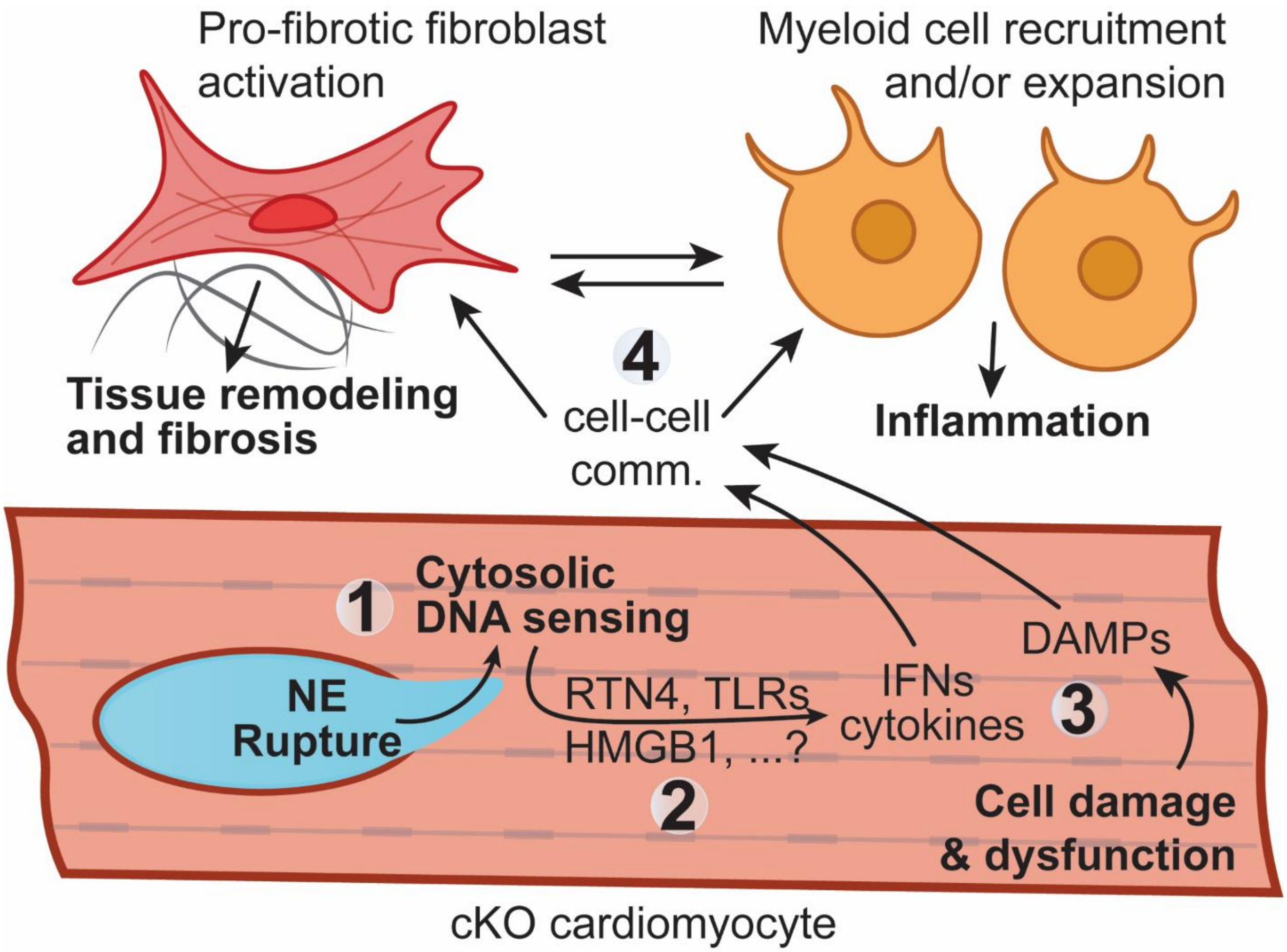

Supplementary Figure S27: Cytosolic pattern recognition receptor signaling downstream of nuclear envelope rupture drives *LMNA*-DCM pathology. (1) Mechanical stress on the nuclear envelope (NE) leads to rupture in structurally compromised nuclei of cKO cardiomyocytes (notably subpopulations CM3 and CM5), resulting in exposure of nuclear DNA to the cytoplasm, either in the form of chromatin protrusions or cytoplasmic leakage of nuclear DNA fragments. **(2)** Cytosolic DNA is detected by cGas/STING-independent cytosolic nuclei acid sensors, such as Toll-like receptors (TLRs) regulated by RTN4 and HMGB1, thereby activating cytoplasmic pattern recognition receptor (PRR) signaling. **(3)** PRR activation triggers the production of interferons (IFNs) and cytokines by cKO cardiomyocytes. Concurrently, cardiomyocytes release damage-associated molecular patterns (DAMPs) as a consequence of cellular stress and injury. **(4)** These signaling molecules mediate aberrant cell-cell communication to fibroblast (Fib1) and myeloid cells (Mye1), promoting pro-fibrotic and inflammatory signaling cascades, respectively. Crosstalk between these fibroblasts and myeloid cells further amplifies fibrosis and inflammation in the heart, ultimately impairing cardiac function. LINC complex disruption attenuates this pathological cascade by substantially reducing the frequency of NE rupture events in cKO cardiomyocytes.

Supplementary Table(s)

Top 25 predicted upstream regulators of eDEGs at 11-dpi

Upstream Regulator	Molecule Type	Predicted State	Activation z-score	$-Log_{10}$(*P*-value) of Overlap	Top 10 Differentially Expressed Target Molecules in Dataset
Ifng	cytokine	Activated	6.466	30.74	Nmrk2, Cd24a, H2ac6, Hmox1, Hspa1a, Lgals3, Ccl7, Mlana, Mafa, Dbp
Tnf	cytokine	Activated	6.071	15.95	Hmox1, Hspa1a, Lgals3, Serpina3n, Serpinb1a, Ccl7, Mlana, Mafa, Klf5
Il1b	cytokine	Activated	5.073	16.02	Cd24a, H2bc8, Hmox1, Hspa1a, Serpina3n, Ccl7, Mafa, Cdkn1a, S100a3, Dbp
INTERFERON ALPHA (family)	group	Activated	4.968	12.02	Hmox1, Hspa1a, Lgals3, Serpinb1a, Cdkn1a, Tnfsf18, Cxcl2, Il1rn, Atf3, Oas1g
Il1a	cytokine	Activated	4.662	8.92	Hmox1, Serpina3n, Cdkn1a, Cxcl2, Ccl2, Il1rn, S100a10, Atf3, Sprr1a, Ccl4
Myd88	other	Activated	4.634	8.41	Hmox1, Gdf15, Tnfrsf12a, Cxcl2, Ccl2, Cebpd, Il1rn, Nppb, Maff, Ccl17
Tlr4	transmembrane receptor	Activated	4.632	11.59	Hmox1, Ccl7, Nr4a2, Cxcl2, Ddit3, Ccl2, Cebpd, Nppb, Atf3, Ccl4
App	other	Activated	4.375	10.26	Hmox1, Hspa1a, Crabp2, Lgals3, Ccl7, Cdkn1a, Gdf15, Nr4a2, Shisa3, Cxcl2
Sting1	ion channel	Activated	4.329	14.47	Cxcl2, Ddit3, Ifi27l2a, Oas1g, Oas3, Oas2, Fcgr4, Ccl4, Pnp, Irf7
NFKB (complex)	complex	Activated	4.292	14.27	Slc2a5, Cd24a, Hmox1, Serpina3n, Ccl7, Cdkn1a, Gdf15, Cgref1, Cxcl2, Dbp
Il6	cytokine	Activated	4.274	7.71	Hmox1, Serpina3n, Cdkn1a, Tnfrsf23, Hpx, Tnfrsf12a, Cxcl2, Ccl2, Cebpd, Il1rn
Irf7	transcription regulator	Activated	4.262	9.11	Ccl2, Oas1g, Oas3, Oas2, Ly6a, Ube2l6, Irf7, Ifitm3, Mx2, Phf11b
Irf3	transcription regulator	Activated	4.235	13.75	Cdkn1a, Ccl2, Ifi27l2a, Oas1g, Oas3, Oas2, Ccl4, Pnp, Ube2l6, Irf7
TP53	transcription regulator	Activated	4.212	15.38	Peg10, Hmox1, Hspa1a, Lgals3, Serpina3n, Psat1, Klf5, Cdkn1a, Ddit4l, Dbp
Csf2	cytokine	Activated	4.189	8.94	Hmox1, Cdkn1a, Ccl25, Gdf15, Nr4a2, Ifrd1, Cxcl2, Ddit3, Ccl2, Il1rn
Agt	growth factor	Activated	4.102	19.62	Hspa1b, Gabrb3, Hmox1, Nptx2, Hspa1a, Crabp2, Lgals3, Serpina3n, Ccl7, Klf5
Tgfb1	growth factor	Activated	4.095	20.08	Hmox1, Hspa1a, Lgals3, Serpina3n, Psat1, Serpinb1a, Ccl7, Cdkn1a, Gsta2, Dbp
LDL (complex)	complex	Activated	4.088	16.87	Hmox1, Ccl7, Cdkn1a, Star, Nr4a2, Cxcl2, Ccl9, Ddit3, Ccl2, Il1rn
ERK1/2 (family)	group	Activated	4.056	8.21	Cdkn1a, Star, Gnrh1, Cxcl2, Ddit3, Ccl2, Ccn2, Ccl4, S100a6, Rhoc
Irgm1	enzyme	Inhibited	-4.042	11.58	Ccl2, Ifi27l2a, Oas1g, Oas3, Oas2, Apol9a, Irf7, Ifitm3, Mx2, Ifi204
Apoe	transporter	Inhibited	-4.048	11.14	Hspa1b, Hmox1, Hspa1a, Serpina3n, Cxcl2, Ddit3, Ccl2, Il1rn, Ccn2, Atf3
Irf2bp2	transcription regulator	Inhibited	-4.094	9.55	Hmox1, Hcn1, Serpina3n, Ccl9, Scimp, Fcgr4, Cd14, Mybl1, Mmp3, Pilra
Ttc39aos1	other	Inhibited	-4.319	17.16	Ccl7, Ccl2, Oas3, Oas2, Ccl4, Ly6a, Apol9a, Ube2l6, Irf7, Ifitm3
Trex1	enzyme	Inhibited	-4.349	16.36	Cdkn1a, Ifi27l2a, Panx1, Oas1g, Oas3, Oas2, Ly6a, S100a6, Ube2l6, Gpx1
Cited2	transcription regulator	Inhibited	-4.61	9.78	Ifrd1, Cxcl2, Ccl2, Il1rn, Pou3f1, Oas1g, Oas3, Scimp, Gadd45b, Fcgr4

Supplementary Table S1: Cytosolic DNA sensing signaling proteins predicted as upstream regulators of eDEGs. Top 25 upstream regulators, identified by Ingenuity Pathway Analysis (IPA), predicted to be activated or inhibited based on 11-dpi eDEG expression differences between cKO and cWT samples. Top 10 most differentially expressed eDEGs targeted by predicted upstream regulators are displayed.
